# Exploration of Chemical Space Through Automated Reasoning

**DOI:** 10.1002/anie.202417657

**Published:** 2025-01-05

**Authors:** Judith Clymo, Christopher M. Collins, Katie Atkinson, Matthew S. Dyer, Michael W. Gaultois, Vladimir V. Gusev, Matthew J. Rosseinsky, Sven Schewe

**Affiliations:** ^1^ Department of Computer Science University of Liverpool, Ashton Building Ashton Street Liverpool L69 3BX United Kingdom; ^2^ Department of Chemistry University of Liverpool Crown Street Liverpool L69 7ZD United Kingdom; ^3^ Leverhulme Research Centre for Functional Materials Design Materials Innovation Factory Liverpool L7 3NY United Kingdom

**Keywords:** Artificial intelligence, explainable models, materials discovery, materials chemistry, automated reasoning

## Abstract

The vast size of composition space poses a significant challenge for materials chemistry: exhaustive enumeration of potentially interesting compositions is typically infeasible, hindering assessment of important criteria ranging from novelty and stability to cost and performance. We report a tool, Comgen, for the efficient exploration of composition space, which makes use of logical methods from computer science used for proving theorems. We demonstrate how these techniques, which have not previously been applied to materials discovery, can enable reasoning about scientific domain knowledge provided by human experts. Comgen accepts a variety of user‐specified criteria, converts these into an abstract form, and utilises a powerful automated reasoning algorithm to identify compositions that satisfy these user requirements, or prove that the requirements cannot be simultaneously satisfied. In contrast to machine learning techniques, explicitly reasoning about domain knowledge, rather than making inferences from data, ensures that Comgen's outputs are fully interpretable and provably correct. Users interact with Comgen through a high‐level Python interface. We illustrate use of the tool with several case studies focused on the search for new ionic conductors. Further, we demonstrate the integration of Comgen into an end‐to‐end automated workflow to propose and evaluate candidate compositions quantitatively, prior to experimental investigation. This highlights the potential of automated formal reasoning in materials chemistry.

## Graphical abstract

In this work we develop an automated reasoning method, COMGEN to determine valid compositions for a given set of domain expert defined constraints. We demonstrate the method in an automated workflow with crystal structure prediction and properties models to locate and predict new potential Li‐ion solid state electrolytes.

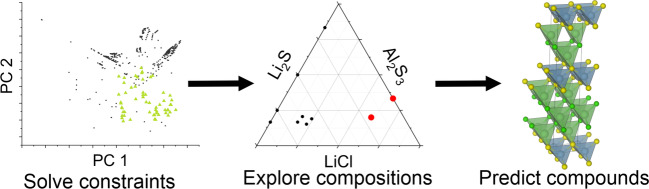

## 1 Introduction

The discovery of new materials drives advances in technology through new properties or property combinations that enhance performance in applications. Materials discovery also expands our understanding of chemical bonding through the realisation of new structures, thus opening unexplored paths to property generation and control. Through progress in understanding of crystal chemistry, enabled by advances in structural science, researchers have been able to target new materials by connecting their chemistries and in particular their compositions to those of known materials, whose structures have been determined and are reported in the databases. However, it remains challenging to identify compositions for synthesis that are not closely associated with known materials. This is because bonding in solids is less well understood than in isolated molecules, and also because of the complex structural relationships between the starting materials and products due to the need for mass transport in solid state reactions.

The vast size of the potential space of new materials is well known. For example, over 16 billion combinations of the form $A_{w}B_{x}C_{y}D_{z}$ are possible where *w,x,y,z* are non‐negative integers up to 8.^[1]^ When allowing for more distinct elements or continuous variation of stoichiometric factors the number of possible compositions grows exponentially and explicit enumeration of all candidates becomes infeasible. The use of the Element Mover's Distance (ElMD)^[2]^ to map chemical space and known materials within it has further demonstrated that existing knowledge is highly clustered, with large areas remaining relatively under‐explored. As compositions well separated from previous knowledge potentially offer fresh insight and enhanced functionality, it is important to consider ways to support decisions made by synthetic chemists to undertake experimental work in regions of chemical space that are not closely connected to current knowledge. Addressing this at scale remains a grand challenge. Solutions must tackle the high dimensionality of the problem (resulting from the number of available elements), the diversity in types of bonding present and resulting wide range of potential crystal structures, and the continuous variation of composition that is possible in solid solutions.

A common approach to exploring chemical space is to enumerate possible compositions and then filter these using various methods. One such tool is SMACT,^[1]^ in which possible compositions are filtered according to rules based on abstract scientific knowledge or on analysis of existing materials. For example, relative frequency of oxidation states in experimentally observed materials is used to assess the probability of new compositions based on the combination of oxidation states that they would require.^[3]^ Machine learning models that predict formation energy and other material properties from compositions (for example [4–6]) can similarly be used to filter candidate compositions. However, any approach that requires explicit enumeration of compositions is limited by the sheer number of candidates, as noted above, and must therefore restrict either the number of elements permitted or the stoichiometry or both.

Instead of enumerating compositions, candidate materials may be generated using prototype‐based substitution into known structures. Candidates can once again be filtered and ranked according to learned models or computational tools that predict their formation energies. Such an approach has been recently used to propose millions of materials, some of which have subsequently been successfully synthesised.[^7^, ^8^] However, the method has been criticised for primarily proposing materials that do not exhibit true novelty and are instead highly similar to existing materials.^[9]^ Another recent approach employs diffusion models to generate possible crystal structures.[^10^, ^11^, ^12^] As with all learned models, ensuring true novelty of generated structures is a challenge. In addition, current diffusion models are limited in the level of control available to the human scientist, who is unable to specify in detail what conditions the generated materials should satisfy.

In order to both extend human control and address the problem of explicitly enumerating candidate compositions, we present a tool to generate compositions according to user‐specified constraints, which can be inspected and iteratively updated. The tool, Comgen, allows users to apply their domain expertise to prioritise targets for experimental synthesis. An important benefit of exploring compositions using constraints is that the chemical space is navigated consistently and robustly at a scale beyond that of human evaluation, tackling the problem of dimensionality while using expert input, and with greater efficiency than a brute force approach. To achieve this, Comgen abstracts chemical constraints into a logical form and uses state‐of‐the‐art methods from computer science that capture and automate domain reasoning, akin to how a human domain expert approaches the task, rather than making statistical inferences over large volumes of data points. Instead of simply checking each possible composition independently against a constraint, the reasoning algorithm is able to learn why a constraint has been violated and avoid considering other candidates that fail the constraint for the same reason. Reasoning about constraints in this way prevents explicitly enumerating all possibilities and also allows for solutions with non‐integer stoichiometries, which may be especially relevant for disordered or doped materials. Because multiple constraints can be combined, we can build up complex requirements that would be impossible for a human researcher to reason about.

The constraints used by Comgen are designed to capture chemical reasoning, such as the inclusion of particular elements or polyatomic units to encourage desired properties, the requirement for particular size relationships or anion to cation ratios between components to promote structural features, or adherence to appropriate bonding rules, such as charge neutrality for compounds treated as ionic. By including the ElMD to reported materials in the constraint system, Comgen generates candidate compositions for synthesis at desired distances from known chemistry, allowing for a simple calibration of novelty and accessibility of the results. Because the constraints used are connected to chemical decisions, Comgen presents the researcher with the unbiased consequences of their requirements, to inform subsequent choices regarding experimental targets. Further, results are explainable since all outcomes are fully traceable to the expert selection of constraints and are not subject to bias beyond that implicit in the constraints themselves. Crucially, results are not subject to the bias common to all machine learning techniques, which strongly promotes prediction of materials that are very similar to previously reported materials.

Comgen may be used in combination with crystal structure prediction,[^13^, ^14^, ^15^] and composition‐based property predictions[^16^, ^17^] for further screening generated compositions. This gives the flexibility to combine chemical knowledge and understanding with the advanced statistical analysis afforded by machine learning. We demonstrate the use of Comgen in such a combined workflow with the computation of energetic stability of the new materials through automated convex hull calculation, enabling quantitative information to be returned to the chemist following an enquiry formulated according to their understanding.

Comgen is provided for use without composition restriction, or with the bonding criteria for ionic materials, a large and important class, for example in energy technologies.[^18^, ^19^, ^20^] The rules‐based approach for composition generation is readily extended to other types of chemical bonding.

## 2 Results and Discussion

### 2.1 Comgen Description

We automate the exploration of composition space by defining the task of suggesting compositions for further investigation as a constraint satisfaction problem. Valid compositions are conceptualised as a list of element quantities that sum to one. We restrict attention to compositions which are plausible (e.g., we require charge neutrality in ionic materials) and further constraints are used to bias solutions towards compositions which are most likely to have desirable properties or are highly novel.

The constraint system is automatically built based on the user's choice of options and any inputs provided. Chemical constraints are abstracted to a logical form and then passed to a state‐of‐the‐art Satisfiability Modulo Theories (SMT) solver Z3,^[21]^ which either returns a solution or determines that this combination of constraints cannot be satisfied. A review of SMT solving and the logical system used by Comgen is provided in the Methods section.

Solutions from the SMT solver are assignments to the variables used in the constraint system. The solutions are interpreted in light of the chemical meaning of the constraints and transformed into a set of compositions that are returned to the user. All of this functionality is available via a user‐friendly API so that the user is never required to directly interact with the full mathematical form of the query.

There are several benefits to using a constraint‐based approach to composition generation over enumerating possibilities. Firstly, large parts of chemical space may be explored simultaneously. It is not necessary to consider only one phase space at a time, and the constraints may be modified on subsequent calls to the solver, allowing for a dynamic exploration. The efficient exploration is enabled by the ability of underlying algorithms to learn new constraints by continually combining existing constraints. Instead of considering one composition at a time, this approach allows the same logical deduction to be applied to multiple possible solutions and across distinct areas of chemical space at once. Secondly, an enumeration approach would typically need to define a grid over the phase space of interest. This may exclude valid compositions. The SMT solver used by Comgen is able to reason about real numbers and therefore does not impose a fixed discretisation of the space under consideration. It is possible to set a minimum distance between any two generated compositions, but this does not force solutions onto a grid as enumeration would do. Finally, because we use a standard mathematical formalisation and a well‐supported constraint solver to build our tool, we automatically benefit from advances in fundamental computer science research. Automated theorem proving tools are the focus of a great deal of research and are continually improving^[22]^ and outperform naïve approaches in a wide range of complex real‐world settings.^[23]^

We now describe the available types of constraints; their formal mathematical definitions are provided in the Methods section. All the constraints described here are templates and are customised for each individual query. Not every kind of constraint needs to be used in every query, and each type of constraint may be included multiple times. This flexibility means that queries can be tuned to allow for more wide‐ranging exploration or to search for precisely specified compositions. We demonstrate some use cases below in Sections 2.3 and 2.4.

#### 2.1.1 Element Selection

The set of possible elements must be provided to the composition generator tool at initialisation. Naturally, we require that all quantities are chosen by Comgen to be non‐negative, and that the total quantity of all elements must sum to one. The user can also set bounds on the number of distinct elements that can be selected from a subset of the permitted elements, and on the quantity of individual elements or across a set of elements. The constraints on element selection may be combined using Boolean connectives to build up more complex restrictions on the generated compositions. Similar constraints over the quantities of species, rather than elements, are also available.

#### 2.1.2 Charge Balancing

As well as selecting the quantity of each element to include, Comgen must select particular species to use. There are constraints specifying that the sum of oxidation states of the selected species must be zero. Comgen contains default values for possible oxidation states of each element in the form of elemental species that can be selected for inclusion in the composition. The default oxidation states may be replaced by user‐selected ones if required. In addition, it is possible to include poly‐atomic species such as peroxide or chlorite, and Comgen contains a default list of common poly‐atomic species.

We further enforce that the choice of species reflects the electronegativity of the element, so that selected positively charged species must have lower electronegativity than selected negatively charged species. Although in general one element is permitted to have multiple different oxidation states within a single composition, the possibility of a composition containing both a positively charged ion and a negatively charged ion of the same element is excluded. The charge balancing constraints are included by default when generating ionic compositions.

#### 2.1.3 Distance From Known Materials

We use the element mover's distance (ElMD) as a metric on the space of compositions.^[2]^ The ElMD computes the Wasserstein distance between compositions, which are represented as vectors of their element quantities, with elements ordered by their Pettifor number.^[24]^ It defines a metric space that can be used to represent the understanding of solid state chemistry that has underpinned and directed the selection of new compositions for synthesis. By adding constraints on the distance between a set of input compositions and the generated compositions, we can encourage the generation of either highly novel compositions (far from the given examples) or new compositions that are similar to known materials (close to the given examples), and therefore more likely to have similar desirable properties. Several ElMD constraints can be combined using Boolean connectives.

#### 2.1.4 Integer Stoichiometry

We may specify lower and upper bounds on the total of the stoichiometric coefficients when a composition is represented with a fully integer stoichiometry (e.g. Li_3_N as opposed to Li_0.75_N_0.25_). This is especially useful if we wish to perform crystal structure prediction on the generated compositions.

#### 2.1.5 Starting Materials

If we hope to synthesise new materials then the starting materials required to do this are an important consideration. Our tool allows the user to provide a list of existing compositions that may be used as the starting materials and require that any generated composition can be formed from these. Optionally, the user may also specify other metrics, such as costs or elemental scarcity, for each starting material and search for compositions formed from these with a bound on the total cost.

#### 2.1.6 Comparing Properties of Ions

Since we are working across composition space only, it is not possible to reason directly about the possible structure of any proposed materials. Such considerations can be introduced later in the workflow. However, we may attempt to bias the search towards compositions with particular structural features by constraining the relative sizes of constituent ions. Two types of constraints are available to achieve this. The user defines two sets of species. In the first approach, one species must be selected from each set to have non‐zero quantities in the generated composition, with the ratio of their sizes falling between some user‐specified bounds. In the second approach, the absolute difference in ionic radius is constrained. In this case we require that all pairs of species selected have a radius difference within the given bounds. Note that the values being compared could represent quantities other than the ionic radius, and therefore these constraints can be used to bound the relative values of other atomic or ionic properties.

### 2.2 Limitations

All of the constraints included in Comgen can be expressed over the theory of linear integer arithmetic (see Linear arithmetic in Methods Section). The constraints presented above give a flexible and expressive tool, and further constraints could be added within the theory of linear integer arithmetic to extend the functionality as required.

There are other constraints that might be desirable but cannot be expressed in this theory. For example, we could not constrain the variance of ionic radii using linear constraints, since calculating the variance requires the non‐linear square‐root function. By restricting attention to queries that can be expressed as a combination of linear constraints we have balanced flexibility and solving time. It would be possible to include more constraints by using a more expressive theory, as the SMT solver Z3 we have used supports non‐linear arithmetic of different types, including polynomial real arithmetic; however, this would substantially increase solving time in a problem‐dependent manner that is hard to predict.

### 2.3 Examples for the Identification of Li or Mg Ionic Conductors

Our case studies focus on the search for new ionic conductors for solid state batteries. Solid state lithium batteries have the potential to offer overall better performance than existing technologies, for example by allowing the use of lithium metal anodes and high voltage cathodes while removing flammable organic liquid electrolytes.^[25]^ However, a significant barrier to their realisation is the development of high performance electrolytes, having to balance properties such as ion transport, chemical stability with other battery components, and a low electronic conductivity. One path to resolving these often competing property requirements remains the discovery of new classes of solid state electrolytes. This also applies to battery technologies that rely on more abundant elements than Li, such as Mg.[^26^, ^27^, ^28^]

We present here various queries to demonstrate how Comgen can be used for exploring potential lithium ion conductors.

#### 2.3.1 Novel Lithium‐Containing Compositions

We begin by specifying the available elements, which were selected in order to avoid partially filled valence shells and also reducible transition elements in order to minimise the likelihood of electronic conductivity, as well as the oxidation states permitted for each: Li^+^, B^3+^, Al^3+^, Si^4+^, P^5+^, Mg^2+^, Zn^2+^, Ta^5+^, Sn^4+^, Ge^4+^, Ga^3+^, K^+^, Ca^2+^, Sr^2+^, Y^3+^, Zr^4+^, Ba^2+^, La^3+^, Gd^3+^, N^3−^, O^2−^, F^−^, S^2−^, Se^2−^, Te^2−^, Cl^−^, Br^−^, I^−^.

We require that lithium should account for at least 10 % of each generated composition. This ensures that compositions with only trace amounts of lithium are not returned. In addition to examples where candidate compositions were drawn from these elements subject to Element Movers Distance constraints, we also constructed queries that reflect a more detailed materials design process. Reflecting on the likely structural roles of the permitted elements in a new structure, we sub‐divided these elements into framework‐forming cations that would form extended covalent bonding networks with the selected anions, {B, Al, Si, P}, smaller non‐framework‐forming cations, {Mg, Zn, Ta, Sn, Ge, Ga}, larger non‐framework forming cations, {K, Ca, Sr, Y, Zr, Ba, La, Gd}, large anions {S, Se, Te, Cl, Br, I} and small anions {N, O, F}. With lithium forming a separate set, {Li}, this results in six disjoint sets. We specified that the compositions should contain exactly one element from each set.

The approach demonstrated here allows specific criteria regarding size and bonding to be built in to the search of chemical space, together with novelty‐based considerations. The selected categories reflect the following thinking: for lithium to be mobile, other elements are required to construct a suitable structural scaffold. Given the need for anions to balance charge, these can have different effects on the sites for lithium and pathways between them depending on their sizes. The non‐lithium cationic species may arrange the anions by forming a covalently‐bonded framework, typically involving tetrahedral coordination for the cation, as reflected in the first set. Such species can also direct both the specific nature of that framework and the coordination sites available for lithium by acting as larger, less covalently‐bonded extra‐framework cations that both appropriately compensate charge to enable a specific framework to form and determine the anion arrangement through their coordination requirements that differ from those of lithium. The second and third sets represent such cations, with more electropositive examples in the third set. The anion candidates are sub‐divided into the smaller first series anions (fifth set) and larger anions (fourth set). It would be possible to construct a range of queries on this basis, e.g., excluding one or both sets of non‐framework forming cations, only relying on one set of anions etc. This is a distinct way of using considerations of size and bond types from the identification of sets of ions with distinct radii and electronegativities discussed earlier.

We used the Liverpool ionic conductivity data set^[16]^ to prepare a list of known lithium containing compositions with good ionic conductivity. Although we are interested in discovering new materials with similar properties, we do not want to investigate compositions that are very closely related to known examples. To reduce the complexity of the query – which is directly correlated with the number of comparison compositions – and therefore also reduce solving time, we clustered compositions from the data set with a fixed radius (1.5) for each cluster and used representative compositions from these clusters to measure the distance from the generated compositions. We add constraints that require the generated compositions to have at least a distance of five, using the ElMD calculation, from these representatives. We chose five as a lower bound on the distance based on preliminary experiments, seeking a value to make the query strongly constrained but to still allow variety in the solutions (note that, because of the clustering step, the closest material from the full dataset can be a little less than five).

We present the results of this query in Table 1.

**Table 1:** Li containing compositions generated with an element movers distance (ElMD) of >5 from known Li ion conductors,^[16]^ using the constraints detailed in the “Novel Lithium Containing Compositions” section above. The composition of known conductors are provided in the same format as they appear in the database.

Composition	Nearest compound in database	ElMD
Li_0.2_K_0.05_Mg_0.217_Al_0.133_N_0.342_Br_0.058_	(Li_2_S)_0.4_((Ga_2_S_3_)_0.1_(GeS_2_)_0.9_)_0.6_	7.97
Li_0.2_K_0.05_Al_0.198_Sn_0.052_Br_0.224_N_0.276_	(LiI)_0.3_((Li_2_S)_0.3_((Ga_2_S_3_)_0.1_(GeS_2_)_0.9_)_0.7_)_0.7_	4.98
Li_0.203_La_0.05_Ge_0.064_P_0.183_N_0.449_Cl_0.05_	(Li_2_S)_0.4_((Ga_2_S_3_)_0.1_(GeS_2_)_0.9_)_0.6_	5.95
Li_0.2_K_0.056_Ta_0.058_Al_0.05_Br_0.578_O_0.058_	LiP_0.65_I_0.35_O_1.95_	5.01
Li_0.233_Zr_0.052_Ta_0.05_P_0.051_I_0.449_N_0.165_	Li_1.45_Al_0.45_Ti_1.55_(PO_4_)	5.01
Li_0.2_Zr_0.083_Ge_0.05_B_0.055_N_0.094_Cl_0.517_	Li_4_BN_3_H_10_	5.00
Li_0.283_K_0.05_Mg_0.189_P_0.078_N_0.35_Cl_0.05_	Li_2_SnS_3_	5.89
Li_0.2_K_0.164_Mg_0.174_P_0.072_N_0.34_Cl_0.05_	(Li_2_S)_0.75_ (P_2_S_5_)_0.25_	7.35
Li_0.2_K_0.053_Mg_0.296_P_0.051_N_0.35_Cl_0.05_	(Li_2_S)_0.4_((Ga_2_S_3_)_0.1_(GeS_2_)_0.9_)_0.6_	7.72
Li_0.211_Y_0.224_Al_0.053_Ge_0.075_Te_0.122_N_0.316_	Li_3.09_BO_2.53_N_0.52_	5.25
Li_0.2_K_0.076_Zn_0.289_P_0.05_S_0.05_N_0.335_	(Li_2_S)_0.4_((Ga_2_S_3_)_0.1_(GeS_2_)_0.9_)_0.6_	8.45
Li_0.2_K_0.176_Zn_0.206_P_0.052_S_0.05_N_0.316_	(Li_2_S)_0.75_ (P_2_S_5_)_0.25_	6.99
Li_0.3_K_0.05_Zn_0.23_P_0.05_S_0.05_N_0.32_	Li_2_SnS_3_	7.30
Li_0.2_Y_0.098_Ge_0.173_B_0.111_Te_0.085_N_0.333_	Li_7_Ge_3_PS_12_	5.41
Li_0.301_K_0.05_Ge_0.05_B_0.05_Se_0.052_F_0.498_	Li_2.88_PO_3.73_N_0.14_	4.81
Li_0.277_Zr_0.127_Ga_0.063_Si_0.05_S_0.267_N_0.214_	Li_2_MgZrO_4_	4.91

#### 2.3.2 Lithium Containing Compositions With Constraints on Starting Materials

The query above was modified to include the selection of starting materials shown in the Supporting Information. We removed the requirement for exactly one element from each set after Comgen proved that the query would otherwise be unsatisfiable. The compositions returned from this query were then passed to the crystal structure prediction program FUSE^[29]^ for structure prediction and energy calculation; the results are presented in the next section.

#### 2.3.3 Magnesium Ion Conductors by Distance

Given the scarcity of lithium and the surging demand for it, there is considerable interest in battery technologies based on more abundant elements. Magnesium is abundant and can provide intercalation‐based battery chemistry, opening the path to all solid state magnesium batteries if suitable electrolytes and cathodes can be found. Finding candidate Mg ion conductors is a challenge because of the higher charge and resulting lower mobility than lithium. Considering compositions that are in some way similar to ionic conductors based on lithium is one way of approaching this. We can now use the ElMD constraints to specify that we would like to find compositions that contain magnesium and are also close to at least one known lithium containing material known to have high ionic conductivity. The lithium ion conductors were again taken from the Liverpool ionic conductivity dataset, selecting the 55 entries that have a room temperature (15 to 35 °C) conductivity of at least 10^−3^ S cm^−1^. Results are shown in column A of Table 2.

**Table 2:** A) Magnesium containing compositions close (ELMD<3) to top performing lithium ion conductors; B) Magnesium containing compositions analogous to Li_6_PS_5_Cl; C) As B but allowing a lower proportion of Mg (still with 13 total atoms); D) As B but allowing less Mg and fewer total atoms.

A	B	C	D
Mg_2_Ba_5_S_5_BCl_4_F_3_	Mg_6_PN_5_S	Mg_4_PO_5_Br_3_	Mg_5_SiSe_2_N_2_O_2_
Mg_2_K_6_Ba_2_SiS_8_O	Mg_6_SiSeN_4_O	Mg_5_SiSe_7_	Mg_4_PS_4_NO
Mg_2_K_8_TeSeS_6_O	Mg_6_MnSeN_5_	Mg_4_SiO_4_BrCl_3_	Mg_4_SiSe_5_Te
Mg_2_K_8_BaS_6_BrCl	Mg_6_SiN_5_Br	Mg_5_SiO_2_S_5_	Mg_3_PClO_5_
MgBa_4_TeSeS_3_	Mg_6_SiTeSeN_4_	Mg_4_SiO_3_SCl_4_	Mg_3_SiBr_2_O_4_

#### 2.3.4 Magnesium Ion Conductors by Structural Analogy

As an alternative to the ElMD constraints we set constraints on the ratio between the ionic radius of magnesium and other elements in the composition, such that the ratios are close to those observed in a known lithium ion conductor. For example, we consider the argyrodite Li_6_PS_5_Cl. The composition contains six lithiums, one other cation, and six anions. The radius ratio of lithium to the other cation is approximately 1.7 and of lithium to the two anions around 0.5.

We search for compositions that contain six parts magnesium, one other cation, and six anions. The radius ratio of magnesium to the second cation is between 1.55 and 1.85, and to at least one of the anions is between 0.45 and 0.55 (permitting approximately a 10 percent variation from the observed ratios, consistent with the precision with which such radii are determined).

A sample of returned compositions is given in column B of Table 2. The outcome is noteable for the prevalence of heavily nitrogen‐rich compositions, driven by the higher charge of Mg. While there are highly conducting nitrides such as Li_3_N, the sensitivity of nitrides to oxidation might lead researchers to prefer targets with a lower nitrogen content, which a revised query that permits a Mg content of between 3 and 6 can afford. We note that argyrodites are reported with Li contents of less than six per formula unit,^[30]^ with the Cd^2+^‐containing argyrodite Cd_3.25_PS_5.5_I_0.5_ ^[31]^ emphasising the scope for lower cation contents with divalent ions. In one example of this approach, we retain the constraint of thirteen total ions in the formula as in the argyrodite, with at least six anions and at least one cation other than Mg, as presented in column C of Table 2. A second variant allows the total number of atoms to vary while fixing the number of anions and non‐Mg cations, with results presented in column D of Table 2.

### 2.4 Creation of an Automated Workflow With Crystal Structure Prediction and Machine Learning

A practical application of Comgen is to use it as part of an automated computational workflow to discover computational targets for experimental investigation based on quantitative assessment of the energetic stability and properties of the compositions identified by automated reasoning. In practice this then requires, for each generated composition, a crystal structure from which an energy can be computed and combined with previously known materials in order to compute an energy versus the convex hull (the composition‐energy surface created by stable phases) and a model to predict the desired property. In this work, we use the crystal structure prediction code FUSE^[13]^ to generate probe structures^[32]^ for this purpose (see Methods) and our previous classification model for the prediction of Li ion conductivity.^[16]^

**Figure 1:**
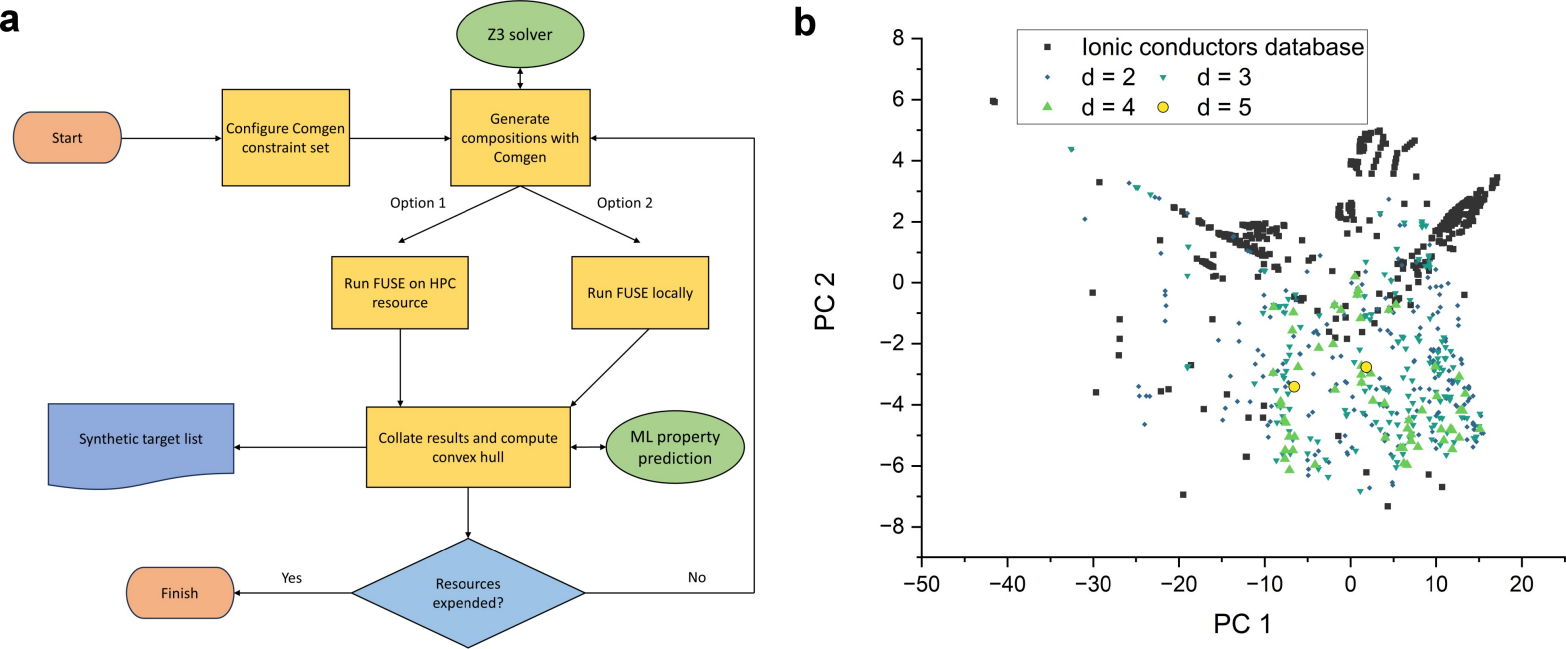
a) Flowchart outlining computational workflow implemented with python wrapper to use Comgen, built upon the Z3 theorem prover, and the structure prediction code FUSE to explore chemical space in this work. The machine learning model for ionic conductivity prediction is from Ref. [16]. b) Distribution of the Liverpool Li ion conductors database^[16]^ (black) plotted by ElMD metric,^[2]^ overlaid with the results of our search query for new Li ion conductors, selecting compositions at varying minimum distances from known phases. From these experiments, the results with the minimum distance to a known composition equal to 4 was taken forward for the crystal structure prediction runs presented within this work.

Comgen and FUSE were integrated into a combined workflow using a python wrapper as shown in Figure 1a. The first stage is to set up the constraints of the Comgen search query (Section 2.1), the wrapper then generates a population of compositions as requested by the user. The crystal structures for each of these is calculated by FUSE. Once all of the FUSE runs have completed, the results are collated and the convex hull is then computed. Typical stable target phases emerging from probe structure calculations will have an energy vs. the convex hull ≤45 meV atom^−1^ at zero Kelvin,^[17]^ with some metastable phases having energies above this. If the available resources have been exhausted computing the initial set of compositions (e.g. a target number of total compositions to generate, or total quantity of compute time spent) then the search will end. If resources remain, then the search will produce a subsequent generation of compositions, and the loop will repeat until resources have been spent.

To start our search, we modify the query “Novel lithium containing compositions” detailed previously (Section 2.3), using the option to select compositions from a subset of starting materials highlighted in the compositional data table supplied as Supporting Information, allowing Comgen to choose from a range of 15 elements, limited to compositions expressed as integers, with a maximum content of 20 atoms. To explore the number of possible solutions to our search, prior to FUSE calculations, the search for compositions was run exhaustively, varying the minimum distance^[2]^ of d=2, 3, 4 and 5 relative to the clustered Li ion conductors database (Section 2.3). These distances yielded 214, 162, 59 and 2 valid results, respectively (Figure 1b). The set of compositions with a minimum distance of 4 were selected to run, with the first 30 compositions to complete the basin hopping routine carried forward to convex hull calculations and ranking as synthetic targets (see Methods).

The compositions and energies above the convex hull are presented in Table 3, with all of the generated probe structures shown in the Supporting Information. Eight candidate compositions have an energy from the convex hull within 45 meV atom^−1^, with nine predicted to have a conductivity above 10^−4^ S cm^−1^.

**Table 3:** Compositions generated in Section 2.4 with a minimum distance of 4 by ElMD from representative compounds in the Li ion conductors database^[16]^ (giving a guarantee of at least distance 2.5 from all items in the full database), for which probe structures were generated. Each chemical formula is presented, as sorted by robocrystallographer.^[33]^ The energies from the convex hull calculated for the final structures generated by FUSE. Compositions shown in **bold** are predicted to have an ionic conductivity greater than 10^−4^ S cm^−1^ using the classifier machine learning model for ionic conductivity presented in Ref. [16], energies highlighted in **bold** indicate those within 45 meV atom^−1^ of being below the convex hull, probe structures within this energy window are typical for compounds to be targeted experimentally. The nearest reference composition to each of the generated compositions is presented as they appear in the database.

Composition	Energy from the convex hull (meV atom^−1^)	Nearest Li ion conductors data‐ base compound by ElMD^[2]^	Distance to nearest compound in database
$Li_{3}Si_{2}SCl_{9}$	66	$LiPO_{3}$	3.40
$Li_{5}ZrSiBr_{3}(O_{2}Cl{)}_{2}$	148	$Li_{3}PO_{4}$	4.13
$Li_{3}Si_{3}OBr_{8}Cl_{5}$	69	$LiBH_{4}$	3.52
$Li_{7}Al_{2}S_{2}Br_{8}Cl$	61	$Li_{2.88}PO_{3.73}N_{0.14}$	4.18
$Li_{6}AlBS_{2}OCl_{6}$	90	$Li_{2.88}PO_{3.73}N_{0.14}$	3.65
**LiAlOBrCl**	110	$(Li_{2}O{)}_{50}(Al_{2}O_{5}{)}_{5}(P_{2}O_{5}{)}_{45}$	3.38
$Li_{6}Zr_{2}SiO_{7}Br_{4}$	153	$Li_{3}P_{0.8}V_{0.2}O_{4}$	4.10
$Li_{3}ZrAl_{2}SO_{5}Cl$	191	$(LiI{)}_{0.3}((Li_{2}S{)}_{0.3}((Ga_{2}S_{3}{)}_{0.1}(GeS_{2}{)}_{0.9}{)}_{0.7}{)}_{0.7}$	4.20
$Li_{6}Al_{2}SBr_{4}(OCl{)}_{2}$	162	$Li_{2.88}PO_{3.73}N_{0.14}$	3.83
$Li_{6}Zr_{2}SiS_{2}O_{5}Cl_{4}$	192	$Li_{3}P_{0.8}V_{0.2}O_{4}$	4.30
$Li_{5}AlBr_{5}Cl_{3}$	**45**	$Li_{2.88}PO_{3.73}N_{0.14}$	4.31
$Li_{5}BCl_{8}$	47	$Li_{2.88}PO_{3.73}N_{0.14}$	4.23
$Li_{3}ZrAl_{4}S_{3}O_{5}Br_{3}$	174	$Li_{3}Al_{0.4}Sc_{1.6}(PO_{4}{)}_{3}$	3.60
$Li_{4}Zr_{2}Si(O_{3}Cl_{2}{)}_{2}$	176	$Li_{0.5}Sr_{0.5}Fe_{0.25}Ta_{0.75}O_{3}$	3.86
$Li_{2}Al_{3}SiS_{2}Br_{5}Cl_{6}$	**−6**	$LiAlSi_{2}O_{6}$	3.79
$Li_{3}ZrBO_{3}Cl_{4}$	136	$(Li_{2}S{)}_{50}(P_{2}S_{5}{)}_{17}(LiBH_{4}{)}_{33}$	4.42
$Li_{3}Al(BrCl_{2}{)}_{2}SiBrCl_{3}$	**26**	$(Li_{2}O{)}_{50}(Al_{2}O_{5}{)}_{5}(P_{2}O_{5}{)}_{45}$	3.78
$Li_{5}AlBr_{3}Cl_{5}$	**28**	$Li_{6}FeCl_{8}$	4.31
$Li_{6}Al_{3}S_{2}O_{5}Cl$	112	$Li_{2}SnS_{3}$	3.25
$Li_{2}AlBSBr_{5}Cl$	76	$(Li_{2}O{)}_{50}(Al_{2}O_{5}{)}_{5}(P_{2}O_{5}{)}_{4}$	4.09
$Li_{2}AlSiS_{2}Cl_{5}$	**29**	$(Li_{2}S{)}_{0.3}((Ga_{2}S_{3}{)}_{0.1}(GeS_{2}{)}_{0.9}{)}_{0.7}$	4.11
$Li_{7}AlBS_{2}Br_{4}Cl_{5}$	56	$Li_{2.88}PO_{3.73}N_{0.14}$	3.99
$Li_{5}Al_{2}S_{4}Cl_{3}$	**21**	$Li_{2}SnS_{3}$	3.48
$Li_{2}Al_{2}S_{3}BrCl$	**23**	$(LiI{)}_{0.3}((Li_{2}S{)}_{0.3}((Ga_{2}S_{3}{)}_{0.1}(GeS_{2}{)}_{0.9}{)}_{0.7}{)}_{0.7}$	4.01
$Li_{6}Al_{2}S_{3}Br_{5}Cl$	57	$Li_{2}SnS_{3}$	3.83
$Li_{4}Zr_{2}B_{3}O_{10}Br$	271	$Li_{0.5}Sr_{0.5}Fe_{0.25}Ta_{0.75}O_{3}$	3.73
$Li_{2}Al_{2}S_{2}OCl_{2}$	89	$(LiI{)}_{0.3}((Li_{2}S{)}_{0.3}((Ga_{2}S_{3}{)}_{0.1}(GeS_{2}{)}_{0.9}{)}_{0.7}{)}_{0.7}$	4.23
$Li_{2}Al_{2}S_{3}Cl_{2}$	**6**	$(LiI{)}_{0.3}((Li_{2}S{)}_{0.3}((Ga_{2}S_{3}{)}_{0.1}(GeS_{2}{)}_{0.9}{)}_{0.7}{)}_{0.7}$	4.12
$Li_{2}Al_{2}S_{2}OBr_{2}$	117	$(LiI{)}_{0.3}((Li_{2}S{)}_{0.3}((Ga_{2}S_{3}{)}_{0.1}(GeS_{2}{)}_{0.9}{)}_{0.7}{)}_{0.7}$	4.01
${\mathrm{Li}}_{4}{\mathrm{ZrAl}}_{4}S_{5}{\left(O_{2}\mathrm{Cl}\right)}_{2}$	155	$(LiI{)}_{0.3}((Li_{2}S{)}_{0.3}((Ga_{2}S_{3}{)}_{0.1}(GeS_{2}{)}_{0.9}{)}_{0.7}{)}_{0.7}$	3.72

Examining the probe structures generated within this work as a whole, it was observed that Comgen generated a variety of compositions with diverse chemistries in terms of their elemental composition and distribution within composition space (Figure 1b, Table 3 and Supporting Information), illustrating how Comgen explores chemical space. The minimum energy structures (probe structures) produced by FUSE after the composition generation are also diverse, in terms of the range of overall structural motifs and local co‐ordination environments, with physically reasonable local co‐ordinations (see Supporting Information). In particular, we highlight two specific results.

**Figure 2:**
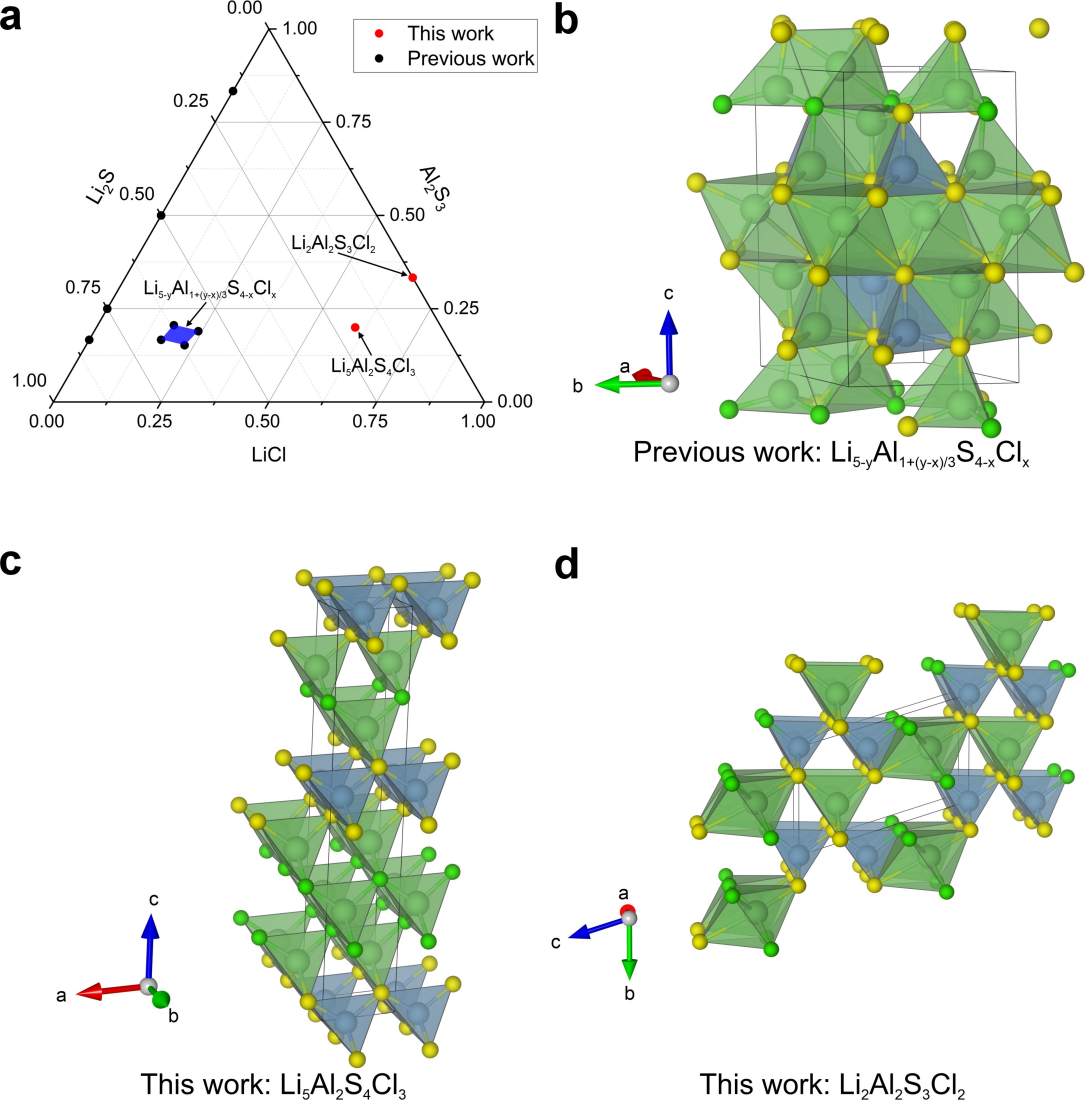
a) Among the compositions studied within this work, two compositions were generated within the previously explored Li−Al−S−Cl phase field.^[34]^ The compositions are presented here as a ternary phase field assembled from LiCl−Li_2_S−${\mathrm{Al}}_{2}S_{3}$ . Four previous compositions lay on the LiCl−${\mathrm{Al}}_{2}S_{3}$ solid solution line (black points), the blue area enclosed by four black points represents the region of composition space where the previously located compound ${\mathrm{Li}}_{5-y}{\mathrm{Al}}_{1+\left(y-x\right)}S_{4-x}{\mathrm{Cl}}_{x}$ . The two red points indicate the two new compositions proposed in this work. b) The representative crystal structure of ${\mathrm{Li}}_{5-y}{\mathrm{Al}}_{1+\left(y-x\right)}S_{4-x}{\mathrm{Cl}}_{x}$ , used in previous simulations^[34]^, with the composition ${\mathrm{Li}}_{13}{\mathrm{Al}}_{3}S_{10}{\mathrm{Cl}}_{2}$ , recalculated as a part of the convex hull calculations for this work and found to be 18 meV atom^−1^ above the convex hull. c) The probe structure at the composition ${\mathrm{Li}}_{5}{\mathrm{Al}}_{2}S_{4}{\mathrm{Cl}}_{2}$ , with an energy computed to be 21 meV atom^−1^ above the previous convex hull, below our target threshold of 45 meV atom^−1^. d) The probe structure of ${\mathrm{Li}}_{2}{\mathrm{Al}}_{2}S_{3}{\mathrm{Cl}}_{2}$ , computed to have an energy above the convex hull of 6 meV atom^−1^, below our target threshold of 45 meV atom^−1^, defining energies of interest to experimental chemistry. Atoms are coloured as follows: Li – light green, Al – cyan, S – yellow and Cl – green.

Firstly, two compositions were generated within the Li−Al−S−Cl phase field, which has been previously explored experimentally,^[34]^ resulting in the discovery of a new compound over the compositional range ${\mathrm{Li}}_{5-y}{\mathrm{Al}}_{1+\left(y-x\right)}S_{4-x}{\mathrm{Cl}}_{x}$ (*x*=0.5‐0.7, y=0.5‐1). With the composition space represented as linear combinations of the starting materials LiCl, Li_2_S and ${\mathrm{Al}}_{2}S_{3}$ , the two compositions selected by Comgen, are separated both from each other, and from previously explored compositions (Figure 2a). The two probe structures generated within this work, ${\mathrm{Li}}_{5}{\mathrm{Al}}_{2}S_{4}{\mathrm{Cl}}_{3}$ and ${\mathrm{Li}}_{2}{\mathrm{Al}}_{2}S_{3}{\mathrm{Cl}}_{2}$ , were both found to have energies relative to the convex hull defining them as experimental targets (+21 and +6 meV atom^−1^, respectively), additionally ${\mathrm{Li}}_{5}{\mathrm{Al}}_{2}S_{4}{\mathrm{Cl}}_{3}$ , is predicted to have a Li ion conductivity greater than 10^−4^ S cm^−1^ at room temperature, highlighting that this composition is likely to be of interest to researchers looking for solid state Li battery electrolytes. The probe structure convex hull energies are both below that of the ordered model of the previously reported compound, which has an energy relative to the convex hull of +26 meV atom^−1^. Both of the generated probe structures are defined by a mixed S and Cl anion lattice, with Li and Al occupying sites that it defines. In the case of ${\mathrm{Li}}_{5}{\mathrm{Al}}_{2}S_{4}{\mathrm{Cl}}_{2}$ , all of the Li and Al atoms occupy tetrahedral sites within the lattice. In ${\mathrm{Li}}_{2}{\mathrm{Al}}_{2}S_{3}{\mathrm{Cl}}_{2}$ , all of the Al occupies tetrahedral sites, with the Li atoms split in a 1 : 1 ratio between occupying distorted octahedral and tetrahedral sites. The structure of ${\mathrm{Li}}_{5}{\mathrm{Al}}_{2}S_{4}{\mathrm{Cl}}_{2}$ is an ordered form of the mineral structure Wurtzite,^[35]^ with ordering of S and Cl and of Li and Al. Order of Li^+^ and Al^3+^ on distinct tetrahedral sites is observed in ${\mathrm{Li}}_{3}{\mathrm{AlS}}_{3}$ ,^[36]^ associated with their different four coordinate ionic radii of 0.59 Å and 0.39 Å respectively. In a synthesised sample, it is likely that the anions would be disordered, as observed in ${\mathrm{Li}}_{4.3}{\mathrm{AlS}}_{3.3}{\mathrm{Cl}}_{0.7}$ ^[34]^ because of the similarity in ionic radii between S^2−^ and Cl^−^, rather than the ordering enforced here by the nature of the periodic density functional theory calculations. Both of the probe structures within the Li−Al−S−Cl phase field, while adopting similar local co‐ordinations to that of the previously known compounds, have overall different structures. The compositions generated within the Li−Al−S−Cl phase field demonstrate the ability of the Comgen‐based workflow to locate the composition(s) of potential new compounds, which are likely to form new structures that are predicted to have a desirable physical property.

**Figure 3:**
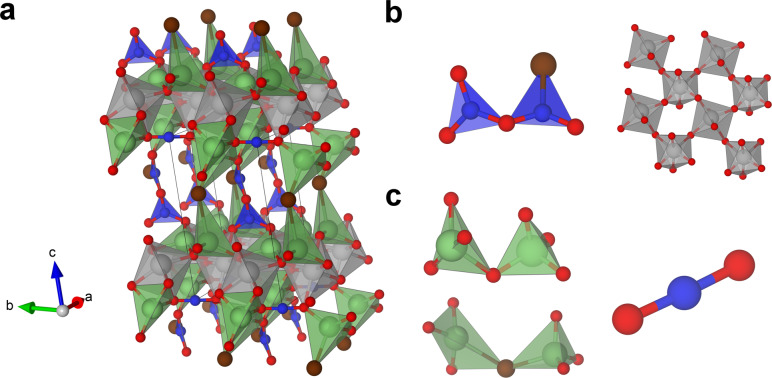
a) The probe structure of ${\mathrm{Li}}_{4}{\mathrm{Zr}}_{2}B_{3}O_{10}\mathrm{Br}$ computed in this work, with an energy relative to the convex hull of +271 meV atom^−1^. b) Two types of environment found within the probe structure that correspond to conventionally found co‐ordinations in ionic compounds, left: two corner sharing trigonal planar B environments and right: a layer of corner sharing ZrO_6_ octahedra. c) left: All of the Li environments within the probe structure are found in heavily distorted tetrahedra. right: one of the B atoms in the structure adopts a linear BO_2_ environment. Atoms are coloured as follows: Li – light green, Zr – grey, B – blue, O – red and Br – Brown.

In contrast to the Li−Al−S−Cl example, ${\mathrm{Li}}_{4}{\mathrm{Zr}}_{2}B_{3}O_{10}\mathrm{Br}$ was computed to have a energy relative to the convex hull of +271 meV atom^−1^, suggesting that the formation of a single phase compound is unlikely at this composition. The computed probe structure is shown in Figure 3, and contains a mixture of conventional co‐ordination environments (Figure 3b) and distorted and atypical environments (Figure 3c). The probe structure is unable to form with reasonable co‐ordinations for all of the cation species, offering an explanation as to why this composition is unlikely to experimentally form a compound. The examples discussed in this section emphasise the advantages of combining all of the tools presented here into one workflow, where Comgen locates points in complex composition spaces that are distant from known compounds, and is then combined with probe structure prediction to explore if stable compounds are likely to form. Examination of the probe structures themselves then provides some explainability behind the predicted stabilities of the generated compositions, and machine learning prediction of properties is used to identify those target compounds of interest as practical Li ion conductors. All of this information can then be used by experimental chemists when prioritising which compositions to explore experimentally, or adjusting the assumptions that generate the constraints used by Comgen. In the workflow used here, structure can be considered early via constraints on ionic radius in Comgen and late with separate CSP tools: reasoning methods can be envisaged that will evaluate structure earlier. The workflow also treats properties with machine learning models applied late on, with the above composition and structure considerations used as proxies to favour properties of interest. The direct integration of quantitative property models into the reasoning‐based Comgen framework appears more challenging than that of structure

## 3 Conclusions

Comgen uses an SMT solver to generate materials compositions satisfying a user‐defined search query, which incorporates criteria such as the distance of candidates from explored chemical space, and relative sizes of potential constituent elements, while respecting provided bonding rules. It is unique within the landscape of support tools for materials science as it makes use of automated reasoning techniques, distinguishing it from previous approaches that enumerate and filter candidate compositions. These automated reasoning techniques provide a fresh perspective on the foundational choices made by synthetic chemists in selecting the chemistry that they explore in the laboratory and thus delimiting the potential realised material outcomes. Distinct from data‐driven techniques, the results provided by Comgen will not be biased by previously discovered materials beyond what is explicitly prompted by the user.

The compositions automatically identified from reasoning by Comgen can be used with other quantitative tools. By combining Comgen with crystal structure prediction to assess thermodynamic stability, we identified as candidates for synthesis eight Li‐containing compositions that satisfy our novelty criteria from a vast composition space comprising 15 elements and are likely to support low‐energy crystal structures. One of these candidates is identified by machine learning as displaying a high Li ion conductivity at room temperature. The workflow driven by Comgen thus transforms design ideas regarding choice of chemistry, extent of novelty and size and bonding control of structure directly into a quantitative assessment of the potential for synthesis of a material matching these criteria.

The constraint sets used are readily expanded to other chemistries and bonding types, and may be used to include assessment of a range of non‐chemical criteria. Our contribution provides quantitative answers to expert user queries, in contrast to brute force surveys of chemical space. It thus defines the consequences of user choices about the chemistry they are interested in, while handling issues that are challenging for a human researcher, such as the high dimensions of multiple element phase fields and the many potential requirements that a candidate target material must meet. The only biases involved are those implicit in the framing of the initial query, and that framing can be revisited by the expert once the quantitative consequences of the original question become clear, for example in the structures and properties that emerge from the lithium ion conductor query above. Further, the answers provided are directly traceable to the constraints used, bringing explainability to the abundantly demonstrated ability of computers to survey chemical space.

## Conflict of Interests

The authors have no conflicts of interest to declare.

4

## Supporting information

As a service to our authors and readers, this journal provides supporting information supplied by the authors. Such materials are peer reviewed and may be re‐organized for online delivery, but are not copy‐edited or typeset. Technical support issues arising from supporting information (other than missing files) should be addressed to the authors.

Supporting Information

## Data Availability

The data that support the findings of this study are available in the supplementary material of this article.

## References

[1] D. W. Davies , K. T. Butler , A. J. Jackson , J. M. Skelton , K. Morita , A. Walsh , J. Open Source Softw. 2019, 4, 1361.

[2] C. J. Hargreaves , M. S. Dyer , M. W. Gaultois , V. A. Kurlin , M. J. Rosseinsky , Chem. Mater. 2020, 32, 10610.

[3] D. W. Davies , K. T. Butler , O. Isayev , A. Walsh , Faraday Discuss. 2018, 211, 553. 30027179 10.1039/c8fd00032h

[4] D. Jha , L. Ward , A. Paul , W.-k. Liao , A. Choudhary , C. Wolverton , A. Agrawal , Sci. Rep. 2018, 8, 17593. 30514926 10.1038/s41598-018-35934-y PMC6279928

[5] R. E. A. Goodall , A. A. Lee , Nat. Commun. 2020, 11, 6280. 33293567 10.1038/s41467-020-19964-7 PMC7722901

[6] A. Wang , S. Kauwe , R. Murdock , T. Sparks , npj Comput. Mater. 2021, 7, 77.

[7] A. Merchant , S. Batzner , S. S. Schoenholz , M. Aykol , G. Cheon , E. D. Cubuk , Nature 2023, 624, 80. 38030720 10.1038/s41586-023-06735-9 PMC10700131

[8] N. Szymanski , B. Rendy , Y. Fei , R. Kumar , T. He , D. Milsted , M. McDermott , M. Gallant , E. Cubuk , A. Merchant , H. Kim , A. Jain , C. Bartel , K. Persson , Y. Zeng , G. Ceder , Nature 2023, 624, 81. 10.1038/s41586-023-06734-w PMC10700133 38030721

[9] A. K. Cheetham , R. Seshadri , Chem. Mater. 2024, 36, 3490. 38681084 10.1021/acs.chemmater.4c00643 PMC11044265

[10] T. Xie , X. Fu , O.-E. Ganea , R. Barzilay , T. Jaakkola , arXiv preprint 2022, 10.48550/arXiv.2110.06197.

[11] R. Jiao , W. Huang , P. Lin , J. Han , P. Chen , Y. Lu , Y. Liu , arXiv preprint 2024, 10.48550/arXiv.2309.04475.

[12] C. Zeni , R. Pinsler , D. Zügner , A. Fowler , M. Horton , X. Fu , S. Shysheya , J. Crabbé , L. Sun , J. Smith , B. Nguyen , H. Schulz , S. Lewis , C.-W. Huang , Z. Lu , Y. Zhou , H. Yang , H. Hao , J. Li , R. Tomioka , T. Xie , arXiv preprint 2024, 10.48550/arXiv.2312.03687.

[13] C. M. Collins , H. Sayeed , G. Darling , J. B. Claridge , T. D. Sparks , M. J. Rosseinsky , Faraday Discuss. 2024, Advance Article, 10.1039/D4FD00094C. 39301819

[14] A. R. Oganov , C. W. Glass , J. Chem. Phys. 2006, 124, 244704. 16821993 10.1063/1.2210932

[15] C. J. Pickard , R. J. Needs , J. Phys. Condens. Matter 2011, 23, 053201. 21406903 10.1088/0953-8984/23/5/053201

[16] C. J. Hargreaves , M. W. Gaultois , L. M. Daniels , E. J. Watts , V. A. Kurlin , M. Moran , Y. Dang , R. Morris , A. Morscher , K. Thompson , M. A. Wright , B.-E. Prasad , F. Blanc , C. M. Collins , C. A. Crawford , B. Duff , J. Evans , J. Gamon , G. Han , B. T. Leube , H. Niu , A. J. Perez , A. Robinson , O. Rogan , P. M. Sharp , E. Shoko , M. Sonni , W. J. Thomas , A. Vasylenko , L. Wang , M. J. Rosseinsky , M. S. Dyer , npj Comput. Mater. 2023, 9, 9..

[17] C. M. Collins , L. M. Daniels , Q. Gibson , M. W. Gaultois , M. Moran , R. Feetham , M. J. Pitcher , M. S. Dyer , C. Delacotte , M. Zanella , C. A. Murray , G. Glodan , O. Pérez , D. Pelloquin , T. D. Manning , J. Alaria , G. R. Darling , J. B. Claridge , M. J. Rosseinsky , Angew. Chem. Int. Ed. 2021, 60, 16457. 10.1002/anie.202102073 PMC8362121 33951284

[18] G. Han , A. Vasylenko , L. M. Daniels , C. M. Collins , L. Corti , R. Chen , H. Niu , T. D. Manning , D. Antypov , M. S. Dyer , J. Lim , M. Zanella , M. Sonni , M. Bahri , H. Jo , Y. Dang , C. M. Robertson , F. Blanc , L. J. Hardwick , N. D. Browning , J. B. Claridge , M. J. Rosseinsky , Science 2024, 383, 739. 38359130 10.1126/science.adh5115

[19] A. Kondori , M. Esmaeilirad , A. M. Harzandi , R. Amine , M. T. Saray , L. Yu , T. Liu , J. Wen , N. Shan , H.-H. Wang , A. T. Ngo , P. C. Redfern , C. S. Johnson , K. Amine , R. Shahbazian-Yassar , L. A. Curtiss , M. Asadi , Science 2023, 379, 499. 36730408 10.1126/science.abq1347

[20] B. Zhang , J. Wang , T. Zou , S. Zhang , X. Yaer , N. Ding , C. Liu , L. Miao , Y. Li , Y. Wu , J. Mater. Chem. C 2015, 3, 11406.

[21] N. S. Bjørner, C. Eisenhofer, L. Kovács, Satisfiability Modulo Custom Theories in Z3, in *Verification, Model Checking, and Abstract Interpretation – 24th International Conference, VMCAI 2023, Boston, MA, USA, January 16–17, 2023, Proceedings*, Verification, Model Checking, and Abstract Interpretation, Springer **2023** pages 91–105.

[22] T. Balyo, M. Heule, M. Iser, M. Järvisalo, M. Suda (Editors), *Proceedings of SAT Competition 2023: Solver, Benchmark and Proof Checker Descriptions*, Department of Computer Science Series of Publications B, Department of Computer Science, University of Helsinki, Finland **2023**.

[23] A. Biere, M. Heule, H. van Maaren, T. Walsh (Editors), *Handbook of Satisfiability – Second Edition*, vol. 336 of *Frontiers in Artificial Intelligence and Applications*, IOS Press **2021**.

[24] D. Pettifor , Solid State Commun. 1984, 51, 31.

[25] A. M. Abakumov , S. S. Fedotov , E. V. Antipov , J.-M. Tarascon , Nat. Commun. 2020, 11, 4976. 33009387 10.1038/s41467-020-18736-7 PMC7532470

[26] M. York , K. Larson , K. C. Harris , E. Carmona , P. Albertus , R. Sharma , M. Noked , E. Strauss , H. Ragones , D. Golodnitsky , J. Solid State Electrochem. 2022, 26, 1851.

[27] R. Li , R. Deng , Z. Wang , Y. Wang , G. Huang , J. Wang , F. Pan , J. Solid State Electrochem. 2023, 27, 1291.

[28] Y. Pang , Y. Zhu , F. Fang , D. Sun , S. Zheng , J. Mater. Sci. Technol. 2023, 161, 136.

[29] C. Collins , G. R. Darling , M. J. Rosseinsky , Faraday Discuss. 2018, 211, 117. 30033457 10.1039/c8fd00045j

[30] P. Adeli , J. D. Bazak , K. H. Park , I. Kochetkov , A. Huq , G. R. Goward , L. F. Nazar , Angew. Chem. Int. Ed. 2019, 58, 8681. 10.1002/anie.201814222 31041839

[31] A. Bubenzer , R. Nitsche , E. Grieshaber , Acta Crystallogr. Sect. B 1976, 32, 2825.

[32] C. Collins , M. Dyer , M. Pitcher , G. Whitehead , M. Zanella , P. Mandal , J. Claridge , G. Darling , M. Rosseinsky , Nature 2017, 546, 280–284. 28593963 10.1038/nature22374

[33] A. M. Ganose , A. Jain , MRS Commun. 2019, 9, 874.

[34] J. Gamon , M. S. Dyer , B. B. Duff , A. Vasylenko , L. M. Daniels , M. Zanella , M. W. Gaultois , F. Blanc , J. B. Claridge , M. J. Rosseinsky , Chem. Mater. 2021, 33, 8733. 34840424 10.1021/acs.chemmater.1c02751 PMC8613839

[35] E. H. Kisi , M. M. Elcombe , Acta Crystallogr. Sect. C 1989, 45, 1867.

[36] J. Gamon , B. B. Duff , M. S. Dyer , C. Collins , L. M. Daniels , T. W. Surta , P. M. Sharp , M. W. Gaultois , F. Blanc , J. B. Claridge , M. J. Rosseinsky , Chem. Mater. 2019, 31, 9699. 32063680 10.1021/acs.chemmater.9b03230 PMC7011735

