# Supplementary material for: Exploration of Chemical Space Through Automated Reasoning
Source: Angew Chem Int Ed Engl. 2025 Jan 5;64(6):e202417657. doi: 10.1002/anie.202417657 (PMC11795740; doi:10.1002/anie.202417657)
Supplement: Supplementary file 1 — Supporting Information [file ANIE-64-e202417657-s001.pdf]

Angewandte Chemie
Eine Zeitschrift der Gesellschaft Deutscher Chemiker

Supporting Information

Exploration of Chemical Space Through Automated Reasoning

J. Clymo, C. M. Collins, K. Atkinson, M. S. Dyer, M. W. Gaultois, V. V. Gusev, M. J. Rosseinsky, S. Schewe*

Exploration of Chemical Space through Automated Reasoning - Supplementary Information

Judith Clymo[1], Christopher M. Collins[2,3], Katie Atkinson[1,3], Matthew S. Dyer[2,3], Michael W. Gaultois[2,3], Vladimir V. Gusev[1,3], Matthew J. Rosseinsky*[2,3], and Sven Schewe[1]

[1]Department of Computer Science, University of Liverpool, Ashton Building, Ashton Street, Liverpool L69 3BX, United Kingdom
[2]Department of Chemistry, University of Liverpool, Crown Street, Liverpool L69 7ZD, United Kingdom
[3]Leverhulme Research Centre for Functional Materials Design, Materials Innovation Factory, Liverpool L7 3NY, United Kingdom

1 Computational Methods

1.1 Mathematical background

We model user requirements on the compositions as logical constraints over the space of all possible combinations of elements. These constraints are automatically generated based on input from the user. All included constraints can be modelled in the language of linear arithmetic over integers and / or reals (LIRA, Linear Integer Real Arithmetic). Constraints are combined using Boolean operators. Overall this gives rise to a Boolean satisfiability problem modulo LIRA, solved by a Satisfiability Modulo Theory (SMT) solver – we use the SMT solver Z3 [1]. We now give a brief review of Boolean satisfiability, LIRA, and SMT.

Boolean satisfiability Boolean formulas consist of variables that can take values 0 and 1 (for *false* and *true*); operators $\wedge$, $\vee$, and $\neg$ (for *and*, *or*, and *negation*, respectively); and parentheses. Two example formulas would be $\Phi = (x \vee y) \wedge (\neg x \vee \neg y)$ and $\Psi = (x \vee y) \wedge \neg x \wedge \neg y$. The Boolean satisfiability (SAT) problem asks, for a given Boolean formula, whether or not there is a satisfying assignment. For example, Φ has two satisfying assignments: $x = 1, y = 0$ and $x = 0, y = 1$; Φ is therefore *satisfiable*. Ψ has no satisfying assignment and is therefore *unsatisfiable*.

To motivate how Boolean logic might be useful in finding possible compositions, consider the following example. To define which compositions to generate we may wish to restrict the choice of elements. Let the Boolean variable x_e mean "the composition contains a non-zero quantity of element e". Then we can express the requirement for a composition to contain an element from group one and an element from group seven as

$$(x_{Li} \vee x_{Na} \vee x_K \vee x_{Rb}) \wedge (x_F \vee x_{Cl} \vee x_{Br} \vee x_I),$$

i.e. "the composition contains a non-zero quantity of lithium *or* the composition contains a non-zero quantity of sodium *or* ... *and* the composition contains a non-zero quantity of fluorine *or* ...".

Solving Boolean formulas A naïve way of solving the satisfiability problem of a Boolean formula is to try all different combinations of valuations of the variables. However, even if we only have 40 variables, the number of different valuations is a 13 digit number (trillions of different valuations), which rises to a 31 digit number for 100 variables (nonillions of different valuations). Fully enumerating

all possible assignments to the variables is usually infeasible – just as fully enumerating all possible compositions is infeasible.

Despite their conceptual simplicity, deciding whether an arbitrary Boolean formula is true or false is NP-complete [2]. It is a major open problem in computer science as to whether there exists an efficient (polynomial running time) algorithm to solve any NP-complete problem. This means that we expect any algorithm for solving arbitrary SAT problems will, on some instances, have running time similar to that of enumerating all possible assignments. Certainly all existing algorithms to solve the SAT problem have exponential worst-case running time. Despite this somewhat negative forecast, improving algorithms and heuristics for SAT solvers is a highly active research area in computer science, with modern solvers often able to solve real-world instances containing thousands or even millions of variables.

A crucial part of many modern SAT solving algorithms is the ability to derive new constraints based on the initial system [3]. When an assignment is found to not satisfy the constraints, the algorithm analyses the reason for this failure and abstracts the reason as an additional constraint. The new constraint now acts as a summary of assignments that can be ignored since they would also violate the initial constraints for the same reason as the already tested assignment. In addition, carefully tuned heuristics are used to determine the order in which to test assignments so that either a satisfying assignment is found quickly or the derived clauses are maximally helpful in guiding the rest of the search. Further heuristics continually analyse the set of additional constraints to balance the benefit they provide against memory efficiency requirements. These heuristic and algorithmic innovations are the reason that SAT solving algorithms can regularly outperform brute force approaches by orders of magnitude.

Satisfiability modulo theories Pure Boolean logic is restrictive and some concepts are either impossible or unnatural to represent in this framework. Satisfiability modulo theories (SMT) are a generalisation of SAT. 'Theories' here refers to a well-defined way of expressing logical or mathematical concepts. The chosen theories are combined with Boolean reasoning so that SMT solvers can (for example) solve formulas involving arithmetic combinations of real numbers or integers, reason about functions, and interpret data structures like arrays. An SMT problem is a set of statements in some theory, which are combined using Boolean connectives. An SMT solver shares the work of deciding such a formula between a SAT solver, which reasons about the overall Boolean formula, and a specialised theory solver, which reasons about the components of the formula.

Linear arithmetic The theory of linear (integer) arithmetic reasons about equations and inequalities over real (integer) valued variables. Each such statement has the form $\Sigma_{i=0}^{n} c_i x_i \bowtie C$, where c_i and C are constants and x_i are (real or integer) variables. The symbol $\bowtie$ stands for one of $\{\geq, >, \leq, <, =, \neq\}$. A system of linear inequalities is a conjunction of two or more linear inequalities.

When all variables are real-valued, linear arithmetic problems can be solved in polynomial time in the number of variables. However, when some or all variables are required to take integer values, deciding whether a system of linear arithmetic constraints is satisfiable is NP-complete.

There are several popular algorithms for solving linear arithmetic problems, including the Simplex algorithm for problems with real-valued variables, and cutting planes and branch and bound methods where variables must have integer values.

The following example demonstrates how a SAT solver and linear arithmetic solver can work together to solve a composite problem. For simplicity, this example is abstract and has no relation to chemistry. In the following section we describe the implementation of constraints on compositions. Although some of the composition constraints are more complex, they remain expressible as collections of linear arithmetic constraints, and are handled by the constraint solver in a similar way to the example shown here.

Example 1. *Let* $A := (x+y=3)$, $B := (3z-2x=0)$, $C := (x-3y=2)$, $D := (y>x)$, $E := (x>0)$. *Consider the formula* $A \wedge (B \vee C) \wedge D \wedge E$. *An SMT solver first seeks a satisfying assignment to the abstracted SAT problem. For example, assigning* A, C, D *and* E *to true,* B *to false satisfies the formula. Next, a theory solver checks the implied system of linear (in)equalities, in this case*

$$\begin{aligned} x+y &= 3 \\ 3z-2x &\neq 0 \\ x-3y &= 2 \\ y &> x \\ x &> 0 \end{aligned}$$

This system is not satisfiable, because $y > x > 0$ *implies* $x - 3y < 0$ *which contradicts* $x - 3y = 2$. *The SAT solver generates a new assignment for the theory solver to check. For example, assigning* A, B, D *and* E *to true,* C *to false satisfies the formula and generates the following problem for the linear arithmetic solver to check:*

$$\begin{aligned} x+y &= 3 \\ 3z-2x &= 0 \\ x-3y &\neq 2 \\ y &> x \\ x &> 0 \end{aligned}$$

This system is satisfiable with $x = 1$, $y = 2$ *and* $z = \frac{2}{3}$. *Once the theory solver confirms this, the algorithm terminates. Note that the formula is not satisfiable if* x, y *and* z *are required to be integers.*

1.2 Mathematical description of Comgen constraints

We now formally describe the available types of constraints. The definitions are given in a slightly simplified form and are further manipulated within the tool to suit the formatting requirements of the underlying SMT solver. This does not significantly change the formulation but would make constraints more difficult for a human to understand.

Element selection The constants n_{sp-el} indicate the quantity of element el in species sp. This quantity is either 0 or 1 except in the case of poly-atomic species. For example, consider the poly-atomic species SO_4. The constant n_{SO_4-O} has value 4 and n_{SO_4-S} has value 1. For all other elements, n_{SO_4-el} is 0. Variables Q_{el} and R_{sp} represent the quantity of element el and of species sp, respectively. The quantity of element el in a composition is given by

$$Q_{el} = \sum_{sp} (R_{sp} \cdot n_{sp-el}).$$

All quantities are non-negative, and the total quantity of all elements must sum to 1.

$$R_{sp} >= 0,$$

$$\sum_{el} Q_{el} = 1.$$

The user can optionally set lower and upper bounds (l_{inc} and u_{inc}) on the number of distinct elements that are included from a subset E_1 of the permitted elements. A Boolean variable, δ_{el}, is introduced. When set to true (1) by the solver, δ_{el} indicates that the quantity of element el is not zero. Taking

the sum over the δ variables (casting true to 1 and false to 0) then counts the number of elements used in the composition.

$$l_{inc} \leq \sum_{el \in E_1} \delta_{el} \leq u_{inc},$$
$$\delta_{el} = 0 \vee Q_{el} \neq 0,$$
$$\delta_{el} = 1 \vee Q_{el} = 0,$$

Similarly, lower and upper bounds (l_{quant} and u_{quant}) may be specified for the quantity of individual elements or across a subset E_2 of the elements.

$$l_{quant} \leq \sum_{el \in E_2} Q_{el} \leq u_{quant}.$$

Charge balancing The constant c_{sp} records the charge of species sp. As before, the variable R_{sp} denotes the quantity of species sp in the composition. The requirement that the total charge of the composition must be zero is encoded by

$$\sum_{sp} R_{sp} \cdot c_{sp} = 0.$$

The constant x_{el} is the electronegativity of element el; the Boolean variable P_{el} indicates whether a positively charged species of element el is included in the composition (has a non-zero quantity) and Boolean variable N_{el} indicates the presence of a negatively charges species of element el. Poly-atomic species are ignored. To enforce that selected charges respect the electronegativity of the elements we forbid the inclusion of both a positively charged species for el_1 and a negatively charged species for el_2 whenever el_1 has the greater electronegativity.

$$(x_{el_1} \geq x_{el_2}) \vee P_{el_1} = 0 \vee N_{el_2} = 0.$$

Distance from known materials To calculate the ElMD between two compositions we fix an ordering over constituent elements and compare the resulting vector representations. The ordering of elements is based on the Pettifor scale.

Auxiliary variables D_i and A_i are introduced to store intermediate values in the calculation, and variable T is the total distance (the ElMD) between the generated composition and the comparison composition. Constants q_i specify the element quantities (which have been normalised) of the comparison composition. As before, variables Q_{el} denote the quantity of element el in the generated composition, although we now use the subscript i to emphasise that here the ordering of elements is important instead of their chemical symbol.

To calculate the ElMD, we first find the difference between the values at each position in the composition arrays (below, this is $Q_i - q_i$). Add this to the value calculated for the previous position (D_i) to get the value for this position (D_{i+1}). Finally, sum the absolute value at each position (the absolute value is A_i, defined to be equal to $|D_i|$).

Constants l_{dist} and u_{dist} are user-specified bounds on the distance.

$$D_0 = 0,$$
$$A_i \geq 0,$$
$$D_{i+1} = Q_i + D_i - q_i,$$
$$(A_i = D_i) \vee (A_i = -D_i),$$
$$T = \sum_i A_i,$$
$$l_{dist} \leq T \leq u_{dist}.$$

Integer stoichiometry The user specifies lower and upper bounds on the total of the integer stoichiometric coefficients, denoted by l_{sto} and u_{sto}. The solver chooses a value N from within the user specified range.

$$l_{sto} \leq \ N \leq u_{sto}.$$

Now the solver is required to set the quantity of each species to a fraction having the total as denominator For example, if N is selected as 5 then the possible quantities for each species is taken from the set $\{0, \frac{1}{5}, \frac{2}{5}, \frac{3}{5}, \frac{4}{5}, 1\}$. Quantities for each species are selected to ensure that the stoichiometries can be given as integers having a total quantity N within the specified range. Because N was not known when the constraints were generated, but is rather chosen as part of the solving process, the constraint system is initially set up allowing for any possible choice of N. Constraints are included so that once N is selected, the possible species quantities are restricted accordingly. As before, the variable R_{sp} denotes the quantity of species sp in the composition. The following constraint is added for each species that could be used in the composition.

$$\forall n \in \{l_{sto}, \ldots, u_{sto}\} \ n \neq N \vee \bigvee_{i \in \{0, \ldots, n\}} R_{sp} = \frac{i}{n}.$$

Starting materials The constants q^i_{el} represent the quantity of element el in the user-provided starting material i. We require for each element el that

$$\sum_i M_i \cdot q^i_{el} = Q_{el},$$

where M_i are the variables representing the amount of starting material i to be used in a potential synthesis, and Q_{el} is the quantity of element el.

Optionally, the user may also specify other metrics, such as costs or elemental scarcity, for each starting material and search for compositions formed from these with a bound on the total cost or other metric selected. The user may specify costs c_i for each material, as well as the maximum total cost t. We then bound the total cost of the materials selected by requiring that

$$\sum_i c_i \cdot M_i \leq t.$$

Comparing properties of ions Given sets of species s_i and s_j with radii r_i and r_j, bounds u_{ratio} and l_{ratio} on the ratio of ion sizes, plus Boolean variables $P_{i,j}$ representing the selection of pair (i, j) and real variables R_{sp} representing the quantity of species sp in the composition, we require that

$$\begin{aligned} &\neg P_{i,j} \vee (R_i > 0 \wedge R_j > 0), \\ &\bigvee_{i,j} P_{i,j}, \\ &\neg P_{i,j} \vee (r_i/r_j \leq u_{ratio}), \\ &\neg P_{i,j} \vee (r_i/r_j \geq l_{ratio}). \end{aligned}$$

When constraining the absolute different of ionic radii, we require that all pairs of species selected have a radius difference within given bounds. Reusing the notation above, with u_{diff} and l_{diff} representing upper and lower bounds respectively on the absolute difference, we require that

$$\begin{aligned} &P_{i,j} \vee \neg(R_i > 0 \wedge R_j > 0), \\ &\neg P_{i,j} \vee (|r_i - r_j| \leq u_{diff}), \\ &\neg P_{i,j} \vee (|r_i - r_j| \geq l_{diff}). \end{aligned}$$

1.3 Python Interface

Comgen is designed for simplicity and ease of use. We provide a high-level Python interface through which the user specifies which constraints are required and provide relevant parameters. The logical constraints are automatically generated and combined before being handed to the constraint solver.

Most of the constraints belong to the `TargetComposition` class for general compositions and the `IonicComposition` class for ionic materials. The user simply creates a `TargetComposition` object and calls the relevant constraint. For example, fixing that the desired composition should have between 3 and 6 elements and exactly 15 atoms is expressed by the following two lines of code.

```
target_composition.distinct_elements(lb=3, ub=6)
target_composition.total_atoms(15)
```

Detailed examples, including code to reproduce the queries given in our case studies, are available at `https://github.com/jclymo/comgen`. Comgen is also available as a Python package at `https://pypi.org/project/comgen`.

1.4 Computational setup

Once compositions are generated by Comgen, they are passed to FUSE for crystal structure prediction. There are two options for how to run FUSE for each of the generated compositions (Figure **??**): Option 1 - Copy all of the generated files across to a high-performance computing facility, such that all of the compositions can be computed simultaneously or Option 2 - Sequentially run all of the FUSE calculations on the local machine. We use Option 1 in our experiments.

FUSE generates an initial pool of crystal structures by running a generative machine learning (ML) model, these structures are then ranked by their energy, with the best structures selected as the starting population for a Basin Hopping (BH) search routine. For the BH search, the generated structures are broken down into small units referred to as sub-modules, where new candidate structures are generated by three classes of action type: i) modifying the atomic structure of sub-modules, ii) re-arranging the order in which the sub-modules appear in the structure or iii) generating new crystal structures, which are in turn broken down into sub-modules.

For runs of FUSE presented in this work, the initial population consisted of the top 25 structures generated by the ML model used by FUSE, then the basin hopping routine was run until 200 structures had been generated since the global minimum was located. The ML pool of structures was assembled by running the ML generator for 25,000 structures, which were then locally optimised and ranked using the ChgNET machine learnt inter-atomic potential [4]. Each FUSE run was limited to crystal structures with a maximum size of 20 atoms per unit cell to match the maximum number of atoms used in the search query (see Results and Discussion). For each generated structure, the energy calculation was performed in two stages: Firstly to tidy the geometry prior to performing density functional theory calculations, the structure was initially optimised using ChgNET, for up to 2,000 cycles (the total run time for this stage is approximately 1-2 minutes), or until the forces are below 0.075 $eVÅ^{-1}$. Secondly, the structure was optimised with the density functional theory code VASP [5] with PBE pseudo-potentials [6], for a total of 160 steps or until the forces are below 0.03 $eVÅ^{-1}$, Γ-centred k-point grids are generated using the "KSPACING" setting in VASP, with the value at the final step of the calculation of 0.3. Once the FUSE calculation had converged, the lowest energy structure was then re-optimised in VASP using the SCAN metaGGA functional [7, 8] and a plane wave cutoff energy of 650 eV, with the remaining VASP settings unchanged. Once a candidate crystal structure has been predicted for a composition, energies relative to the convex hull are then computed using pymatgen [9], using 782 ordered reference structures from 263 different compounds containing combinations of the elements used within the search and fewer than the upper limit of six elements from the ICSD database [10], the materials project [11] and previous work [12] computed as described above for the final probe structures, for a full list of compounds, see the csv datatable provided as part of the supporting information.

All of the FUSE and VASP calculations in this work were then run on the Tier 2 computing facility Cirrus (see acknowledgements), with each FUSE job running on one 36 core CPU node.

1.5 Visualization and written descriptions of crystal structures

All crystal structure illustrations were prepared using VESTA [13]. All of the probe structures presented in the Supplementary Information are provided with a description that has been automatically generated using the language model Robocrystallographer [14], using the prompt: "robocrys <input_structure.cif> −−conventional −−symmetry −−symprec 0.001". Descriptions were then edited to remove grammatical errors, these descriptions are included to help illustrate the diversity of chemistry sampled by Comgen in this work.

2 Probe structures from Automated Workflow

Below we provide images of the final probe structures for all of the compositions referenced in the main text.

Generated descriptions have then been edited for clarity and to correct typographical errors. All structural images were generated using VESTA[13], where generated structures are flat in shape, the "Standardisation of crystal data" function within VESTA was used to reshape the unit cell for these figures for clarity, with figures exported from VESTA, the default colour pallette was used for each of the figures in this section. The structure descriptions were generated using robocrystallographer [14] as outlined in the methods section.

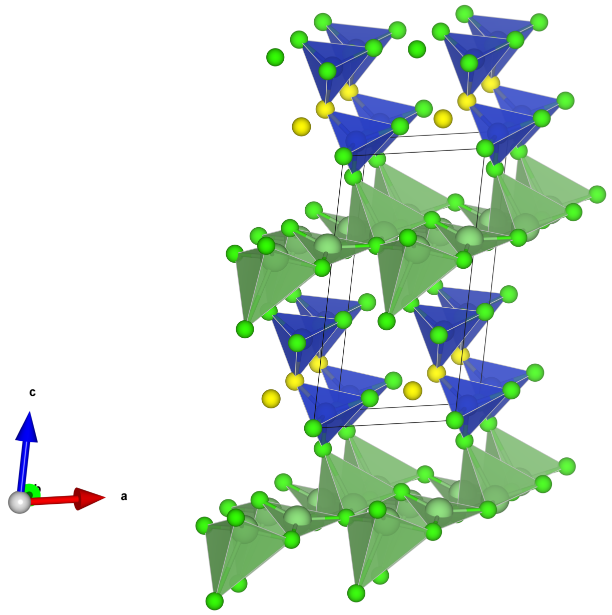

Figure S1: The predicted structure of $Li_3Si_2SCl_9$.

1. **$Li_3Si_2SCl_9$** crystallizes in the triclinic P1 space group. The structure is two-dimensional and consists of one $Li_3Si_2SCl_9$ sheet oriented in the (0, 0, 1) direction. There are three inequivalent Li^{1+} sites. In the first Li^{1+} site, $Li(1)^{1+}$ is bonded in a trigonal planar geometry to one $Cl(2)^{1-}$, one $Cl(3)^{1-}$, and one $Cl(5)^{1-}$ atom. The Li(1)-Cl(2) bond length is 2.26 Å. The Li(1)-Cl(3) bond length is 2.27 Å. The Li(1)-Cl(5) bond length is 2.26 Å. In the second Li^{1+} site, $Li(2)^{1+}$ is bonded in a trigonal planar geometry to one $Cl(2)^{1-}$, one $Cl(3)^{1-}$, and one $Cl(5)^{1-}$ atom. The Li(2)-Cl(2) bond length is 2.28 Å. The Li(2)-Cl(3) bond length is 2.27 Å. The Li(2)-Cl(5) bond length is 2.26 Å. In the third Li^{1+} site, $Li(3)^{1+}$ is bonded to one $Cl(2)^{1-}$, one $Cl(3)^{1-}$, one

$Cl(4)^{1-}$, and one $Cl(5)^{1-}$ atom to form distorted $LiCl_4$ trigonal pyramids that share a corner with one $Si(1)SCl_3$ tetrahedra. The Li(3)-Cl(2) bond length is 2.29 Å. The Li(3)-Cl(3) bond length is 2.28 Å. The Li(3)-Cl(4) bond length is 2.89 Å. The Li(3)-Cl(5) bond length is 2.27 Å. There are two inequivalent Si^{4+} sites. In the first Si^{4+} site, $Si(1)^{4+}$ is bonded to one $S(1)^{2-}$, one $Cl(1)^{1-}$, one $Cl(4)^{1-}$, and one $Cl(8)^{1-}$ atom to form $SiSCl_3$ tetrahedra that share a corner with one $Si(2)SCl_3$ tetrahedra and a corner with one $Li(3)Cl_4$ trigonal pyramid. The Si(1)-S(1) bond length is 2.12 Å. The Si(1)-Cl(1) bond length is 2.02 Å. The Si(1)-Cl(4) bond length is 2.03 Å. The Si(1)-Cl(8) bond length is 2.02 Å. In the second Si^{4+} site, $Si(2)^{4+}$ is bonded to one $S(1)^{2-}$, one $Cl(6)^{1-}$, one $Cl(7)^{1-}$, and one $Cl(9)^{1-}$ atom to form corner-sharing $SiSCl_3$ tetrahedra. The Si(2)-S(1) bond length is 2.12 Å. The Si(2)-Cl(6) bond length is 2.02 Å. The Si(2)-Cl(7) bond length is 2.02 Å. The Si(2)-Cl(9) bond length is 2.03 Å. $S(1)^{2-}$ is bonded in a water-like geometry to one $Si(1)^{4+}$ and one $Si(2)^{4+}$ atom. There are nine inequivalent Cl^{1-} sites. In the first Cl^{1-} site, $Cl(1)^{1-}$ is bonded in a single-bond geometry to one $Si(1)^{4+}$ atom. In the second Cl^{1-} site, $Cl(2)^{1-}$ is bonded in a trigonal non-coplanar geometry to one $Li(1)^{1+}$, one $Li(2)^{1+}$, and one $Li(3)^{1+}$ atom. In the third Cl^{1-} site, $Cl(3)^{1-}$ is bonded in a trigonal non-coplanar geometry to one $Li(1)^{1+}$, one $Li(2)^{1+}$, and one $Li(3)^{1+}$ atom. In the fourth Cl^{1-} site, $Cl(4)^{1-}$ is bonded in a distorted bent 120 degrees geometry to one $Li(3)^{1+}$ and one $Si(1)^{4+}$ atom. In the fifth Cl^{1-} site, $Cl(5)^{1-}$ is bonded in a trigonal planar geometry to one $Li(1)^{1+}$, one $Li(2)^{1+}$, and one $Li(3)^{1+}$ atom. In the sixth Cl^{1-} site, $Cl(6)^{1-}$ is bonded in a single-bond geometry to one $Si(2)^{4+}$ atom. In the seventh Cl^{1-} site, $Cl(7)^{1-}$ is bonded in a single-bond geometry to one $Si(2)^{4+}$ atom. In the eighth Cl^{1-} site, $Cl(8)^{1-}$ is bonded in a single-bond geometry to one $Si(1)^{4+}$ atom. In the ninth Cl^{1-} site, $Cl(9)^{1-}$ is bonded in a single-bond geometry to one $Si(2)^{4+}$ atom.

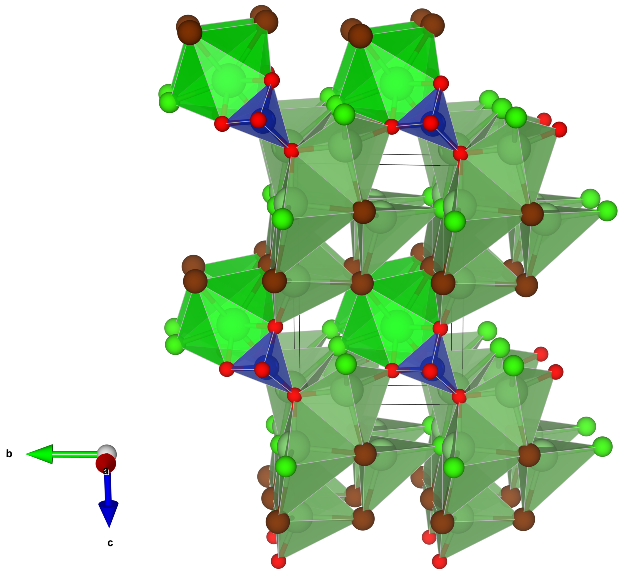

Figure S2: The predicted structure of $Li_5ZrSiBr_3(O_2Cl)_2$.

2. $\mathbf{Li_5ZrSiBr_3(O_2Cl)_2}$ is Chalcostibite-derived structured and crystallizes in the triclinic P1 space group. There are five inequivalent Li^{1+} sites. In the first Li^{1+} site, $Li(1)^{1+}$ is bonded in a 5-coordinate geometry to one $O(1)^{2-}$, one $Br(1)^{1-}$, one $Br(3)^{1-}$, and two equivalent $Cl(1)^{1-}$ atoms. The Li(1)-O(1) bond length is 1.93 AA. The Li(1)-Br(1) bond length is 2.67 AA. The Li(1)-Br(3) bond length is 2.91 AA. There is one shorter (2.49 AA) and one longer (2.63 AA) Li(1)-Cl(1) bond length. In the second Li^{1+} site, $Li(2)^{1+}$ is bonded in a 4-coordinate geometry to one $O(1)^{2-}$, one $O(3)^{2-}$, one $Cl(1)^{1-}$, and one $Cl(2)^{1-}$ atom. The Li(2)-O(1) bond length is 1.97 AA. The Li(2)-O(3) bond length is 2.03 AA. The Li(2)-Cl(1) bond length is 2.34 AA. The Li(2)-Cl(2) bond length is 2.43 AA. In the third Li^{1+} site, $Li(3)^{1+}$ is bonded to one $Br(2)^{1-}$,

two equivalent $Br(1)^{1-}$, and one $Cl(1)^{1-}$ atom to form corner-sharing $LiBr_3Cl$ tetrahedra. The Li(3)-Br(2) bond length is 2.60 AA. There is one shorter (2.65 AA) and one longer (2.68 AA) Li(3)-Br(1) bond length. The Li(3)-Cl(1) bond length is 2.38 AA. In the fourth Li^{1+} site, $Li(4)^{1+}$ is bonded in a 5-coordinate geometry to one $O(2)^{2-}$, one $Br(2)^{1-}$, two equivalent $Br(3)^{1-}$, and one $Cl(1)^{1-}$ atom. The Li(4)-O(2) bond length is 1.88 AA. The Li(4)-Br(2) bond length is 2.53 AA. There is one shorter (2.64 AA) and one longer (2.66 AA) Li(4)-Br(3) bond length. The Li(4)-Cl(1) bond length is 2.86 AA. In the fifth Li^{1+} site, $Li(5)^{1+}$ is bonded in a 4-coordinate geometry to one $O(1)^{2-}$, one $O(4)^{2-}$, one $Br(1)^{1-}$, and one $Cl(2)^{1-}$ atom. The Li(5)-O(1) bond length is 1.97 AA. The Li(5)-O(4) bond length is 1.92 AA. The Li(5)-Br(1) bond length is 2.52 AA. The Li(5)-Cl(2) bond length is 2.49 AA. $Zr(1)^{4+}$ is bonded in a 6-coordinate geometry to one $O(2)^{2-}$, one $O(3)^{2-}$, one $O(4)^{2-}$, one $Br(2)^{1-}$, one $Br(3)^{1-}$, and one $Cl(2)^{1-}$ atom. The Zr(1)-O(2) bond length is 2.05 AA. The Zr(1)-O(3) bond length is 2.01 AA. The Zr(1)-O(4) bond length is 2.03 AA. The Zr(1)-Br(2) bond length is 2.73 AA. The Zr(1)-Br(3) bond length is 2.73 AA. The Zr(1)-Cl(2) bond length is 2.54 AA. $Si(1)^{4+}$ is bonded in a tetrahedral geometry to one $O(1)^{2-}$, one $O(2)^{2-}$, one $O(3)^{2-}$, and one $O(4)^{2-}$ atom. The Si(1)-O(1) bond length is 1.60 AA. The Si(1)-O(2) bond length is 1.65 AA. The Si(1)-O(3) bond length is 1.62 AA. The Si(1)-O(4) bond length is 1.66 AA. There are four inequivalent O^{2-} sites. In the first O^{2-} site, $O(1)^{2-}$ is bonded in a distorted trigonal pyramidal geometry to one $Li(1)^{1+}$, one $Li(2)^{1+}$, one $Li(5)^{1+}$, and one $Si(1)^{4+}$ atom. In the second O^{2-} site, $O(2)^{2-}$ is bonded in a distorted trigonal non-coplanar geometry to one $Li(4)^{1+}$, one $Zr(1)^{4+}$, and one $Si(1)^{4+}$ atom. In the third O^{2-} site, $O(3)^{2-}$ is bonded in a distorted trigonal planar geometry to one $Li(2)^{1+}$, one $Zr(1)^{4+}$, and one $Si(1)^{4+}$ atom. In the fourth O^{2-} site, $O(4)^{2-}$ is bonded in a distorted trigonal planar geometry to one $Li(5)^{1+}$, one $Zr(1)^{4+}$, and one $Si(1)^{4+}$ atom. There are three inequivalent Br^{1-} sites. In the first Br^{1-} site, $Br(1)^{1-}$ is bonded in a 4-coordinate geometry to one $Li(1)^{1+}$, one $Li(5)^{1+}$, and two equivalent $Li(3)^{1+}$ atoms. In the second Br^{1-} site, $Br(2)^{1-}$ is bonded in a distorted trigonal planar geometry to one $Li(3)^{1+}$, one $Li(4)^{1+}$, and one $Zr(1)^{4+}$ atom. In the third Br^{1-} site, $Br(3)^{1-}$ is bonded in a distorted see-saw-like geometry to one $Li(1)^{1+}$, two equivalent $Li(4)^{1+}$, and one $Zr(1)^{4+}$ atom. There are two inequivalent Cl^{1-} sites. In the first Cl^{1-} site, $Cl(1)^{1-}$ is bonded in a distorted square pyramidal geometry to one $Li(2)^{1+}$, one $Li(3)^{1+}$, one $Li(4)^{1+}$, and two equivalent $Li(1)^{1+}$ atoms. In the second Cl^{1-} site, $Cl(2)^{1-}$ is bonded in a 3-coordinate geometry to one $Li(2)^{1+}$, one $Li(5)^{1+}$, and one $Zr(1)^{4+}$ atom.

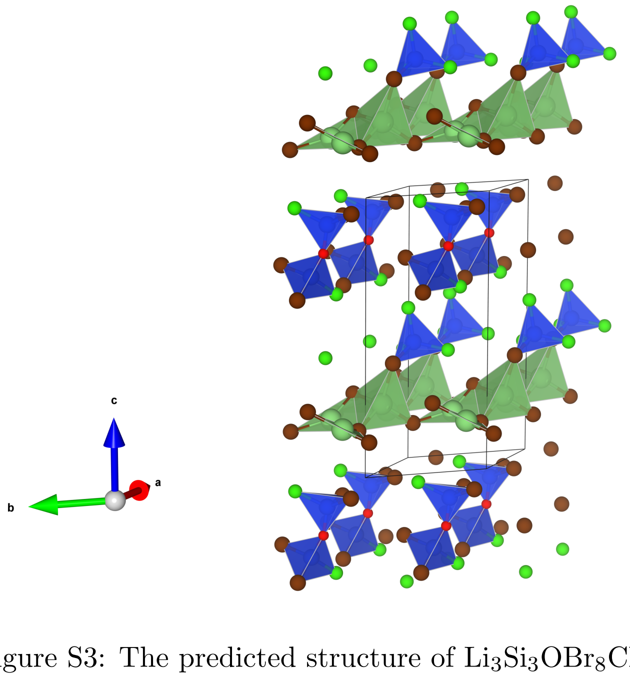

Figure S3: The predicted structure of $Li_3Si_3OBr_8Cl_5$.

3. **$Li_3Si_3OBr_8Cl_5$** crystallizes in the triclinic P1 space group. The structure is two-dimensional and consists of one $Si_2OBr_4Cl_2$ cluster and one $Li_3SiBr_4Cl_3$ sheet oriented in the (0, 0, 1) direction. In the $Si_2OBr_4Cl_2$ cluster, there are two inequivalent Si^{4+} sites. In the first Si^{4+} site, $Si(1)^{4+}$ is bonded to one $O(1)^{2-}$, one $Br(1)^{1-}$, one $Br(7)^{1-}$, and one $Cl(2)^{1-}$ atom to form corner-sharing $SiBr_2ClO$ tetrahedra. The Si(1)-O(1) bond length is 1.61 Å. The Si(1)-Br(1) bond length is 2.19 Å. The Si(1)-Br(7) bond length is 2.19 Å. The Si(1)-Cl(2) bond length is 2.02 Å. In the second Si^{4+} site, $Si(2)^{4+}$ is bonded to one $O(1)^{2-}$, one $Br(3)^{1-}$, one $Br(8)^{1-}$, and one $Cl(1)^{1-}$ atom to form corner-sharing $SiBr_2ClO$ tetrahedra. The Si(2)-O(1) bond length is 1.62 Å. The Si(2)-Br(3) bond length is 2.19 Å. The Si(2)-Br(8) bond length is 2.18 Å. The Si(2)-Cl(1) bond length is 2.02 Å. $O(1)^{2-}$ is bonded in a bent 150 degrees geometry to one $Si(1)^{4+}$ and one $Si(2)^{4+}$ atom. There are four inequivalent Br^{1-} sites. In the first Br^{1-} site, $Br(1)^{1-}$ is bonded in a single-bond geometry to one $Si(1)^{4+}$ atom. In the second Br^{1-} site, $Br(3)^{1-}$ is bonded in a single-bond geometry to one $Si(2)^{4+}$ atom. In the third Br^{1-} site, $Br(7)^{1-}$ is bonded in a single-bond geometry to one $Si(1)^{4+}$ atom. In the fourth Br^{1-} site, $Br(8)^{1-}$ is bonded in a single-bond geometry to one $Si(2)^{4+}$ atom. There are two inequivalent Cl^{1-} sites. In the first Cl^{1-} site, $Cl(1)^{1-}$ is bonded in a single-bond geometry to one $Si(2)^{4+}$ atom. In the second Cl^{1-} site, $Cl(2)^{1-}$ is bonded in a single-bond geometry to one $Si(1)^{4+}$ atom. In the $Li_3SiBr_4Cl_3$ sheet, there are three inequivalent Li^{1+} sites. In the first Li^{1+} site, $Li(1)^{1+}$ is bonded in a distorted trigonal planar geometry to one $Br(2)^{1-}$, one $Br(4)^{1-}$, and one $Br(5)^{1-}$ atom. The Li(1)-Br(2) bond length is 2.45 Å. The Li(1)-Br(4) bond length is 2.39 Å. The Li(1)-Br(5) bond length is 2.44 Å. In the second Li^{1+} site, $Li(2)^{1+}$ is bonded in a trigonal planar geometry to one $Br(2)^{1-}$, one $Br(4)^{1-}$, and one $Br(5)^{1-}$ atom. The Li(2)-Br(2) bond length is 2.44 Å. The Li(2)-Br(4) bond length is 2.44 Å. The Li(2)-Br(5) bond length is 2.38 Å. In the third Li^{1+} site, $Li(3)^{1+}$ is bonded to one $Br(2)^{1-}$, one $Br(4)^{1-}$, one $Br(5)^{1-}$, and one $Br(6)^{1-}$ atom to form $LiBr_4$ tetrahedra that share a corner with one $Si(3)BrCl_3$ tetrahedra. The Li(3)-Br(2) bond length is 2.54 Å. The Li(3)-Br(4) bond length is 2.42 Å. The Li(3)-Br(5) bond length is 2.43 Å. The Li(3)-Br(6) bond length is 2.73 Å. $Si(3)^{4+}$ is bonded to one $Br(6)^{1-}$, one $Cl(3)^{1-}$, one $Cl(4)^{1-}$, and one $Cl(5)^{1-}$ atom to form $SiBrCl_3$ tetrahedra that share a corner with one $Li(3)Br_4$ tetrahedra. The Si(3)-Br(6) bond length is 2.21 Å. The Si(3)-Cl(3) bond length is 2.01 Å. The Si(3)-Cl(4) bond length is 2.02 Å. The Si(3)-Cl(5) bond length is 2.01 Å. There are four inequivalent Br^{1-} sites. In the first Br^{1-} site, $Br(2)^{1-}$ is bonded in a 3-coordinate geometry to one $Li(1)^{1+}$, one $Li(2)^{1+}$, and one $Li(3)^{1+}$ atom. In the second Br^{1-} site, $Br(4)^{1-}$ is bonded in a distorted trigonal planar geometry to one $Li(1)^{1+}$, one $Li(2)^{1+}$, and one $Li(3)^{1+}$ atom. In the third Br^{1-} site, $Br(5)^{1-}$ is bonded in a 3-coordinate geometry to one $Li(1)^{1+}$, one $Li(2)^{1+}$, and one $Li(3)^{1+}$ atom. In the fourth Br^{1-} site, $Br(6)^{1-}$ is bonded in a water-like geometry to one $Li(3)^{1+}$ and one $Si(3)^{4+}$ atom. There are three inequivalent Cl^{1-} sites. In the first Cl^{1-} site, $Cl(3)^{1-}$ is bonded in a single-bond geometry to one $Si(3)^{4+}$ atom. In the second Cl^{1-} site, $Cl(4)^{1-}$ is bonded in a single-bond geometry to one $Si(3)^{4+}$ atom. In the third Cl^{1-} site, $Cl(5)^{1-}$ is bonded in a single-bond geometry to one $Si(3)^{4+}$ atom.

4. **$Li_7Al_2S_2Br_8Cl$** crystallizes in the triclinic P1 space group. There are seven inequivalent Li^{1+} sites. In the first Li^{1+} site, $Li(1)^{1+}$ is bonded to one $Br(1)^{1-}$, one $Br(6)^{1-}$, and two equivalent $Br(3)^{1-}$ atoms to form $LiBr_4$ tetrahedra that share corners with two equivalent $Li(4)SBr_4$ square pyramids, a corner with one $Li(3)Br_2Cl_2$ tetrahedra, a corner with one $Al(1)S_2Br_2$ tetrahedra, corners with two equivalent $Li(1)Br_4$ tetrahedra, and corners with two equivalent $Li(2)Br_5$ trigonal bipyramids. The Li(1)-Br(1) bond length is 2.54 Å. The Li(1)-Br(6) bond length is 2.60 Å. There is one shorter (2.43 Å) and one longer (2.44 Å) Li(1)-Br(3) bond length. In the second Li^{1+} site, $Li(2)^{1+}$ is bonded to one $Br(3)^{1-}$, one $Br(7)^{1-}$, one $Br(8)^{1-}$, and two equivalent $Br(4)^{1-}$ atoms to form distorted $LiBr_5$ trigonal bipyramids that share corners with two equivalent $Li(6)S_2Br_4$ octahedra, a corner with one $Li(3)Br_2Cl_2$ tetrahedra, corners with two equivalent $Li(1)Br_4$ tetrahedra, corners with two equivalent $Li(2)Br_5$ trigonal bipyramids, and an edge with one $Al(2)S_2Br_2$ tetrahedra. The corner-sharing octahedral tilt angles range from

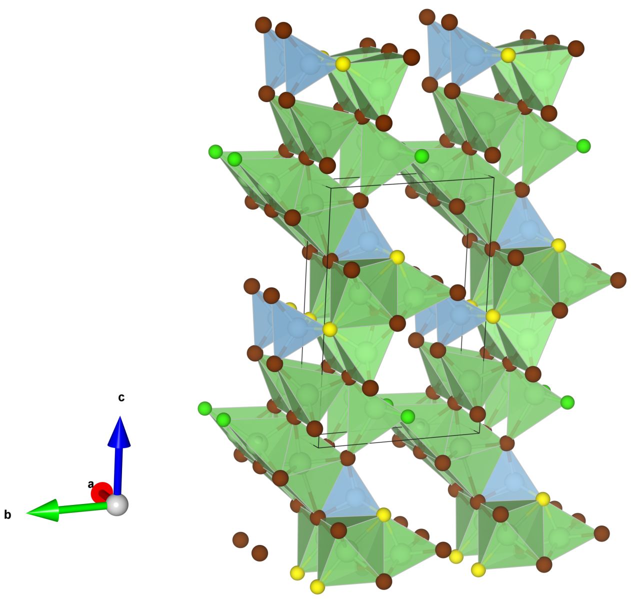

Figure S4: The predicted structure of $Li_7Al_2S_2Br_8Cl$.

39-55°. The Li(2)-Br(3) bond length is 2.60 Å. The Li(2)-Br(7) bond length is 2.62 Å. The Li(2)-Br(8) bond length is 3.18 Å. Both Li(2)-Br(4) bond lengths are 2.56 Å. In the third Li^{1+} site, $Li(3)^{1+}$ is bonded to one $Br(1)^{1-}$, one $Br(7)^{1-}$, and two equivalent $Cl(1)^{1-}$ atoms to form distorted $LiBr_2Cl_2$ tetrahedra that share corners with two equivalent $Li(4)SBr_4$ square pyramids, a corner with one $Li(1)Br_4$ tetrahedra, a corner with one $Al(2)S_2Br_2$ tetrahedra, corners with two equivalent $Li(3)Br_2Cl_2$ tetrahedra, and a corner with one $Li(2)Br_5$ trigonal bipyramid. The Li(3)-Br(1) bond length is 2.49 Å. The Li(3)-Br(7) bond length is 3.31 Å. There is one shorter (2.25 Å) and one longer (2.26 Å) Li(3)-Cl(1) bond length. In the fourth Li^{1+} site, $Li(4)^{1+}$ is bonded to one $S(1)^{2-}$, two equivalent $Br(1)^{1-}$, and two equivalent $Br(2)^{1-}$ atoms to form distorted $LiSBr_4$ square pyramids that share corners with two equivalent $Li(6)S_2Br_4$ octahedra, corners with two equivalent $Li(5)SBr_4$ square pyramids, corners with two equivalent $Li(3)Br_2Cl_2$ tetrahedra, corners with two equivalent $Li(1)Br_4$ tetrahedra, corners with two equivalent $Al(1)S_2Br_2$ tetrahedra, an edge with one $Li(5)SBr_4$ square pyramid, edges with two equivalent $Li(4)SBr_4$ square pyramids, and a faceface with one $Li(6)S_2Br_4$ octahedra. The corner-sharing octahedral tilt angles range from 55-57°. The Li(4)-S(1) bond length is 2.51 Å. Both Li(4)-Br(1) bond lengths are 2.51 Å. There is one shorter (2.79 Å) and one longer (2.95 Å) Li(4)-Br(2) bond length. In the fifth Li^{1+} site, $Li(5)^{1+}$ is bonded to one $S(2)^{2-}$, two equivalent $Br(2)^{1-}$, and two equivalent $Br(5)^{1-}$ atoms to form $LiSBr_4$ square pyramids that share corners with two equivalent $Li(6)S_2Br_4$ octahedra, corners with two equivalent $Li(4)SBr_4$ square pyramids, corners with two equivalent $Al(1)S_2Br_2$ tetrahedra, corners with two equivalent $Al(2)S_2Br_2$ tetrahedra, an edge with one $Li(4)SBr_4$ square pyramid, edges with two equivalent $Li(5)SBr_4$ square pyramids, and a faceface with one $Li(6)S_2Br_4$ octahedra. The corner-sharing octahedral tilt angles are 53°. The Li(5)-S(2) bond length is 2.50 Å. There is one shorter (2.67 Å) and one longer (2.69 Å) Li(5)-Br(2) bond length. There is one shorter (2.65 Å) and one longer (2.67 Å) Li(5)-Br(5) bond length. In the sixth Li^{1+} site, $Li(6)^{1+}$ is bonded to one $S(1)^{2-}$, one $S(2)^{2-}$, two equivalent $Br(2)^{1-}$, and two equivalent $Br(8)^{1-}$ atoms to form LiS_2Br_4 octahedra that share corners with two equivalent $Li(4)SBr_4$ square pyramids, corners with two equivalent $Li(5)SBr_4$ square pyramids, corners with two equivalent $Al(1)S_2Br_2$ tetrahedra, corners with two equivalent $Li(2)Br_5$ trigonal bipyramids, edges with two equivalent $Li(6)S_2Br_4$ octahedra, edges with two equivalent $Al(2)S_2Br_2$ tetrahedra, a faceface with one $Li(4)SBr_4$ square pyramid, and a faceface with one $Li(5)SBr_4$ square pyramid. The Li(6)-S(1) bond length is 2.53

Å. The Li(6)-S(2) bond length is 2.83 Å. There is one shorter (2.79 Å) and one longer (2.80 Å) Li(6)-Br(2) bond length. There is one shorter (2.65 Å) and one longer (2.70 Å) Li(6)-Br(8) bond length. In the seventh Li^{1+} site, $Li(7)^{1+}$ is bonded in a rectangular see-saw-like geometry to one $Br(3)^{1-}$, two equivalent $Br(4)^{1-}$, and one $Cl(1)^{1-}$ atom. The Li(7)-Br(3) bond length is 2.55 Å. There is one shorter (2.58 Å) and one longer (2.60 Å) Li(7)-Br(4) bond length. The Li(7)-Cl(1) bond length is 2.28 Å. There are two inequivalent Al3+ sites. In the first Al^{3+} site, $Al(1)^{3+}$ is bonded to two equivalent $S(1)^{2-}$, one $Br(5)^{1-}$, and one $Br(6)^{1-}$ atom to form AlS_2Br_2 tetrahedra that share corners with two equivalent $Li(6)S_2Br_4$ octahedra, corners with two equivalent $Li(4)SBr_4$ square pyramids, corners with two equivalent $Li(5)SBr_4$ square pyramids, a corner with one $Li(1)Br_4$ tetrahedra, and corners with two equivalent $Al(1)S_2Br_2$ tetrahedra. The corner-sharing octahedral tilt angles range from 65-66°. There is one shorter (2.25 Å) and one longer (2.26 Å) Al(1)-S(1) bond length. The Al(1)-Br(5) bond length is 2.35 Å. The Al(1)-Br(6) bond length is 2.31 Å. In the second Al^{3+} site, $Al(2)^{3+}$ is bonded to two equivalent $S(2)^{2-}$, one $Br(7)^{1-}$, and one $Br(8)^{1-}$ atom to form AlS_2Br_2 tetrahedra that share corners with two equivalent $Li(5)SBr_4$ square pyramids, a corner with one $Li(3)Br_2Cl_2$ tetrahedra, corners with two equivalent $Al(2)S_2Br_2$ tetrahedra, edges with two equivalent $Li(6)S_2Br_4$ octahedra, and an edge with one $Li(2)Br_5$ trigonal bipyramid. Both Al(2)-S(2) bond lengths are 2.24 Å. The Al(2)-Br(7) bond length is 2.32 Å. The Al(2)-Br(8) bond length is 2.37 Å. There are two inequivalent S^{2-} sites. In the first S^{2-} site, $S(1)^{2-}$ is bonded in a tetrahedral geometry to one $Li(4)^{1+}$, one $Li(6)^{1+}$, and two equivalent $Al(1)^{3+}$ atoms. In the second S^{2-} site, $S(2)^{2-}$ is bonded in a distorted trigonal pyramidal geometry to one $Li(5)^{1+}$, one $Li(6)^{1+}$, and two equivalent $Al(2)^{3+}$ atoms. There are eight inequivalent Br^{1-} sites. In the first Br^{1-} site, $Br(1)^{1-}$ is bonded in a tetrahedral geometry to one $Li(1)^{1+}$, one $Li(3)^{1+}$, and two equivalent $Li(4)^{1+}$ atoms. In the second Br^{1-} site, $Br(2)^{1-}$ is bonded in a 6-coordinate geometry to two equivalent $Li(4)^{1+}$, two equivalent $Li(5)^{1+}$, and two equivalent $Li(6)^{1+}$ atoms. In the third Br^{1-} site, $Br(3)^{1-}$ is bonded in a distorted tetrahedral geometry to one $Li(2)^{1+}$, one $Li(7)^{1+}$, and two equivalent $Li(1)^{1+}$ atoms. In the fourth Br^{1-} site, $Br(4)^{1-}$ is bonded in a 4-coordinate geometry to two equivalent $Li(2)^{1+}$ and two equivalent $Li(7)^{1+}$ atoms. In the fifth Br^{1-} site, $Br(5)^{1-}$ is bonded in a trigonal non-coplanar geometry to two equivalent $Li(5)^{1+}$ and one $Al(1)^{3+}$ atom. In the sixth Br^{1-} site, $Br(6)^{1-}$ is bonded in a water-like geometry to one $Li(1)^{1+}$ and one $Al(1)^{3+}$ atom. In the seventh Br^{1-} site, $Br(7)^{1-}$ is bonded in a 3-coordinate geometry to one $Li(2)^{1+}$, one $Li(3)^{1+}$, and one $Al(2)^{3+}$ atom. In the eighth Br^{1-} site, $Br(8)^{1-}$ is bonded in a distorted rectangular see-saw-like geometry to one $Li(2)^{1+}$, two equivalent $Li(6)^{1+}$, and one $Al(2)^{3+}$ atom. $Cl(1)^{1-}$ is bonded in a trigonal non-coplanar geometry to one $Li(7)^{1+}$ and two equivalent $Li(3)^{1+}$ atoms.

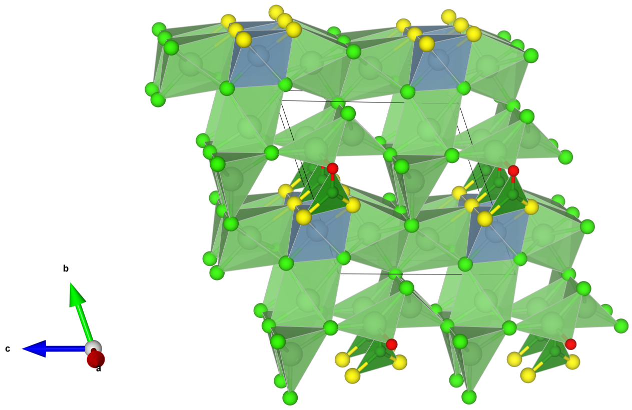

Figure S5: The predicted structure of $Li_6AlBS_2OCl_6$.

5. **$Li_6AlBS_2OCl_6$** is Aluminum carbonitride-derived structured and crystallizes in the triclinic P1

space group. There are six inequivalent Li^{1+} sites. In the first Li^{1+} site, $Li(1)^{1+}$ is bonded to one $S(2)^{2-}$, one $Cl(5)^{1-}$, two equivalent $Cl(1)^{1-}$, and two equivalent $Cl(6)^{1-}$ atoms to form distorted $LiSCl_5$ octahedra that share corners with two equivalent $Li(5)SCl_5$ octahedra, corners with two equivalent $Li(6)Cl_3O$ tetrahedra, corners with two equivalent $Li(4)Cl_4$ tetrahedra, a corner with one $Al(1)S_3Cl_2$ trigonal bipyramid, corners with two equivalent $Li(2)Cl_5$ trigonal bipyramids, a corner with one $Li(3)Cl_2O_2$ trigonal pyramid, edges with two equivalent $Li(1)SCl_5$ octahedra, an edge with one $Al(1)S_3Cl_2$ trigonal bipyramid, and a faceface with one $Li(5)SCl_5$ octahedra. The corner-sharing octahedral tilt angles range from 44-53°. The Li(1)-S(2) bond length is 2.60 Å. The Li(1)-Cl(5) bond length is 2.50 Å. There is one shorter (2.60 Å) and one longer (2.64 Å) Li(1)-Cl(1) bond length. There is one shorter (2.44 Å) and one longer (3.14 Å) Li(1)-Cl(6) bond length. In the second Li^{1+} site, $Li(2)^{1+}$ is bonded to one $Cl(1)^{1-}$, one $Cl(3)^{1-}$, one $Cl(4)^{1-}$, and two equivalent $Cl(2)^{1-}$ atoms to form distorted $LiCl_5$ trigonal bipyramids that share corners with two equivalent $Li(1)SCl_5$ octahedra, corners with two equivalent $Li(5)SCl_5$ octahedra, a corner with one $Li(6)Cl_3O$ tetrahedra, corners with two equivalent $Li(2)Cl_5$ trigonal bipyramids, corners with two equivalent $Li(3)Cl_2O_2$ trigonal pyramids, edges with two equivalent $Li(4)Cl_4$ tetrahedra, and an edge with one $Al(1)S_3Cl_2$ trigonal bipyramid. The corner-sharing octahedral tilt angles range from 40-63°. The Li(2)-Cl(1) bond length is 2.87 Å. The Li(2)-Cl(3) bond length is 2.40 Å. The Li(2)-Cl(4) bond length is 2.46 Å. There is one shorter (2.31 Å) and one longer (2.34 Å) Li(2)-Cl(2) bond length. In the third Li^{1+} site, $Li(3)^{1+}$ is bonded to two equivalent $O(1)^{2-}$, one $Cl(2)^{1-}$, and one $Cl(5)^{1-}$ atom to form distorted $LiCl_2O_2$ trigonal pyramids that share a corner with one $Li(1)SCl_5$ octahedra, a corner with one $Li(5)SCl_5$ octahedra, a corner with one $Li(4)Cl_4$ tetrahedra, corners with two equivalent $Li(2)Cl_5$ trigonal bipyramids, corners with two equivalent $Li(3)Cl_2O_2$ trigonal pyramids, and edges with two equivalent $Li(6)Cl_3O$ tetrahedra. The corner-sharing octahedral tilt angles range from 3-75°. There is one shorter (1.99 Å) and one longer (2.13 Å) Li(3)-O(1) bond length. The Li(3)-Cl(2) bond length is 2.31 Å. The Li(3)-Cl(5) bond length is 2.47 Å. In the fourth Li^{1+} site, $Li(4)^{1+}$ is bonded to one $Cl(2)^{1-}$, one $Cl(6)^{1-}$, and two equivalent $Cl(3)^{1-}$ atoms to form distorted $LiCl_4$ tetrahedra that share corners with two equivalent $Li(1)SCl_5$ octahedra, corners with two equivalent $Li(5)SCl_5$ octahedra, corners with two equivalent $Li(6)Cl_3O$ tetrahedra, corners with two equivalent $Li(4)Cl_4$ tetrahedra, a corner with one $Li(3)Cl_2O_2$ trigonal pyramid, and edges with two equivalent $Li(2)Cl_5$ trigonal bipyramids. The corner-sharing octahedral tilt angles range from 58-72°. The Li(4)-Cl(2) bond length is 2.44 Å. The Li(4)-Cl(6) bond length is 2.36 Å. Both Li(4)-Cl(3) bond lengths are 2.33 Å. In the fifth Li^{1+} site, $Li(5)^{1+}$ is bonded to one $S(1)^{2-}$, one $Cl(5)^{1-}$, two equivalent $Cl(4)^{1-}$, and two equivalent $Cl(6)^{1-}$ atoms to form distorted $LiSCl_5$ octahedra that share corners with two equivalent $Li(1)SCl_5$ octahedra, corners with two equivalent $Li(6)Cl_3O$ tetrahedra, corners with two equivalent $Li(4)Cl_4$ tetrahedra, corners with two equivalent $Li(2)Cl_5$ trigonal bipyramids, a corner with one $Li(3)Cl_2O_2$ trigonal pyramid, edges with two equivalent $Li(5)SCl_5$ octahedra, edges with two equivalent $Al(1)S_3Cl_2$ trigonal bipyramids, and a faceface with one $Li(1)SCl_5$ octahedra. The corner-sharing octahedral tilt angles range from 44-53°. The Li(5)-S(1) bond length is 2.91 Å. The Li(5)-Cl(5) bond length is 2.50 Å. There is one shorter (2.56 Å) and one longer (2.80 Å) Li(5)-Cl(4) bond length. There is one shorter (2.43 Å) and one longer (2.47 Å) Li(5)-Cl(6) bond length. In the sixth Li^{1+} site, $Li(6)^{1+}$ is bonded to one $O(1)^{2-}$, one $Cl(3)^{1-}$, and two equivalent $Cl(5)^{1-}$ atoms to form distorted $LiCl_3O$ tetrahedra that share corners with two equivalent $Li(1)SCl_5$ octahedra, corners with two equivalent $Li(5)SCl_5$ octahedra, corners with two equivalent $Li(6)Cl_3O$ tetrahedra, corners with two equivalent $Li(4)Cl_4$ tetrahedra, a corner with one $Li(2)Cl_5$ trigonal bipyramid, and edges with two equivalent $Li(3)Cl_2O_2$ trigonal pyramids. The corner-sharing octahedral tilt angles range from 44-77°. The Li(6)-O(1) bond length is 1.92 Å. The Li(6)-Cl(3) bond length is 2.30 Å. There is one shorter (2.36 Å) and one longer (2.37 Å) Li(6)-Cl(5) bond length. $Al(1)^{3+}$ is bonded to one $S(2)^{2-}$, two equivalent $S(1)^{2-}$, one $Cl(1)^{1-}$, and one $Cl(4)^{1-}$ atom to form AlS_3Cl_2 trigonal bipyramids that share a corner with one $Li(1)SCl_5$ octahedra, corners with two

equivalent $Al(1)S_3Cl_2$ trigonal bipyramids, an edge with one $Li(1)SCl_5$ octahedra, edges with two equivalent $Li(5)SCl_5$ octahedra, and an edge with one $Li(2)Cl_5$ trigonal bipyramid. The corner-sharing octahedral tilt angles are 66°. The Al(1)-S(2) bond length is 2.30 Å. There is one shorter (2.31 Å) and one longer (2.39 Å) Al(1)-S(1) bond length. The Al(1)-Cl(1) bond length is 2.29 Å. The Al(1)-Cl(4) bond length is 2.31 Å. $B(1)^{3+}$ is bonded in a distorted trigonal planar geometry to one $S(1)^{2-}$, one $S(2)^{2-}$, and one $O(1)^{2-}$ atom. The B(1)-S(1) bond length is 1.95 Å. The B(1)-S(2) bond length is 1.83 Å. The B(1)-O(1) bond length is 1.31 Å. There are two inequivalent S^{2-} sites. In the first S^{2-} site, $S(1)^{2-}$ is bonded in a distorted see-saw-like geometry to one $Li(5)^{1+}$, two equivalent $Al(1)^{3+}$, and one $B(1)^{3+}$ atom. In the second S^{2-} site, $S(2)^{2-}$ is bonded in a 3-coordinate geometry to one $Li(1)^{1+}$, one $Al(1)^{3+}$, and one $B(1)^{3+}$ atom. $O(1)^{2-}$ is bonded in a distorted trigonal pyramidal geometry to one $Li(6)^{1+}$, two equivalent $Li(3)^{1+}$, and one $B(1)^{3+}$ atom. There are six inequivalent Cl^{1-} sites. In the first Cl^{1-} site, $Cl(1)^{1-}$ is bonded in a distorted rectangular see-saw-like geometry to one $Li(2)^{1+}$, two equivalent $Li(1)^{1+}$, and one $Al(1)^{3+}$ atom. In the second Cl^{1-} site, $Cl(2)^{1-}$ is bonded in a distorted tetrahedral geometry to one $Li(3)^{1+}$, one $Li(4)^{1+}$, and two equivalent $Li(2)^{1+}$ atoms. In the third Cl^{1-} site, $Cl(3)^{1-}$ is bonded in a distorted trigonal pyramidal geometry to one $Li(2)^{1+}$, one $Li(6)^{1+}$, and two equivalent $Li(4)^{1+}$ atoms. In the fourth Cl^{1-} site, $Cl(4)^{1-}$ is bonded in a distorted rectangular see-saw-like geometry to one $Li(2)^{1+}$, two equivalent $Li(5)^{1+}$, and one $Al(1)^{3+}$ atom. In the fifth Cl^{1-} site, $Cl(5)^{1-}$ is bonded in a distorted trigonal bipyramidal geometry to one $Li(1)^{1+}$, one $Li(3)^{1+}$, one $Li(5)^{1+}$, and two equivalent $Li(6)^{1+}$ atoms. In the sixth Cl^{1-} site, $Cl(6)^{1-}$ is bonded in a 4-coordinate geometry to one $Li(4)^{1+}$, two equivalent $Li(1)^{1+}$, and two equivalent $Li(5)^{1+}$ atoms.

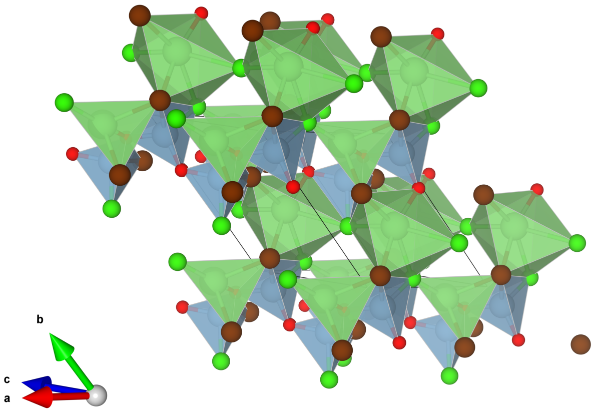

Figure S6: The predicted structure of LiAlOBrCl.

6. **LiAlOBrCl** crystallizes in the triclinic P1 space group. There are two inequivalent Li^{1+} sites. In the first Li^{1+} site, $Li(1)^{1+}$ is bonded in a 6-coordinate geometry to one $O(2)^{2-}$, one $Br(1)^{1-}$, one $Br(2)^{1-}$, one $Cl(1)^{1-}$, and two equivalent $Cl(2)^{1-}$ atoms. The Li(1)-O(2) bond length is 1.92 Å. The Li(1)-Br(1) bond length is 2.67 Å. The Li(1)-Br(2) bond length is 3.11 Å. The Li(1)-Cl(1) bond length is 2.94 Å. There is one shorter (2.42 Å) and one longer (3.00 Å) Li(1)-Cl(2) bond length. In the second Li^{1+} site, $Li(2)^{1+}$ is bonded in a 5-coordinate geometry to one $O(1)^{2-}$, one $Br(2)^{1-}$, two equivalent $Br(1)^{1-}$, and one $Cl(1)^{1-}$ atom. The Li(2)-O(1) bond length is 1.92 Å. The Li(2)-Br(2) bond length is 2.83 Å. There is one shorter (2.75 Å) and one longer (3.28 Å) Li(2)-Br(1) bond length. The Li(2)-Cl(1) bond length is 2.43 Å. There are two inequivalent Al^{3+} sites. In the first Al^{3+} site, $Al(1)^{3+}$ is bonded to one $O(1)^{2-}$, one $O(2)^{2-}$, one $Br(2)^{1-}$, and one $Cl(2)^{1-}$ atom to form corner-sharing $AlBrClO_2$ tetrahedra. The Al(1)-O(1) bond length is 1.72 Å. The Al(1)-O(2) bond length is 1.73 Å. The Al(1)-Br(2) bond length is 2.34 Å. The Al(1)-Cl(2) bond length is 2.18 Å. In the second Al^{3+} site, $Al(2)^{3+}$ is bonded to one $O(1)^{2-}$, one $O(2)^{2-}$, one $Br(1)^{1-}$, and one $Cl(1)^{1-}$ atom to form distorted

corner-sharing $AlBrClO_2$ tetrahedra. The Al(2)-O(1) bond length is 1.72 Å. The Al(2)-O(2) bond length is 1.72 Å. The Al(2)-Br(1) bond length is 2.36 Å. The Al(2)-Cl(1) bond length is 2.18 Å. There are two inequivalent O^{2-} sites. In the first O^{2-} site, $O(1)^{2-}$ is bonded in a trigonal planar geometry to one $Li(2)^{1+}$, one $Al(1)^{3+}$, and one $Al(2)^{3+}$ atom. In the second O^{2-} site, $O(2)^{2-}$ is bonded in a trigonal planar geometry to one $Li(1)^{1+}$, one $Al(1)^{3+}$, and one $Al(2)^{3+}$ atom. There are two inequivalent Br^{1-} sites. In the first Br^{1-} site, $Br(1)^{1-}$ is bonded in a 4-coordinate geometry to one $Li(1)^{1+}$, two equivalent $Li(2)^{1+}$, and one $Al(2)^{3+}$ atom. In the second Br^{1-} site, $Br(2)^{1-}$ is bonded in a 3-coordinate geometry to one $Li(1)^{1+}$, one $Li(2)^{1+}$, and one $Al(1)^{3+}$ atom. There are two inequivalent Cl^{1-} sites. In the first Cl^{1-} site, $Cl(1)^{1-}$ is bonded in a 3-coordinate geometry to one $Li(1)^{1+}$, one $Li(2)^{1+}$, and one $Al(2)^{3+}$ atom. In the second Cl^{1-} site, $Cl(2)^{1-}$ is bonded in a distorted water-like geometry to two equivalent $Li(1)^{1+}$ and one $Al(1)^{3+}$ atom.

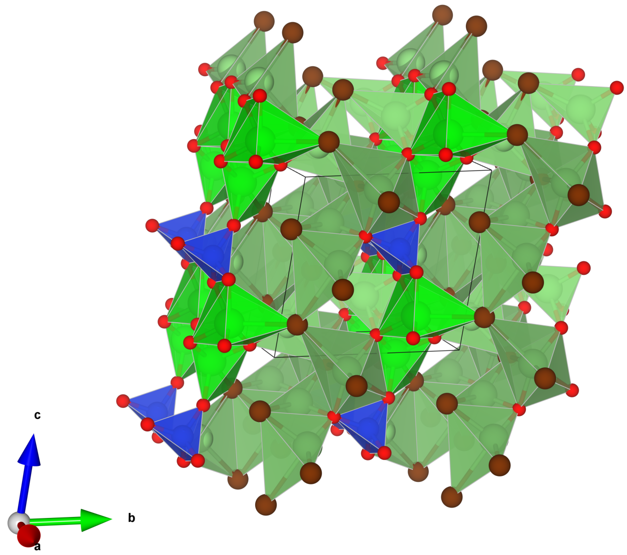

Figure S7: The predicted structure of $Li_6Zr_2SiO_7Br_4$.

7. **$Li_6Zr_2SiO_7Br_4$** crystallizes in the triclinic P1 space group. There are six inequivalent Li^{1+} sites. In the first Li^{1+} site, $Li(1)^{1+}$ is bonded in a 4-coordinate geometry to one $O(7)^{2-}$, one $Br(1)^{1-}$, one $Br(3)^{1-}$, and one $Br(4)^{1-}$ atom. The Li(1)-O(7) bond length is 1.86 Å. The Li(1)-Br(1) bond length is 2.42 Å. The Li(1)-Br(3) bond length is 2.50 Å. The Li(1)-Br(4) bond length is 3.11 Å. In the second Li^{1+} site, $Li(2)^{1+}$ is bonded in a 2-coordinate geometry to one $O(4)^{2-}$, one $O(6)^{2-}$, one $Br(3)^{1-}$, and one $Br(4)^{1-}$ atom. The Li(2)-O(4) bond length is 1.88 Å. The Li(2)-O(6) bond length is 1.94 Å. The Li(2)-Br(3) bond length is 2.66 Å. The Li(2)-Br(4) bond length is 3.02 Å. In the third Li^{1+} site, $Li(3)^{1+}$ is bonded to one $O(2)^{2-}$, one $O(5)^{2-}$, one $O(6)^{2-}$, and one $Br(1)^{1-}$ atom to form distorted $LiBrO_3$ trigonal pyramids that share a corner with one $Si(1)O_4$ tetrahedra. The Li(3)-O(2) bond length is 1.96 Å. The Li(3)-O(5) bond length is 2.13 Å. The Li(3)-O(6) bond length is 1.89 Å. The Li(3)-Br(1) bond length is 2.48 Å. In the fourth Li^{1+} site, $Li(4)^{1+}$ is bonded in a 6-coordinate geometry to one $O(1)^{2-}$, one $O(5)^{2-}$, one $O(7)^{2-}$, one $Br(4)^{1-}$, and two equivalent $Br(2)^{1-}$ atoms. The Li(4)-O(1) bond length is 2.57 Å. The Li(4)-O(5) bond length is 2.02 Å. The Li(4)-O(7) bond length is 2.09 Å. The Li(4)-Br(4) bond length is 3.01 Å. There is one shorter (2.57 Å) and one longer (3.08 Å) Li(4)-Br(2) bond length. In the fifth Li^{1+} site, $Li(5)^{1+}$ is bonded in a 4-coordinate geometry to one $O(7)^{2-}$, one $Br(1)^{1-}$, one $Br(2)^{1-}$, and one $Br(3)^{1-}$ atom. The Li(5)-O(7) bond length is 1.87 Å. The Li(5)-Br(1) bond length is 2.53 Å. The Li(5)-Br(2) bond length is 2.46 Å. The Li(5)-Br(3) bond length is 2.71 Å. In the sixth Li^{1+} site, $Li(6)^{1+}$ is bonded in a 1-coordinate geometry to one $O(3)^{2-}$, one $Br(1)^{1-}$, one $Br(2)^{1-}$, and two equivalent $Br(4)^{1-}$ atoms. The Li(6)-O(3) bond length is 1.89

Å. The Li(6)-Br(1) bond length is 2.60 Å. The Li(6)-Br(2) bond length is 2.49 Å. There is one shorter (2.57 Å) and one longer (3.15 Å) Li(6)-Br(4) bond length. There are two inequivalent Zr^{4+} sites. In the first Zr^{4+} site, $Zr(1)^{4+}$ is bonded in a 6-coordinate geometry to one $O(2)^{2-}$, one $O(3)^{2-}$, one $O(4)^{2-}$, one $O(5)^{2-}$, one $O(6)^{2-}$, and one $Br(4)^{1-}$ atom. The Zr(1)-O(2) bond length is 1.98 Å. The Zr(1)-O(3) bond length is 2.07 Å. The Zr(1)-O(4) bond length is 2.11 Å. The Zr(1)-O(5) bond length is 2.04 Å. The Zr(1)-O(6) bond length is 2.26 Å. The Zr(1)-Br(4) bond length is 2.93 Å. In the second Zr^{4+} site, $Zr(2)^{4+}$ is bonded in a 4-coordinate geometry to one $O(1)^{2-}$, one $O(2)^{2-}$, one $O(3)^{2-}$, one $O(5)^{2-}$, and one $Br(3)^{1-}$ atom. The Zr(2)-O(1) bond length is 2.02 Å. The Zr(2)-O(2) bond length is 2.03 Å. The Zr(2)-O(3) bond length is 1.98 Å. The Zr(2)-O(5) bond length is 2.03 Å. The Zr(2)-Br(3) bond length is 2.79 Å. $Si(1)^{4+}$ is bonded to one $O(1)^{2-}$, one $O(4)^{2-}$, one $O(6)^{2-}$, and one $O(7)^{2-}$ atom to form SiO_4 tetrahedra that share a corner with one $Li(3)BrO_3$ trigonal pyramid. The Si(1)-O(1) bond length is 1.62 Å. The Si(1)-O(4) bond length is 1.65 Å. The Si(1)-O(6) bond length is 1.65 Å. The Si(1)-O(7) bond length is 1.61 Å. There are seven inequivalent O^{2-} sites. In the first O^{2-} site, $O(1)^{2-}$ is bonded in a 2-coordinate geometry to one $Li(4)^{1+}$, one $Zr(2)^{4+}$, and one $Si(1)^{4+}$ atom. In the second O^{2-} site, $O(2)^{2-}$ is bonded in a 3-coordinate geometry to one $Li(3)^{1+}$, one $Zr(1)^{4+}$, and one $Zr(2)^{4+}$ atom. In the third O^{2-} site, $O(3)^{2-}$ is bonded in a distorted trigonal non-coplanar geometry to one $Li(6)^{1+}$, one $Zr(1)^{4+}$, and one $Zr(2)^{4+}$ atom. In the fourth O^{2-} site, $O(4)^{2-}$ is bonded in a distorted trigonal non-coplanar geometry to one $Li(2)^{1+}$, one $Zr(1)^{4+}$, and one $Si(1)^{4+}$ atom. In the fifth O^{2-} site, $O(5)^{2-}$ is bonded in a trigonal pyramidal geometry to one $Li(3)^{1+}$, one $Li(4)^{1+}$, one $Zr(1)^{4+}$, and one $Zr(2)^{4+}$ atom. In the sixth O^{2-} site, $O(6)^{2-}$ is bonded in a 4-coordinate geometry to one $Li(2)^{1+}$, one $Li(3)^{1+}$, one $Zr(1)^{4+}$, and one $Si(1)^{4+}$ atom. In the seventh O^{2-} site, $O(7)^{2-}$ is bonded in a distorted trigonal pyramidal geometry to one $Li(1)^{1+}$, one $Li(4)^{1+}$, one $Li(5)^{1+}$, and one $Si(1)^{4+}$ atom. There are four inequivalent Br^{1-} sites. In the first Br^{1-} site, $Br(1)^{1-}$ is bonded in a distorted trigonal pyramidal geometry to one $Li(1)^{1+}$, one $Li(3)^{1+}$, one $Li(5)^{1+}$, and one $Li(6)^{1+}$ atom. In the second Br^{1-} site, $Br(2)^{1-}$ is bonded in a 3-coordinate geometry to one $Li(5)^{1+}$, one $Li(6)^{1+}$, and two equivalent $Li(4)^{1+}$ atoms. In the third Br^{1-} site, $Br(3)^{1-}$ is bonded in a 4-coordinate geometry to one $Li(1)^{1+}$, one $Li(2)^{1+}$, one $Li(5)^{1+}$, and one $Zr(2)^{4+}$ atom. In the fourth Br^{1-} site, $Br(4)^{1-}$ is bonded in a 6-coordinate geometry to one $Li(1)^{1+}$, one $Li(2)^{1+}$, one $Li(4)^{1+}$, two equivalent $Li(6)^{1+}$, and one $Zr(1)^{4+}$ atom.

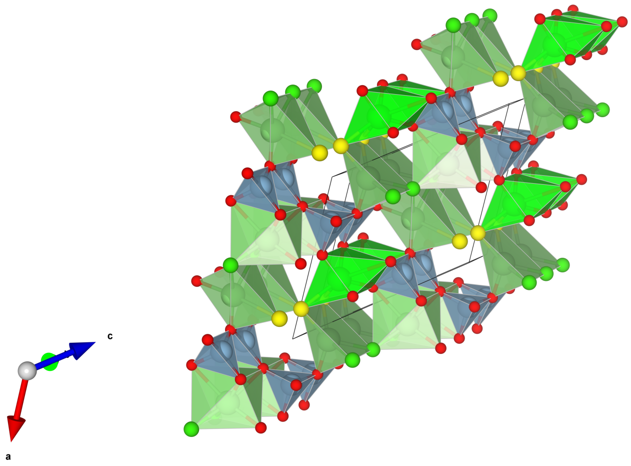

Figure S8: The predicted structure of $Li_3ZrAl_2SO_5Cl$.

8. **$Li_3ZrAl_2SO_5Cl$** is Chalcostibite-derived structured and crystallizes in the triclinic P1 space group. There are three inequivalent Li^{1+},sites. In the first Li^{1+},site, $Li(1)^{1+}$,is bonded in a 5-coordinate geometry to two equivalent $S(1)^{2-}$, one $O(1)^{2-}$, and two equivalent $Cl(1)^{1-}$,atoms.

There is one shorter (2.40 Å) and one longer (2.74 Å) Li(1)-S(1) bond length. The Li(1)-O(1) bond length is 1.92 Å. There is one shorter (2.44 Å) and one longer (2.78 Å) Li(1)-Cl(1) bond length. In the second Li^{1+},site, $Li(2)^{1+}$,is bonded in a 5-coordinate geometry to one $O(2)^{2-}$, one $O(3)^{2-}$, one $O(4)^{2-}$, and two equivalent $Cl(1)^{1-}$,atoms. The Li(2)-O(2) bond length is 1.88 Å. The Li(2)-O(3) bond length is 2.34 Å. The Li(2)-O(4) bond length is 2.71 Å. There is one shorter (2.35 Å) and one longer (2.42 Å) Li(2)-Cl(1) bond length. In the third Li^{1+},site, $Li(3)^{1+}$,is bonded in a 5-coordinate geometry to one $S(1)^{2-}$, one $O(1)^{2-}$, one $O(5)^{2-}$, and two equivalent $Cl(1)^{1-}$,atoms. The Li(3)-S(1) bond length is 3.10 Å. The Li(3)-O(1) bond length is 2.09 Å. The Li(3)-O(5) bond length is 1.88 Å. There is one shorter (2.37 Å) and one longer (2.54 Å) Li(3)-Cl(1) bond length. $Zr(1)^{4+}$,is bonded in a 6-coordinate geometry to two equivalent $S(1)^{2-}$, two equivalent $O(3)^{2-}$, and two equivalent $O(4)^{2-}$,atoms. There is one shorter (2.50 Å) and one longer (2.60 Å) Zr(1)-S(1) bond length. There is one shorter (2.16 Å) and one longer (2.17 Å) Zr(1)-O(3) bond length. There is one shorter (2.08 Å) and one longer (2.13 Å) Zr(1)-O(4) bond length. There are two inequivalent Al^{3+},sites. In the first Al^{3+},site, $Al(1)^{3+}$,is bonded to one $O(2)^{2-}$, one $O(3)^{2-}$, and two equivalent $O(1)^{2-}$,atoms to form corner-sharing AlO_4 tetrahedra. The Al(1)-O(2) bond length is 1.72 Å. The Al(1)-O(3) bond length is 1.80 Å. Both Al(1)-O(1) bond lengths are 1.79 Å. In the second Al^{3+},site, $Al(2)^{3+}$,is bonded to one $O(2)^{2-}$, one $O(4)^{2-}$, and two equivalent $O(5)^{2-}$,atoms to form corner-sharing AlO_4 trigonal pyramids. The Al(2)-O(2) bond length is 1.72 Å. The Al(2)-O(4) bond length is 1.80 Å. There is one shorter (1.77 Å) and one longer (1.78 Å) Al(2)-O(5) bond length. $S(1)^{2-}$,is bonded in a 5-coordinate geometry to one $Li(3)^{1+}$, two equivalent $Li(1)^{1+}$, and two equivalent $Zr(1)^{4+}$,atoms. There are five inequivalent O^{2-},sites. In the first O^{2-},site, $O(1)^{2-}$,is bonded in a tetrahedral geometry to one $Li(1)^{1+}$, one $Li(3)^{1+}$, and two equivalent $Al(1)^{3+}$,atoms. In the second O^{2-},site, $O(2)^{2-}$,is bonded in a distorted T-shaped geometry to one $Li(2)^{1+}$, one $Al(1)^{3+}$, and one $Al(2)^{3+}$,atom. In the third O^{2-},site, $O(3)^{2-}$,is bonded in a 4-coordinate geometry to one $Li(2)^{1+}$, two equivalent $Zr(1)^{4+}$, and one $Al(1)^{3+}$,atom. In the fourth O^{2-},site, $O(4)^{2-}$,is bonded in a 3-coordinate geometry to one $Li(2)^{1+}$, two equivalent $Zr(1)^{4+}$, and one $Al(2)^{3+}$,atom. In the fifth O^{2-},site, $O(5)^{2-}$,is bonded in a trigonal planar geometry to one $Li(3)^{1+}$,and two equivalent $Al(2)^{3+}$,atoms. $Cl(1)^{1-}$,is bonded in a 6-coordinate geometry to two equivalent $Li(1)^{1+}$, two equivalent $Li(2)^{1+}$, and two equivalent $Li(3)^{1+}$,atoms.

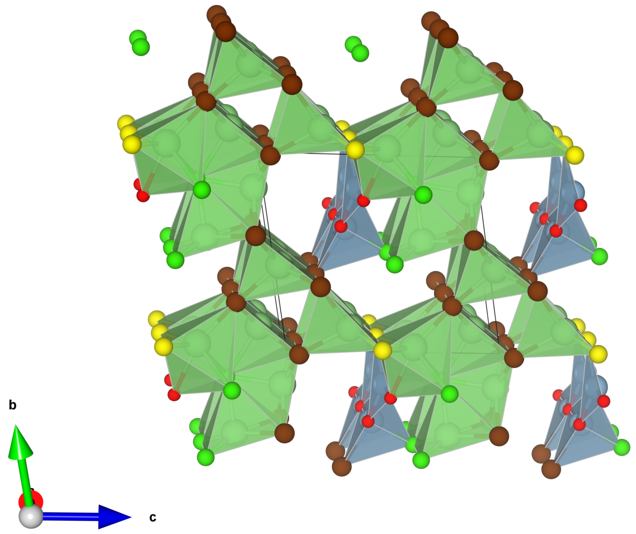

Figure S9: The predicted structure of $Li_6Al_2SBr_4(OCl)_2$.

9. $\mathbf{Li_6Al_2SBr_4(OCl)_2}$ crystallizes in the triclinic P1 space group. There are six inequivalent Li^{1+} sites. In the first Li^{1+} site, $Li(1)^{1+}$ is bonded in a 6-coordinate geometry to two equivalent

$S(1)^{2-}$, one $O(2)^{2-}$, one $Br(2)^{1-}$, and two equivalent $Cl(1)^{1-}$ atoms. There is one shorter (2.38 Å) and one longer (2.39 Å) Li(1)-S(1) bond length. The Li(1)-O(2) bond length is 2.52 Å. The Li(1)-Br(2) bond length is 2.53 Å. There is one shorter (2.93 Å) and one longer (2.95 Å) Li(1)-Cl(1) bond length. In the second Li^{1+} site, $Li(2)^{1+}$ is bonded in a 5-coordinate geometry to two equivalent $S(1)^{2-}$, one $Br(4)^{1-}$, and two equivalent $Br(3)^{1-}$ atoms. There is one shorter (2.34 Å) and one longer (2.35 Å) Li(2)-S(1) bond length. The Li(2)-Br(4) bond length is 2.55 Å. There is one shorter (3.02 Å) and one longer (3.26 Å) Li(2)-Br(3) bond length. In the third Li^{1+} site, $Li(3)^{1+}$ is bonded to two equivalent $Br(2)^{1-}$, two equivalent $Br(4)^{1-}$, and one $Cl(1)^{1-}$ atom to form distorted $LiBr_4Cl$ square pyramids that share corners with two equivalent $Li(6)Br_4Cl$ square pyramids, corners with two equivalent $Li(5)Br_5$ trigonal bipyramids, edges with two equivalent $Li(3)Br_4Cl$ square pyramids, and a faceface with one $Li(6)Br_4Cl$ square pyramid. There is one shorter (2.52 Å) and one longer (2.54 Å) Li(3)-Br(2) bond length. There is one shorter (2.61 Å) and one longer (2.72 Å) Li(3)-Br(4) bond length. The Li(3)-Cl(1) bond length is 2.65 Å. In the fourth Li^{1+} site, $Li(4)^{1+}$ is bonded in a 6-coordinate geometry to one $Br(2)^{1-}$, two equivalent $Br(1)^{1-}$, one $Cl(1)^{1-}$, and two equivalent $Cl(2)^{1-}$ atoms. The Li(4)-Br(2) bond length is 3.22 Å. There is one shorter (2.73 Å) and one longer (2.88 Å) Li(4)-Br(1) bond length. The Li(4)-Cl(1) bond length is 2.65 Å. There is one shorter (2.39 Å) and one longer (2.40 Å) Li(4)-Cl(2) bond length. In the fifth Li^{1+} site, $Li(5)^{1+}$ is bonded to one $Br(2)^{1-}$, two equivalent $Br(1)^{1-}$, and two equivalent $Br(3)^{1-}$ atoms to form $LiBr_5$ trigonal bipyramids that share corners with two equivalent $Li(3)Br_4Cl$ square pyramids, corners with two equivalent $Li(6)Br_4Cl$ square pyramids, corners with two equivalent $Al(2)BrClO_3$ trigonal bipyramids, an edge with one $Li(6)Br_4Cl$ square pyramid, and edges with two equivalent $Li(5)Br_5$ trigonal bipyramids. The Li(5)-Br(2) bond length is 2.54 Å. There is one shorter (2.65 Å) and one longer (2.69 Å) Li(5)-Br(1) bond length. There is one shorter (2.58 Å) and one longer (2.59 Å) Li(5)-Br(3) bond length. In the sixth Li^{1+} site, $Li(6)^{1+}$ is bonded to two equivalent $Br(1)^{1-}$, two equivalent $Br(4)^{1-}$, and one $Cl(1)^{1-}$ atom to form $LiBr_4Cl$ square pyramids that share corners with two equivalent $Li(3)Br_4Cl$ square pyramids, corners with two equivalent $Li(5)Br_5$ trigonal bipyramids, edges with two equivalent $Li(6)Br_4Cl$ square pyramids, an edge with one $Li(5)Br_5$ trigonal bipyramid, and a faceface with one $Li(3)Br_4Cl$ square pyramid. There is one shorter (2.61 Å) and one longer (2.62 Å) Li(6)-Br(1) bond length. There is one shorter (2.52 Å) and one longer (2.57 Å) Li(6)-Br(4) bond length. The Li(6)-Cl(1) bond length is 2.50 Å. There are two inequivalent Al^{3+} sites. In the first Al^{3+} site, $Al(1)^{3+}$ is bonded in a 5-coordinate geometry to one $S(1)^{2-}$, one $O(1)^{2-}$, two equivalent $O(2)^{2-}$, and one $Cl(1)^{1-}$ atom. The Al(1)-S(1) bond length is 2.21 Å. The Al(1)-O(1) bond length is 1.87 Å. Both Al(1)-O(2) bond lengths are 1.83 Å. The Al(1)-Cl(1) bond length is 2.61 Å. In the second Al^{3+} site, $Al(2)^{3+}$ is bonded to one $O(2)^{2-}$, two equivalent $O(1)^{2-}$, one $Br(3)^{1-}$, and one $Cl(2)^{1-}$ atom to form distorted $AlBrClO_3$ trigonal bipyramids that share corners with two equivalent $Li(5)Br_5$ trigonal bipyramids and corners with two equivalent $Al(2)BrClO_3$ trigonal bipyramids. The Al(2)-O(2) bond length is 1.82 Å. Both Al(2)-O(1) bond lengths are 1.86 Å. The Al(2)-Br(3) bond length is 2.51 Å. The Al(2)-Cl(2) bond length is 2.24 Å. $S(1)^{2-}$ is bonded in a 5-coordinate geometry to two equivalent $Li(1)^{1+}$, two equivalent $Li(2)^{1+}$, and one $Al(1)^{3+}$ atom. There are two inequivalent O^{2-} sites. In the first O^{2-} site, $O(1)^{2-}$ is bonded in a T-shaped geometry to one $Al(1)^{3+}$ and two equivalent $Al(2)^{3+}$ atoms. In the second O^{2-} site, $O(2)^{2-}$ is bonded in a distorted square co-planar geometry to one $Li(1)^{1+}$, one $Al(2)^{3+}$, and two equivalent $Al(1)^{3+}$ atoms. There are four inequivalent Br^{1-} sites. In the first Br^{1-} site, $Br(1)^{1-}$ is bonded in a distorted pentagonal pyramidal geometry to two equivalent $Li(4)^{1+}$, two equivalent $Li(5)^{1+}$, and two equivalent $Li(6)^{1+}$ atoms. In the second Br^{1-} site, $Br(2)^{1-}$ is bonded in a 5-coordinate geometry to one $Li(1)^{1+}$, one $Li(4)^{1+}$, one $Li(5)^{1+}$, and two equivalent $Li(3)^{1+}$ atoms. In the third Br^{1-} site, $Br(3)^{1-}$ is bonded in a 5-coordinate geometry to two equivalent $Li(2)^{1+}$, two equivalent $Li(5)^{1+}$, and one $Al(2)^{3+}$ atom. In the fourth Br^{1-} site, $Br(4)^{1-}$ is bonded in a 5-coordinate geometry to one $Li(2)^{1+}$, two equivalent $Li(3)^{1+}$, and two equivalent $Li(6)^{1+}$ atoms.

There are two inequivalent Cl^{1-} sites. In the first Cl^{1-} site, $Cl(1)^{1-}$ is bonded in a 6-coordinate geometry to one $Li(3)^{1+}$, one $Li(4)^{1+}$, one $Li(6)^{1+}$, two equivalent $Li(1)^{1+}$, and one $Al(1)^{3+}$ atom. In the second Cl^{1-} site, $Cl(2)^{1-}$ is bonded in a trigonal non-coplanar geometry to two equivalent $Li(4)^{1+}$ and one $Al(2)^{3+}$ atom.

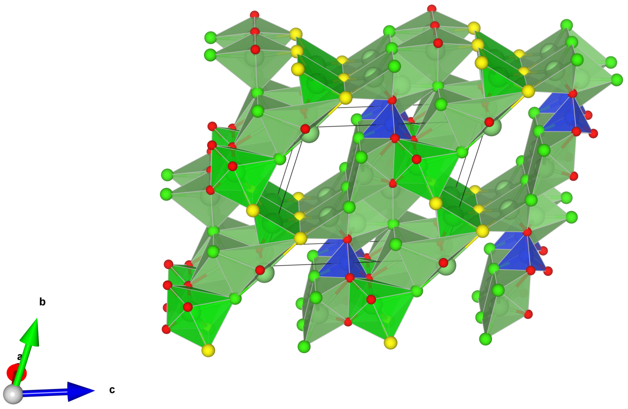

Figure S10: The predicted structure of $Li_6Zr_2SiS_2O_5Cl_4$.

10. $\mathbf{Li_6Zr_2SiS_2O_5Cl_4}$ crystallizes in the triclinic P1 space group. There are six inequivalent Li^{1+} sites. In the first Li^{1+} site, $Li(1)^{1+}$ is bonded in a 2-coordinate geometry to two equivalent $O(1)^{2-}$ and one $Cl(2)^{1-}$ atom. Both Li(1)-O(1) bond lengths are 2.00 Å. The Li(1)-Cl(2) bond length is 2.44 Å. In the second Li^{1+} site, $Li(2)^{1+}$ is bonded in a 5-coordinate geometry to one $O(3)^{2-}$, two equivalent $Cl(3)^{1-}$, and two equivalent $Cl(4)^{1-}$ atoms. The Li(2)-O(3) bond length is 1.89 Å. There is one shorter (2.47 Å) and one longer (2.48 Å) Li(2)-Cl(3) bond length. Both Li(2)-Cl(4) bond lengths are 2.53 Å. In the third Li^{1+} site, $Li(3)^{1+}$ is bonded in a 5-coordinate geometry to one $O(4)^{2-}$, two equivalent $O(2)^{2-}$, one $Cl(1)^{1-}$, and one $Cl(3)^{1-}$ atom. The Li(3)-O(4) bond length is 2.30 Å. Both Li(3)-O(2) bond lengths are 1.93 Å. The Li(3)-Cl(1) bond length is 2.29 Å. The Li(3)-Cl(3) bond length is 2.43 Å. In the fourth Li^{1+} site, $Li(4)^{1+}$ is bonded to one $S(2)^{2-}$, two equivalent $O(3)^{2-}$, one $Cl(1)^{1-}$, and one $Cl(3)^{1-}$ atom to form distorted $LiSCl_2O_2$ trigonal bipyramids that share corners with two equivalent $Li(6)Cl_4O$ square pyramids and corners with two equivalent $Li(4)SCl_2O_2$ trigonal bipyramids. The Li(4)-S(2) bond length is 2.63 Å. There is one shorter (2.06 Å) and one longer (2.07 Å) Li(4)-O(3) bond length. The Li(4)-Cl(1) bond length is 2.32 Å. The Li(4)-Cl(3) bond length is 2.68 Å. In the fifth Li^{1+} site, $Li(5)^{1+}$ is bonded in a 5-coordinate geometry to two equivalent $S(1)^{2-}$, one $O(2)^{2-}$, and two equivalent $Cl(3)^{1-}$ atoms. There is one shorter (2.59 Å) and one longer (2.65 Å) Li(5)-S(1) bond length. The Li(5)-O(2) bond length is 1.85 Å. There is one shorter (2.47 Å) and one longer (2.50 Å) Li(5)-Cl(3) bond length. In the sixth Li^{1+} site, $Li(6)^{1+}$ is bonded to one $O(1)^{2-}$, two equivalent $Cl(1)^{1-}$, and two equivalent $Cl(2)^{1-}$ atoms to form distorted $LiCl_4O$ square pyramids that share corners with two equivalent $Li(4)SCl_2O_2$ trigonal bipyramids and edges with two equivalent $Li(6)Cl_4O$ square pyramids. The Li(6)-O(1) bond length is 1.99 Å. Both Li(6)-Cl(1) bond lengths are 2.40 Å. There is one shorter (2.55 Å) and one longer (2.56 Å) Li(6)-Cl(2) bond length. There are two inequivalent Zr^{4+} sites. In the first Zr^{4+} site, $Zr(1)^{4+}$ is bonded in a 5-coordinate geometry to two equivalent $S(1)^{2-}$, two equivalent $S(2)^{2-}$, and one $O(1)^{2-}$ atom. There is one shorter (2.49 Å) and one longer (2.50 Å) Zr(1)-S(1) bond length. There is one shorter (2.61 Å) and one longer (2.62 Å) Zr(1)-S(2) bond length. The Zr(1)-O(1) bond length is 1.88 Å. In the second Zr^{4+} site, $Zr(2)^{4+}$ is bonded in a 7-coordinate geometry to two equivalent $S(2)^{2-}$, one $O(3)^{2-}$, one $O(4)^{2-}$, two equivalent $O(5)^{2-}$, and one $Cl(2)^{1-}$ atom. There is one shorter (2.77 Å) and one longer (2.79 Å) Zr(2)-S(2) bond length. The Zr(2)-O(3) bond length is 1.89 Å. The Zr(2)-O(4) bond length is 2.23 Å. Both Zr(2)-O(5) bond lengths are 2.06 Å. The Zr(2)-Cl(2) bond length is 2.85 Å. $Si(1)^{4+}$ is bonded to one $O(2)^{2-}$, one $O(5)^{2-}$,

two equivalent $O(4)^{2-}$, and one $Cl(4)^{1-}$ atom to form distorted corner-sharing $SiClO_4$ trigonal bipyramids. The Si(1)-O(2) bond length is 1.59 Å. The Si(1)-O(5) bond length is 1.72 Å. There is one shorter (1.81 Å) and one longer (1.83 Å) Si(1)-O(4) bond length. The Si(1)-Cl(4) bond length is 2.17 Å. There are two inequivalent S^{2-} sites. In the first S^{2-} site, $S(1)^{2-}$ is bonded in a 4-coordinate geometry to two equivalent $Li(5)^{1+}$ and two equivalent $Zr(1)^{4+}$ atoms. In the second S^{2-} site, $S(2)^{2-}$ is bonded in a 5-coordinate geometry to one $Li(4)^{1+}$, two equivalent $Zr(1)^{4+}$, and two equivalent $Zr(2)^{4+}$ atoms. There are five inequivalent O^{2-} sites. In the first O^{2-} site, $O(1)^{2-}$ is bonded in a trigonal pyramidal geometry to one $Li(6)^{1+}$, two equivalent $Li(1)^{1+}$, and one $Zr(1)^{4+}$ atom. In the second O^{2-} site, $O(2)^{2-}$ is bonded in a distorted trigonal pyramidal geometry to one $Li(5)^{1+}$, two equivalent $Li(3)^{1+}$, and one $Si(1)^{4+}$ atom. In the third O^{2-} site, $O(3)^{2-}$ is bonded in a tetrahedral geometry to one $Li(2)^{1+}$, two equivalent $Li(4)^{1+}$, and one $Zr(2)^{4+}$ atom. In the fourth O^{2-} site, $O(4)^{2-}$ is bonded in a 4-coordinate geometry to one $Li(3)^{1+}$, one $Zr(2)^{4+}$, and two equivalent $Si(1)^{4+}$ atoms. In the fifth O^{2-} site, $O(5)^{2-}$ is bonded in a trigonal non-coplanar geometry to two equivalent $Zr(2)^{4+}$ and one $Si(1)^{4+}$ atom. There are four inequivalent Cl^{1-} sites. In the first Cl^{1-} site, $Cl(1)^{1-}$ is bonded in a distorted tetrahedral geometry to one $Li(3)^{1+}$, one $Li(4)^{1+}$, and two equivalent $Li(6)^{1+}$ atoms. In the second Cl^{1-} site, $Cl(2)^{1-}$ is bonded in a distorted rectangular see-saw-like geometry to one $Li(1)^{1+}$, two equivalent $Li(6)^{1+}$, and one $Zr(2)^{4+}$ atom. In the third Cl^{1-} site, $Cl(3)^{1-}$ is bonded in a distorted octahedral geometry to one $Li(3)^{1+}$, one $Li(4)^{1+}$, two equivalent $Li(2)^{1+}$, and two equivalent $Li(5)^{1+}$ atoms. In the fourth Cl^{1-} site, $Cl(4)^{1-}$ is bonded in a distorted trigonal non-coplanar geometry to two equivalent $Li(2)^{1+}$ and one $Si(1)^{4+}$ atom.

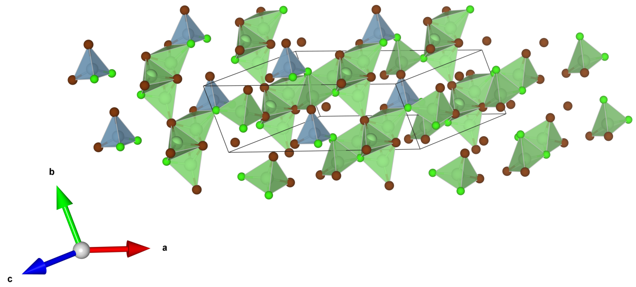

Figure S11: The predicted structure of $Li_5AlBr_5Cl_3$.

11. **$Li_5AlBr_5Cl_3$** crystallizes in the triclinic P1 space group. There are five inequivalent Li^{1+} sites. In the first Li^{1+} site, $Li(1)^{1+}$ is bonded to one $Br(1)^{1-}$, one $Br(4)^{1-}$, one $Cl(1)^{1-}$, and one $Cl(3)^{1-}$ atom to form distorted $LiBr_2Cl_2$ tetrahedra that share a corner with one $Li(2)Br_2Cl_2$ tetrahedra, a corner with one $Li(4)Br_3Cl$ tetrahedra, a corner with one $Al(1)Br_2Cl_2$ tetrahedra, a corner with one $Li(5)Br_4Cl$ trigonal bipyramid, an edge with one $Li(4)Br_3Cl$ tetrahedra, and an edge with one $Li(5)Br_4Cl$ trigonal bipyramid. The Li(1)-Br(1) bond length is 2.52 Å. The Li(1)-Br(4) bond length is 2.50 Å. The Li(1)-Cl(1) bond length is 2.44 Å. The Li(1)-Cl(3) bond length is 2.35 Å. In the second Li^{1+} site, $Li(2)^{1+}$ is bonded to one $Br(2)^{1-}$, one $Br(3)^{1-}$, one $Cl(2)^{1-}$, and one $Cl(3)^{1-}$ atom to form $LiBr_2Cl_2$ tetrahedra that share a corner with one $Li(1)Br_2Cl_2$ tetrahedra, corners with two equivalent $Li(4)Br_3Cl$ tetrahedra, corners with two equivalent $Al(1)Br_2Cl_2$ tetrahedra, and a corner with one $Li(5)Br_4Cl$ trigonal bipyramid. The Li(2)-Br(2) bond length is 2.49 Å. The Li(2)-Br(3) bond length is 2.58 Å. The Li(2)-Cl(2) bond length is 2.53 Å. The Li(2)-Cl(3) bond length is 2.31 Å. In the third Li^{1+} site, $Li(3)^{1+}$ is bonded in a 5-coordinate geometry to one $Br(1)^{1-}$, one $Br(2)^{1-}$, one $Br(4)^{1-}$, one $Br(5)^{1-}$, and one $Cl(3)^{1-}$ atom. The Li(3)-Br(1) bond length is 2.59 Å. The Li(3)-Br(2) bond length is 2.58 Å. The Li(3)-Br(4) bond length is 2.67 Å. The Li(3)-Br(5) bond length is 3.22 Å. The Li(3)-Cl(3) bond length is 2.44 Å. In the fourth Li^{1+} site, $Li(4)^{1+}$ is bonded to one $Br(1)^{1-}$, one $Br(2)^{1-}$, one $Br(4)^{1-}$, and one $Cl(3)^{1-}$ atom to form distorted $LiBr_3Cl$ tetrahedra that share a

corner with one Li(1)Br_2Cl_2 tetrahedra, corners with two equivalent Li(2)Br_2Cl_2 tetrahedra, a corner with one Li(5)Br_4Cl trigonal bipyramid, an edge with one Li(1)Br_2Cl_2 tetrahedra, and an edge with one Li(5)Br_4Cl trigonal bipyramid. The Li(4)-Br(1) bond length is 2.56 Å. The Li(4)-Br(2) bond length is 2.60 Å. The Li(4)-Br(4) bond length is 2.46 Å. The Li(4)-Cl(3) bond length is 2.38 Å. In the fifth Li^{1+} site, $Li(5)^{1+}$ is bonded to one $Br(1)^{1-}$, one $Br(2)^{1-}$, one $Br(4)^{1-}$, one $Br(5)^{1-}$, and one $Cl(1)^{1-}$ atom to form $LiBr_4Cl$ trigonal bipyramids that share a corner with one Li(1)Br_2Cl_2 tetrahedra, a corner with one Li(2)Br_2Cl_2 tetrahedra, a corner with one Li(4)Br_3Cl tetrahedra, an edge with one Li(1)Br_2Cl_2 tetrahedra, an edge with one Li(4)Br_3Cl tetrahedra, and an edge with one Al(1)Br_2Cl_2 tetrahedra. The Li(5)-Br(1) bond length is 2.58 Å. The Li(5)-Br(2) bond length is 2.60 Å. The Li(5)-Br(4) bond length is 2.53 Å. The Li(5)-Br(5) bond length is 2.83 Å. The Li(5)-Cl(1) bond length is 2.67 Å. $Al(1)^{3+}$ is bonded to one $Br(3)^{1-}$, one $Br(5)^{1-}$, one $Cl(1)^{1-}$, and one $Cl(2)^{1-}$ atom to form $AlBr_2Cl_2$ tetrahedra that share a corner with one Li(1)Br_2Cl_2 tetrahedra, corners with two equivalent Li(2)Br_2Cl_2 tetrahedra, and an edge with one Li(5)Br_4Cl trigonal bipyramid. The Al(1)-Br(3) bond length is 2.30 Å. The Al(1)-Br(5) bond length is 2.28 Å. The Al(1)-Cl(1) bond length is 2.17 Å. The Al(1)-Cl(2) bond length is 2.14 Å. There are five inequivalent Br^{1-} sites. In the first Br^{1-} site, $Br(1)^{1-}$ is bonded in a 4-coordinate geometry to one $Li(1)^{1+}$, one $Li(3)^{1+}$, one $Li(4)^{1+}$, and one $Li(5)^{1+}$ atom. In the second Br^{1-} site, $Br(2)^{1-}$ is bonded in a distorted tetrahedral geometry to one $Li(2)^{1+}$, one $Li(3)^{1+}$, one $Li(4)^{1+}$, and one $Li(5)^{1+}$ atom. In the third Br^{1-} site, $Br(3)^{1-}$ is bonded in a water-like geometry to one $Li(2)^{1+}$ and one $Al(1)^{3+}$ atom. In the fourth Br^{1-} site, $Br(4)^{1-}$ is bonded in a distorted rectangular see-saw-like geometry to one $Li(1)^{1+}$, one $Li(3)^{1+}$, one $Li(4)^{1+}$, and one $Li(5)^{1+}$ atom. In the fifth Br^{1-} site, $Br(5)^{1-}$ is bonded in a 3-coordinate geometry to one $Li(3)^{1+}$, one $Li(5)^{1+}$, and one $Al(1)^{3+}$ atom. There are three inequivalent Cl^{1-} sites. In the first Cl^{1-} site, $Cl(1)^{1-}$ is bonded in a distorted T-shaped geometry to one $Li(1)^{1+}$, one $Li(5)^{1+}$, and one $Al(1)^{3+}$ atom. In the second Cl^{1-} site, $Cl(2)^{1-}$ is bonded in a bent 120 degrees geometry to one $Li(2)^{1+}$ and one $Al(1)^{3+}$ atom. In the third Cl^{1-} site, $Cl(3)^{1-}$ is bonded in a rectangular see-saw-like geometry to one $Li(1)^{1+}$, one $Li(2)^{1+}$, one $Li(3)^{1+}$, and one $Li(4)^{1+}$ atom.

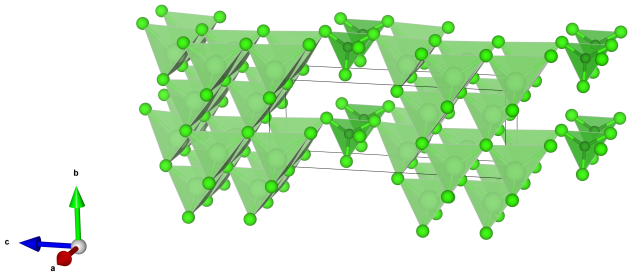

Figure S12: The predicted structure of Li_5BCl_8.

12. **Li_5BCl_8** crystallizes in the triclinic P1 space group. There are five inequivalent Li^{1+} sites. In the first Li^{1+} site, $Li(1)^{1+}$ is bonded to one $Cl(6)^{1-}$, one $Cl(7)^{1-}$, and two equivalent $Cl(1)^{1-}$ atoms to form corner-sharing $LiCl_4$ tetrahedra. The Li(1)-Cl(6) bond length is 2.79 Å. The Li(1)-Cl(7) bond length is 2.35 Å. Both Li(1)-Cl(1) bond lengths are 2.28 Å. In the second Li^{1+} site, $Li(2)^{1+}$ is bonded to one $Cl(1)^{1-}$, one $Cl(4)^{1-}$, and two equivalent $Cl(7)^{1-}$ atoms to form corner-sharing $LiCl_4$ tetrahedra. The Li(2)-Cl(1) bond length is 2.32 Å. The Li(2)-Cl(4) bond length is 2.39 Å. Both Li(2)-Cl(7) bond lengths are 2.37 Å. In the third Li^{1+} site, $Li(3)^{1+}$ is bonded to one $Cl(7)^{1-}$, one $Cl(8)^{1-}$, and two equivalent $Cl(4)^{1-}$ atoms to form corner-sharing $LiCl_4$ tetrahedra. The Li(3)-Cl(7) bond length is 2.36 Å. The Li(3)-Cl(8) bond length is 2.37 Å. Both Li(3)-Cl(4) bond lengths are 2.36 Å. In the fourth Li^{1+} site, $Li(4)^{1+}$ is bonded to one $Cl(4)^{1-}$, one $Cl(5)^{1-}$, and two equivalent $Cl(8)^{1-}$ atoms to form corner-sharing $LiCl_4$ tetrahedra. The Li(4)-Cl(4) bond length is 2.37 Å. The Li(4)-Cl(5) bond length is 2.30 Å. There is one

shorter (2.37 Å) and one longer (2.38 Å) Li(4)-Cl(8) bond length. In the fifth Li^{1+} site, $Li(5)^{1+}$ is bonded to one $Cl(3)^{1-}$, one $Cl(8)^{1-}$, and two equivalent $Cl(5)^{1-}$ atoms to form corner-sharing $LiCl_4$ tetrahedra. The Li(5)-Cl(3) bond length is 2.66 Å. The Li(5)-Cl(8) bond length is 2.33 Å. Both Li(5)-Cl(5) bond lengths are 2.29 Å. $B(1)^{3+}$ is bonded in a trigonal planar geometry to one $Cl(2)^{1-}$, one $Cl(3)^{1-}$, and one $Cl(6)^{1-}$ atom. The B(1)-Cl(2) bond length is 1.72 Å. The B(1)-Cl(3) bond length is 1.74 Å. The B(1)-Cl(6) bond length is 1.74 Å. There are eight inequivalent Cl^{1-} sites. In the first Cl^{1-} site, $Cl(1)^{1-}$ is bonded in a trigonal non-coplanar geometry to one $Li(2)^{1+}$ and two equivalent $Li(1)^{1+}$ atoms. In the second Cl^{1-} site, $Cl(2)^{1-}$ is bonded in a single-bond geometry to one $B(1)^{3+}$ atom. In the third Cl^{1-} site, $Cl(3)^{1-}$ is bonded in a distorted water-like geometry to one $Li(5)^{1+}$ and one $B(1)^{3+}$ atom. In the fourth Cl^{1-} site, $Cl(4)^{1-}$ is bonded in a tetrahedral geometry to one $Li(2)^{1+}$, one $Li(4)^{1+}$, and two equivalent $Li(3)^{1+}$ atoms. In the fifth Cl^{1-} site, $Cl(5)^{1-}$ is bonded in a trigonal non-coplanar geometry to one $Li(4)^{1+}$ and two equivalent $Li(5)^{1+}$ atoms. In the sixth Cl^{1-} site, $Cl(6)^{1-}$ is bonded in a distorted water-like geometry to one $Li(1)^{1+}$ and one $B(1)^{3+}$ atom. In the seventh Cl^{1-} site, $Cl(7)^{1-}$ is bonded in a tetrahedral geometry to one $Li(1)^{1+}$, one $Li(3)^{1+}$, and two equivalent $Li(2)^{1+}$ atoms. In the eighth Cl^{1-} site, $Cl(8)^{1-}$ is bonded in a tetrahedral geometry to one $Li(3)^{1+}$, one $Li(5)^{1+}$, and two equivalent $Li(4)^{1+}$ atoms.

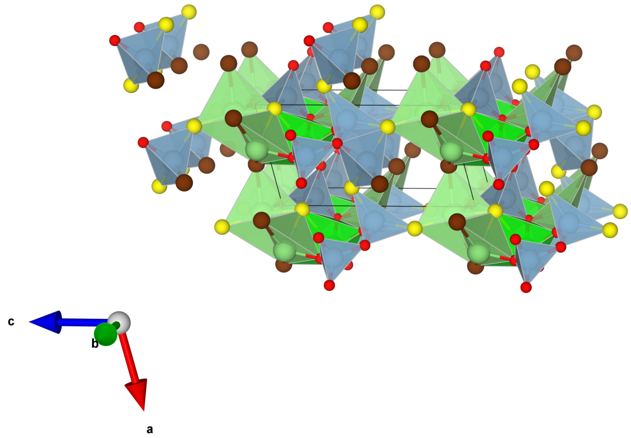

Figure S13: The predicted structure of $Li_3ZrAl_4S_3O_5Br_3$.

13. **$Li_3ZrAl_4S_3O_5Br_3$** crystallizes in the triclinic P1 space group. There are three inequivalent Li^{1+} sites. In the first Li^{1+} site, $Li(1)^{1+}$ is bonded to one $S(2)^{2-}$, one $O(5)^{2-}$, one $Br(1)^{1-}$, and one $Br(3)^{1-}$ atom to form distorted $LiSBr_2O$ trigonal pyramids that share a corner with one $Al(4)O_4$ tetrahedra, corners with two equivalent $Al(1)SBrO_2$ tetrahedra, corners with two equivalent $Li(3)S_2Br_3$ trigonal bipyramids, and an edge with one $Li(3)S_2Br_3$ trigonal bipyramid. The Li(1)-S(2) bond length is 2.52 Å. The Li(1)-O(5) bond length is 1.95 Å. The Li(1)-Br(1) bond length is 2.65 Å. The Li(1)-Br(3) bond length is 2.59 Å. In the second Li^{1+} site, $Li(2)^{1+}$ is bonded in a 4-coordinate geometry to one $O(1)^{2-}$, one $O(2)^{2-}$, one $O(3)^{2-}$, and one $Br(2)^{1-}$ atom. The Li(2)-O(1) bond length is 2.06 Å. The Li(2)-O(2) bond length is 2.00 Å. The Li(2)-O(3) bond length is 2.16 Å. The Li(2)-Br(2) bond length is 2.70 Å. In the third Li^{1+} site, $Li(3)^{1+}$ is bonded to one $S(2)^{2-}$, one $S(3)^{2-}$, one $Br(1)^{1-}$, and two equivalent $Br(3)^{1-}$ atoms to form distorted LiS_2Br_3 trigonal bipyramids that share a corner with one $Al(2)S_2BrO$ tetrahedra, a corner with one $Al(3)S_2O_2$ tetrahedra, corners with two equivalent $Li(3)S_2Br_3$ trigonal bipyramids, corners with two equivalent $Li(1)SBr_2O$ trigonal pyramids, an edge with one $Al(1)SBrO_2$ tetrahedra, and an edge with one $Li(1)SBr_2O$ trigonal pyramid. The Li(3)-S(2) bond length is 2.50 Å. The Li(3)-S(3) bond length is 2.38 Å. The Li(3)-Br(1) bond length is 2.60 Å. There is one shorter (2.70 Å) and one longer (2.96 Å) Li(3)-Br(3) bond length. $Zr(1)^{4+}$ is bonded in a 6-coordinate geometry to one $S(2)^{2-}$, one $O(1)^{2-}$, one $O(2)^{2-}$, one $O(4)^{2-}$, one

$O(5)^{2-}$, and one $Br(3)^{1-}$ atom. The Zr(1)-S(2) bond length is 2.67 Å. The Zr(1)-O(1) bond length is 2.34 Å. The Zr(1)-O(2) bond length is 1.95 Å. The Zr(1)-O(4) bond length is 2.25 Å. The Zr(1)-O(5) bond length is 1.99 Å. The Zr(1)-Br(3) bond length is 2.91 Å. There are four inequivalent Al^{3+} sites. In the first Al^{3+} site, $Al(1)^{3+}$ is bonded to one $S(2)^{2-}$, one $O(1)^{2-}$, one $O(4)^{2-}$, and one $Br(1)^{1-}$ atom to form $AlSBrO_2$ tetrahedra that share corners with two equivalent $Al(4)O_4$ tetrahedra, corners with two equivalent $Li(1)SBr_2O$ trigonal pyramids, and an edge with one $Li(3)S_2Br_3$ trigonal bipyramid. The Al(1)-S(2) bond length is 2.18 Å. The Al(1)-O(1) bond length is 1.74 Å. The Al(1)-O(4) bond length is 1.75 Å. The Al(1)-Br(1) bond length is 2.36 Å. In the second Al^{3+} site, $Al(2)^{3+}$ is bonded to one $S(1)^{2-}$, one $S(3)^{2-}$, one $O(3)^{2-}$, and one $Br(2)^{1-}$ atom to form distorted AlS_2BrO tetrahedra that share a corner with one $Al(4)O_4$ tetrahedra, a corner with one $Al(3)S_2O_2$ tetrahedra, a corner with one $Li(3)S_2Br_3$ trigonal bipyramid, and an edge with one $Al(3)S_2O_2$ tetrahedra. The Al(2)-S(1) bond length is 2.18 Å. The Al(2)-S(3) bond length is 2.22 Å. The Al(2)-O(3) bond length is 1.84 Å. The Al(2)-Br(2) bond length is 2.31 Å. In the third Al^{3+} site, $Al(3)^{3+}$ is bonded to one $S(1)^{2-}$, one $S(3)^{2-}$, one $O(2)^{2-}$, and one $O(3)^{2-}$ atom to form distorted AlS_2O_2 tetrahedra that share a corner with one $Al(4)O_4$ tetrahedra, a corner with one $Al(2)S_2BrO$ tetrahedra, a corner with one $Li(3)S_2Br_3$ trigonal bipyramid, and an edge with one $Al(2)S_2BrO$ tetrahedra. The Al(3)-S(1) bond length is 2.17 Å. The Al(3)-S(3) bond length is 2.23 Å. The Al(3)-O(2) bond length is 1.77 Å. The Al(3)-O(3) bond length is 1.86 Å. In the fourth Al^{3+} site, $Al(4)^{3+}$ is bonded to one $O(1)^{2-}$, one $O(3)^{2-}$, one $O(4)^{2-}$, and one $O(5)^{2-}$ atom to form AlO_4 tetrahedra that share a corner with one $Al(2)S_2BrO$ tetrahedra, a corner with one $Al(3)S_2O_2$ tetrahedra, corners with two equivalent $Al(1)SBrO_2$ tetrahedra, and a corner with one $Li(1)SBr_2O$ trigonal pyramid. The Al(4)-O(1) bond length is 1.77 Å. The Al(4)-O(3) bond length is 1.78 Å. The Al(4)-O(4) bond length is 1.76 Å. The Al(4)-O(5) bond length is 1.72 Å. There are three inequivalent S^{2-} sites. In the first S^{2-} site, $S(1)^{2-}$ is bonded in a bent 120 degrees geometry to one $Al(2)^{3+}$ and one $Al(3)^{3+}$ atom. In the second S^{2-} site, $S(2)^{2-}$ is bonded in a 4-coordinate geometry to one $Li(1)^{1+}$, one $Li(3)^{1+}$, one $Zr(1)^{4+}$, and one $Al(1)^{3+}$ atom. In the third S^{2-} site, $S(3)^{2-}$ is bonded in a 3-coordinate geometry to one $Li(3)^{1+}$, one $Al(2)^{3+}$, and one $Al(3)^{3+}$ atom. There are five inequivalent O^{2-} sites. In the first O^{2-} site, $O(1)^{2-}$ is bonded in a rectangular see-saw-like geometry to one $Li(2)^{1+}$, one $Zr(1)^{4+}$, one $Al(1)^{3+}$, and one $Al(4)^{3+}$ atom. In the second O^{2-} site, $O(2)^{2-}$ is bonded in a distorted trigonal planar geometry to one $Li(2)^{1+}$, one $Zr(1)^{4+}$, and one $Al(3)^{3+}$ atom. In the third O^{2-} site, $O(3)^{2-}$ is bonded in a distorted tetrahedral geometry to one $Li(2)^{1+}$, one $Al(2)^{3+}$, one $Al(3)^{3+}$, and one $Al(4)^{3+}$ atom. In the fourth O^{2-} site, $O(4)^{2-}$ is bonded in a distorted trigonal planar geometry to one $Zr(1)^{4+}$, one $Al(1)^{3+}$, and one $Al(4)^{3+}$ atom. In the fifth O^{2-} site, $O(5)^{2-}$ is bonded in a distorted trigonal planar geometry to one $Li(1)^{1+}$, one $Zr(1)^{4+}$, and one $Al(4)^{3+}$ atom. There are three inequivalent Br^{1-} sites. In the first Br^{1-} site, $Br(1)^{1-}$ is bonded in a 3-coordinate geometry to one $Li(1)^{1+}$, one $Li(3)^{1+}$, and one $Al(1)^{3+}$ atom. In the second Br^{1-} site, $Br(2)^{1-}$ is bonded in an L-shaped geometry to one $Li(2)^{1+}$ and one $Al(2)^{3+}$ atom. In the third Br^{1-} site, $Br(3)^{1-}$ is bonded in a 4-coordinate geometry to one $Li(1)^{1+}$, two equivalent $Li(3)^{1+}$, and one $Zr(1)^{4+}$ atom.

14. $\mathbf{Li_4Zr_2Si(O_3Cl_2)_2}$ crystallizes in the triclinic P1 space group. There are four inequivalent Li^{1+} sites. In the first Li^{1+} site, $Li(1)^{1+}$ is bonded in a 6-coordinate geometry to one $O(3)^{2-}$, one $O(5)^{2-}$, one $Cl(1)^{1-}$, one $Cl(2)^{1-}$, one $Cl(3)^{1-}$, and one $Cl(4)^{1-}$ atom. The Li(1)-O(3) bond length is 2.06 Å. The Li(1)-O(5) bond length is 2.02 Å. The Li(1)-Cl(1) bond length is 3.15 Å. The Li(1)-Cl(2) bond length is 2.38 Å. The Li(1)-Cl(3) bond length is 2.59 Å. The Li(1)-Cl(4) bond length is 2.74 Å. In the second Li^{1+} site, $Li(2)^{1+}$ is bonded in a 4-coordinate geometry to one $O(2)^{2-}$, one $O(6)^{2-}$, one $Cl(1)^{1-}$, and one $Cl(4)^{1-}$ atom. The Li(2)-O(2) bond length is 1.98 Å. The Li(2)-O(6) bond length is 2.01 Å. The Li(2)-Cl(1) bond length is 2.40 Å. The Li(2)-Cl(4) bond length is 2.62 Å. In the third Li^{1+} site, $Li(3)^{1+}$ is bonded in a 4-coordinate geometry to one $O(1)^{2-}$, one $O(3)^{2-}$, one $O(4)^{2-}$, one $O(5)^{2-}$, and one $Cl(1)^{1-}$ atom. The Li(3)-O(1) bond length is 2.02 Å. The Li(3)-O(3) bond length is 1.92 Å. The Li(3)-O(4) bond

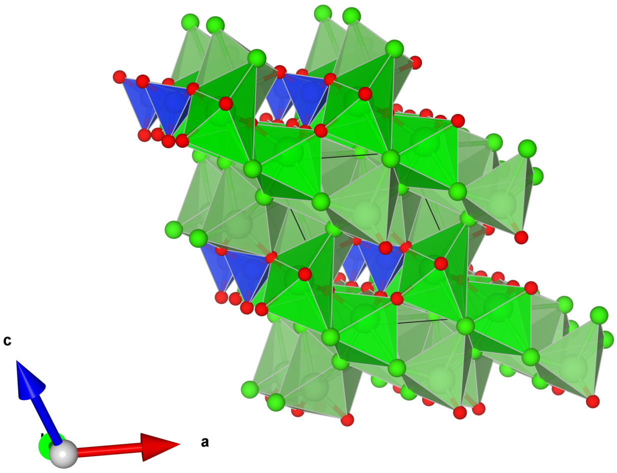

Figure S14: The predicted structure of $Li_4Zr_2Si(O_3Cl_2)_2$.

length is 2.06 Å. The Li(3)-O(5) bond length is 2.42 Å. The Li(3)-Cl(1) bond length is 2.92 Å. In the fourth Li^{1+} site, $Li(4)^{1+}$ is bonded in a 4-coordinate geometry to one $O(3)^{2-}$, one $Cl(1)^{1-}$, one $Cl(2)^{1-}$, and one $Cl(3)^{1-}$ atom. The Li(4)-O(3) bond length is 1.85 Å. The Li(4)-Cl(1) bond length is 2.46 Å. The Li(4)-Cl(2) bond length is 2.33 Å. The Li(4)-Cl(3) bond length is 2.37 Å. There are two inequivalent Zr^{4+} sites. In the first Zr^{4+} site, $Zr(1)^{4+}$ is bonded in a 6-coordinate geometry to one $O(1)^{2-}$, one $O(2)^{2-}$, one $O(6)^{2-}$, one $Cl(1)^{1-}$, one $Cl(3)^{1-}$, and one $Cl(4)^{1-}$ atom. The Zr(1)-O(1) bond length is 1.96 Å. The Zr(1)-O(2) bond length is 2.03 Å. The Zr(1)-O(6) bond length is 1.99 Å. The Zr(1)-Cl(1) bond length is 2.65 Å. The Zr(1)-Cl(3) bond length is 2.54 Å. The Zr(1)-Cl(4) bond length is 2.73 Å. In the second Zr^{4+} site, $Zr(2)^{4+}$ is bonded in a 4-coordinate geometry to one $O(1)^{2-}$, one $O(4)^{2-}$, one $O(5)^{2-}$, one $O(6)^{2-}$, one $Cl(2)^{1-}$, and one $Cl(4)^{1-}$ atom. The Zr(2)-O(1) bond length is 2.00 Å. The Zr(2)-O(4) bond length is 2.05 Å. The Zr(2)-O(5) bond length is 2.03 Å. The Zr(2)-O(6) bond length is 2.01 Å. The Zr(2)-Cl(2) bond length is 2.67 Å. The Zr(2)-Cl(4) bond length is 2.81 Å. $Si(1)^{4+}$ is bonded in a tetrahedral geometry to one $O(2)^{2-}$, one $O(3)^{2-}$, one $O(4)^{2-}$, and one $O(5)^{2-}$ atom. The Si(1)-O(2) bond length is 1.66 Å. The Si(1)-O(3) bond length is 1.60 Å. The Si(1)-O(4) bond length is 1.63 Å. The Si(1)-O(5) bond length is 1.65 Å. There are six inequivalent O^{2-} sites. In the first O^{2-} site, $O(1)^{2-}$ is bonded in a distorted trigonal planar geometry to one $Li(3)^{1+}$, one $Zr(1)^{4+}$, and one $Zr(2)^{4+}$ atom. In the second O^{2-} site, $O(2)^{2-}$ is bonded in a trigonal planar geometry to one $Li(2)^{1+}$, one $Zr(1)^{4+}$, and one $Si(1)^{4+}$ atom. In the third O^{2-} site, $O(3)^{2-}$ is bonded in a distorted trigonal pyramidal geometry to one $Li(1)^{1+}$, one $Li(3)^{1+}$, one $Li(4)^{1+}$, and one $Si(1)^{4+}$ atom. In the fourth O^{2-} site, $O(4)^{2-}$ is bonded in a 3-coordinate geometry to one $Li(3)^{1+}$, one $Zr(2)^{4+}$, and one $Si(1)^{4+}$ atom. In the fifth O^{2-} site, $O(5)^{2-}$ is bonded in a 4-coordinate geometry to one $Li(1)^{1+}$, one $Li(3)^{1+}$, one $Zr(2)^{4+}$, and one $Si(1)^{4+}$ atom. In the sixth O^{2-} site, $O(6)^{2-}$ is bonded in a trigonal non-coplanar geometry to one $Li(2)^{1+}$, one $Zr(1)^{4+}$, and one $Zr(2)^{4+}$ atom. There are four inequivalent Cl^{1-} sites. In the first Cl^{1-} site, $Cl(1)^{1-}$ is bonded in a 5-coordinate geometry to one $Li(1)^{1+}$, one $Li(2)^{1+}$, one $Li(3)^{1+}$, one $Li(4)^{1+}$, and one $Zr(1)^{4+}$ atom. In the second Cl^{1-} site, $Cl(2)^{1-}$ is bonded in a 3-coordinate geometry to one $Li(1)^{1+}$, one $Li(4)^{1+}$, and one $Zr(2)^{4+}$ atom. In the third Cl^{1-} site, $Cl(3)^{1-}$ is bonded in a 3-coordinate geometry to one $Li(1)^{1+}$, one $Li(4)^{1+}$, and one $Zr(1)^{4+}$ atom. In the fourth Cl^{1-} site, $Cl(4)^{1-}$ is bonded in a 4-coordinate geometry to one $Li(1)^{1+}$, one $Li(2)^{1+}$, one $Zr(1)^{4+}$, and one $Zr(2)^{4+}$ atom.

15. $\mathbf{Li_2Al_3SiS_2Br_5Cl_6}$ crystallizes in the triclinic P1 space group. The structure is two-dimensional and consists of one $Li_2Al_3SiS_2Br_5Cl_6$ sheet oriented in the (0, 0, 1) direction. There are two

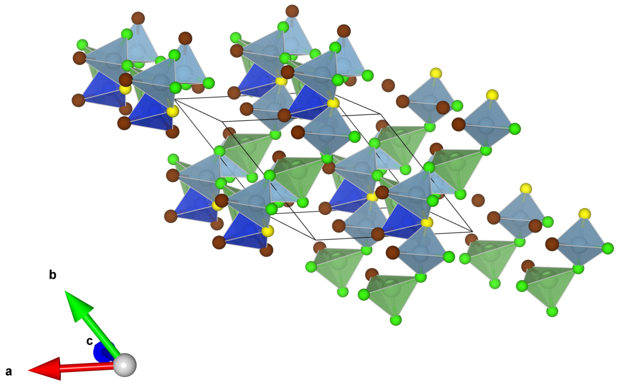

Figure S15: The predicted structure of $Li_2Al_3SiS_2Br_5Cl_6$.

inequivalent Li^{1+} sites. In the first Li^{1+} site, $Li(1)^{1+}$ is bonded to one $Br(3)^{1-}$, one $Cl(1)^{1-}$, one $Cl(3)^{1-}$, and one $Cl(5)^{1-}$ atom to form $LiBrCl_3$ tetrahedra that share a corner with one $Al(2)SBrCl_2$ tetrahedra, a corner with one $Al(3)SBrCl_2$ tetrahedra, and an edge with one $Al(1)SBrCl_2$ tetrahedra. The Li(1)-Br(3) bond length is 2.67 Å. The Li(1)-Cl(1) bond length is 2.44 Å. The Li(1)-Cl(3) bond length is 2.37 Å. The Li(1)-Cl(5) bond length is 2.37 Å. In the second Li^{1+} site, $Li(2)^{1+}$ is bonded to one $S(1)^{2-}$, one $Cl(2)^{1-}$, one $Cl(4)^{1-}$, and one $Cl(6)^{1-}$ atom to form distorted $LiSCl_3$ tetrahedra that share a corner with one $Al(2)SBrCl_2$ tetrahedra, a corner with one $Al(3)SBrCl_2$ tetrahedra, a corner with one $Si(1)S_2Br_2$ tetrahedra, and an edge with one $Al(1)SBrCl_2$ tetrahedra. The Li(2)-S(1) bond length is 2.61 Å. The Li(2)-Cl(2) bond length is 2.40 Å. The Li(2)-Cl(4) bond length is 2.35 Å. The Li(2)-Cl(6) bond length is 2.36 Å. There are three inequivalent Al^{3+} sites. In the first Al^{3+} site, $Al(1)^{3+}$ is bonded to one $S(1)^{2-}$, one $Br(3)^{1-}$, one $Cl(1)^{1-}$, and one $Cl(2)^{1-}$ atom to form $AlSBrCl_2$ tetrahedra that share a corner with one $Si(1)S_2Br_2$ tetrahedra, an edge with one $Li(1)BrCl_3$ tetrahedra, and an edge with one $Li(2)SCl_3$ tetrahedra. The Al(1)-S(1) bond length is 2.28 Å. The Al(1)-Br(3) bond length is 2.29 Å. The Al(1)-Cl(1) bond length is 2.14 Å. The Al(1)-Cl(2) bond length is 2.14 Å. In the second Al^{3+} site, $Al(2)^{3+}$ is bonded to one $S(2)^{2-}$, one $Br(4)^{1-}$, one $Cl(3)^{1-}$, and one $Cl(4)^{1-}$ atom to form $AlSBrCl_2$ tetrahedra that share a corner with one $Li(1)BrCl_3$ tetrahedra, a corner with one $Li(2)SCl_3$ tetrahedra, a corner with one $Al(3)SBrCl_2$ tetrahedra, and a corner with one $Si(1)S_2Br_2$ tetrahedra. The Al(2)-S(2) bond length is 2.35 Å. The Al(2)-Br(4) bond length is 2.24 Å. The Al(2)-Cl(3) bond length is 2.15 Å. The Al(2)-Cl(4) bond length is 2.15 Å. In the third Al^{3+} site, $Al(3)^{3+}$ is bonded to one $S(2)^{2-}$, one $Br(2)^{1-}$, one $Cl(5)^{1-}$, and one $Cl(6)^{1-}$ atom to form $AlSBrCl_2$ tetrahedra that share a corner with one $Li(1)BrCl_3$ tetrahedra, a corner with one $Li(2)SCl_3$ tetrahedra, a corner with one $Al(2)SBrCl_2$ tetrahedra, and a corner with one $Si(1)S_2Br_2$ tetrahedra. The Al(3)-S(2) bond length is 2.34 Å. The Al(3)-Br(2) bond length is 2.24 Å. The Al(3)-Cl(5) bond length is 2.15 Å. The Al(3)-Cl(6) bond length is 2.15 Å. $Si(1)^{4+}$ is bonded to one $S(1)^{2-}$, one $S(2)^{2-}$, one $Br(1)^{1-}$, and one $Br(5)^{1-}$ atom to form SiS_2Br_2 tetrahedra that share a corner with one $Li(2)SCl_3$ tetrahedra, a corner with one $Al(1)SBrCl_2$ tetrahedra, a corner with one $Al(2)SBrCl_2$ tetrahedra, and a corner with one $Al(3)SBrCl_2$ tetrahedra. The Si(1)-S(1) bond length is 2.08 Å. The Si(1)-S(2) bond length is 2.16 Å. The Si(1)-Br(1) bond length is 2.19 Å. The Si(1)-Br(5) bond length is 2.20 Å. There are two inequivalent S^{2-} sites. In the first S^{2-} site, $S(1)^{2-}$ is bonded in a 3-coordinate geometry to one $Li(2)^{1+}$, one $Al(1)^{3+}$, and one $Si(1)^{4+}$ atom. In the second S^{2-} site, $S(2)^{2-}$ is bonded in a trigonal non-coplanar geometry to one $Al(2)^{3+}$, one $Al(3)^{3+}$, and one $Si(1)^{4+}$ atom. There are five inequivalent Br^{1-} sites. In the first Br^{1-} site, $Br(1)^{1-}$ is bonded in a single-bond geometry to one $Si(1)^{4+}$ atom. In the second Br^{1-} site, $Br(2)^{1-}$ is bonded in a single-bond geometry to one $Al(3)^{3+}$ atom. In the third Br^{1-} site, $Br(3)^{1-}$ is bonded in a distorted L-shaped geometry to one $Li(1)^{1+}$ and one $Al(1)^{3+}$ atom. In the fourth Br^{1-} site, $Br(4)^{1-}$ is bonded in a single-

bond geometry to one $Al(2)^{3+}$ atom. In the fifth Br^{1-} site, $Br(5)^{1-}$ is bonded in a single-bond geometry to one $Si(1)^{4+}$ atom. There are six inequivalent Cl^{1-} sites. In the first Cl^{1-} site, $Cl(1)^{1-}$ is bonded in a distorted L-shaped geometry to one $Li(1)^{1+}$ and one $Al(1)^{3+}$ atom. In the second Cl^{1-} site, $Cl(2)^{1-}$ is bonded in an L-shaped geometry to one $Li(2)^{1+}$ and one $Al(1)^{3+}$ atom. In the third Cl^{1-} site, $Cl(3)^{1-}$ is bonded in a bent 120 degrees geometry to one $Li(1)^{1+}$ and one $Al(2)^{3+}$ atom. In the fourth Cl^{1-} site, $Cl(4)^{1-}$ is bonded in a water-like geometry to one $Li(2)^{1+}$ and one $Al(2)^{3+}$ atom. In the fifth Cl^{1-} site, $Cl(5)^{1-}$ is bonded in a distorted bent 120 degrees geometry to one $Li(1)^{1+}$ and one $Al(3)^{3+}$ atom. In the sixth Cl^{1-} site, $Cl(6)^{1-}$ is bonded in a bent 120 degrees geometry to one $Li(2)^{1+}$ and one $Al(3)^{3+}$ atom.

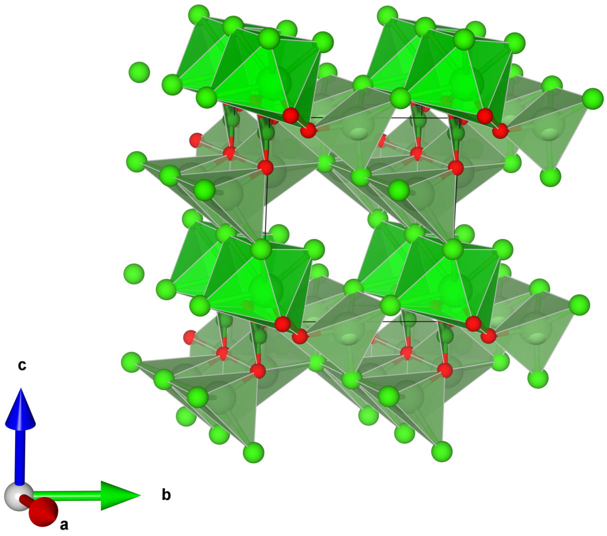

Figure S16: The predicted structure of $Li_3ZrBO_3Cl_4$.

16. **$Li_3ZrBO_3Cl_4$** crystallizes in the triclinic P1 space group. There are three inequivalent Li^{1+} sites. In the first Li^{1+} site, $Li(1)^{1+}$ is bonded to one $O(1)^{2-}$, one $Cl(1)^{1-}$, and two equivalent $Cl(3)^{1-}$ atoms to form distorted corner-sharing $LiCl_3O$ tetrahedra. The Li(1)-O(1) bond length is 1.95 Å. The Li(1)-Cl(1) bond length is 2.36 Å. There is one shorter (2.38 Å) and one longer (2.41 Å) Li(1)-Cl(3) bond length. In the second Li^{1+} site, $Li(2)^{1+}$ is bonded in a 5-coordinate geometry to one $O(1)^{2-}$, two equivalent $O(3)^{2-}$, one $Cl(1)^{1-}$, and one $Cl(2)^{1-}$ atom. The Li(2)-O(1) bond length is 2.18 Å. There is one shorter (1.94 Å) and one longer (2.39 Å) Li(2)-O(3) bond length. The Li(2)-Cl(1) bond length is 2.44 Å. The Li(2)-Cl(2) bond length is 2.82 Å. In the third Li^{1+} site, $Li(3)^{1+}$ is bonded in a 4-coordinate geometry to one $O(3)^{2-}$, one $Cl(2)^{1-}$, and two equivalent $Cl(1)^{1-}$ atoms. The Li(3)-O(3) bond length is 1.91 Å. The Li(3)-Cl(2) bond length is 2.43 Å. There is one shorter (2.28 Å) and one longer (2.82 Å) Li(3)-Cl(1) bond length. $Zr(1)^{4+}$ is bonded in a 7-coordinate geometry to one $O(1)^{2-}$, two equivalent $O(2)^{2-}$, one $Cl(3)^{1-}$, one $Cl(4)^{1-}$, and two equivalent $Cl(2)^{1-}$ atoms. The Zr(1)-O(1) bond length is 2.13 Å. There is one shorter (2.07 Å) and one longer (2.14 Å) Zr(1)-O(2) bond length. The Zr(1)-Cl(3) bond length is 2.53 Å. The Zr(1)-Cl(4) bond length is 2.36 Å. There is one shorter (2.71 Å) and one longer (2.83 Å) Zr(1)-Cl(2) bond length. $B(1)^{3+}$ is bonded in a trigonal planar geometry to one $O(1)^{2-}$, one $O(2)^{2-}$, and one $O(3)^{2-}$ atom. The B(1)-O(1) bond length is 1.39 Å. The B(1)-O(2) bond length is 1.43 Å. The B(1)-O(3) bond length is 1.31 Å. There are three inequivalent O^{2-} sites. In the first O^{2-} site, $O(1)^{2-}$ is bonded in a 4-coordinate geometry to one $Li(1)^{1+}$, one $Li(2)^{1+}$, one $Zr(1)^{4+}$, and one $B(1)^{3+}$ atom. In the second O^{2-} site, $O(2)^{2-}$ is bonded in a 3-coordinate geometry to two equivalent $Zr(1)^{4+}$ and one $B(1)^{3+}$ atom. In the third O^{2-} site, $O(3)^{2-}$ is bonded in a 4-coordinate geometry to one $Li(3)^{1+}$, two equivalent $Li(2)^{1+}$, and one $B(1)^{3+}$ atom. There are four inequivalent Cl^{1-} sites. In the first Cl^{1-} site, $Cl(1)^{1-}$

is bonded in a 4-coordinate geometry to one $Li(1)^{1+}$, one $Li(2)^{1+}$, and two equivalent $Li(3)^{1+}$ atoms. In the second Cl^{1-} site, $Cl(2)^{1-}$ is bonded in a 4-coordinate geometry to one $Li(2)^{1+}$, one $Li(3)^{1+}$, and two equivalent $Zr(1)^{4+}$ atoms. In the third Cl^{1-} site, $Cl(3)^{1-}$ is bonded in a distorted trigonal non-coplanar geometry to two equivalent $Li(1)^{1+}$ and one $Zr(1)^{4+}$ atom. In the fourth Cl^{1-} site, $Cl(4)^{1-}$ is bonded in a single-bond geometry to one $Zr(1)^{4+}$ atom.

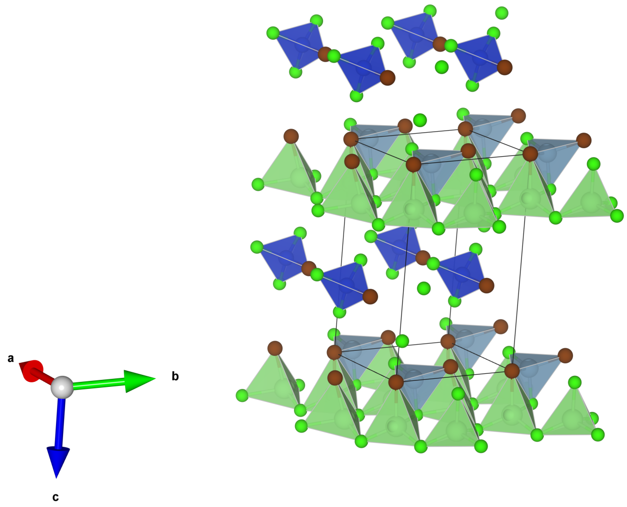

Figure S17: The predicted structure of $Li_3Al(BrCl_2)_2SiBrCl_3$.

17. **$Li_3Al(BrCl_2)_2SiBrCl_3$** crystallizes in the triclinic P1 space group. The structure is two-dimensional and consists of one bromotrichlorosilane molecule and one $Li_3Al(BrCl_2)_2$ sheet oriented in the (0, 0, 1) direction. In the $Li_3Al(BrCl_2)_2$ sheet, there are three inequivalent Li^{1+} sites. In the first Li^{1+} site, $Li(1)^{1+}$ is bonded to one $Br(2)^{1-}$, one $Cl(2)^{1-}$, one $Cl(3)^{1-}$, and one $Cl(5)^{1-}$ atom to form distorted $LiBrCl_3$ tetrahedra that share corners with three equivalent $Li(3)BrCl_3$ tetrahedra, corners with three equivalent $Li(2)Cl_4$ tetrahedra, and an edge with one $Al(1)Br_2Cl_2$ tetrahedra. The Li(1)-Br(2) bond length is 2.63 Å. The Li(1)-Cl(2) bond length is 2.56 Å. The Li(1)-Cl(3) bond length is 2.27 Å. The Li(1)-Cl(5) bond length is 2.27 Å. In the second Li^{1+} site, $Li(2)^{1+}$ is bonded to one $Cl(2)^{1-}$, one $Cl(3)^{1-}$, one $Cl(4)^{1-}$, and one $Cl(5)^{1-}$ atom to form $LiCl_4$ tetrahedra that share corners with three equivalent $Li(1)BrCl_3$ tetrahedra, corners with three equivalent $Li(3)BrCl_3$ tetrahedra, and an edge with one $Al(1)Br_2Cl_2$ tetrahedra. The Li(2)-Cl(2) bond length is 2.58 Å. The Li(2)-Cl(3) bond length is 2.26 Å. The Li(2)-Cl(4) bond length is 2.46 Å. The Li(2)-Cl(5) bond length is 2.27 Å. In the third Li^{1+} site, $Li(3)^{1+}$ is bonded to one $Br(1)^{1-}$, one $Cl(2)^{1-}$, one $Cl(3)^{1-}$, and one $Cl(5)^{1-}$ atom to form $LiBrCl_3$ tetrahedra that share corners with three equivalent $Li(1)BrCl_3$ tetrahedra, corners with three equivalent $Li(2)Cl_4$ tetrahedra, and an edge with one $Al(1)Br_2Cl_2$ tetrahedra. The Li(3)-Br(1) bond length is 2.64 Å. The Li(3)-Cl(2) bond length is 2.58 Å. The Li(3)-Cl(3) bond length is 2.26 Å. The Li(3)-Cl(5) bond length is 2.26 Å. $Al(1)^{3+}$ is bonded to one $Br(1)^{1-}$, one $Br(2)^{1-}$, one $Cl(2)^{1-}$, and one $Cl(4)^{1-}$ atom to form $AlBr_2Cl_2$ tetrahedra that share an edge with one $Li(1)BrCl_3$ tetrahedra, an edge with one $Li(3)BrCl_3$ tetrahedra, and an edge with one $Li(2)Cl_4$ tetrahedra. The Al(1)-Br(1) bond length is 2.28 Å. The Al(1)-Br(2) bond length is 2.28 Å. The Al(1)-Cl(2) bond length is 2.22 Å. The Al(1)-Cl(4) bond length is 2.13 Å. There are two inequivalent Br^{1-} sites. In the first Br^{1-} site, $Br(1)^{1-}$ is bonded in an L-shaped geometry to one $Li(3)^{1+}$ and one $Al(1)^{3+}$ atom. In the second Br^{1-} site, $Br(2)^{1-}$ is bonded in an L-shaped geometry to one $Li(1)^{1+}$ and one $Al(1)^{3+}$ atom. There are four inequivalent Cl^{1-} sites. In the first Cl^{1-} site, $Cl(2)^{1-}$ is bonded in a trigonal pyramidal geometry to one $Li(1)^{1+}$, one $Li(2)^{1+}$, one $Li(3)^{1+}$, and one $Al(1)^{3+}$ atom. In the second Cl^{1-} site, $Cl(3)^{1-}$ is bonded in a trigonal non-coplanar geometry to one $Li(1)^{1+}$, one $Li(2)^{1+}$, and one $Li(3)^{1+}$ atom. In the third

Cl^{1-} site, $Cl(4)^{1-}$ is bonded in an L-shaped geometry to one $Li(2)^{1+}$ and one $Al(1)^{3+}$ atom. In the fourth Cl^{1-} site, $Cl(5)^{1-}$ is bonded in a trigonal non-coplanar geometry to one $Li(1)^{1+}$, one $Li(2)^{1+}$, and one $Li(3)^{1+}$ atom.

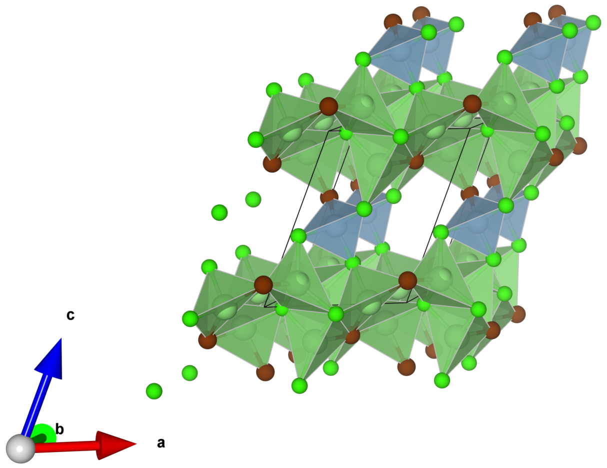

Figure S18: The predicted structure of $Li_5AlBr_3Cl_5$.

18. **$Li_5AlBr_3Cl_5$** crystallizes in the triclinic P1 space group. There are five inequivalent Li^{1+} sites. In the first Li^{1+} site, $Li(1)^{1+}$ is bonded to one $Br(2)^{1-}$, one $Cl(1)^{1-}$, one $Cl(2)^{1-}$, and one $Cl(4)^{1-}$ atom to form distorted $LiBrCl_3$ tetrahedra that share a corner with one $Al(1)BrCl_3$ tetrahedra, corners with three equivalent $Li(2)Br_2Cl_2$ tetrahedra, an edge with one $Li(3)Br_2Cl_2$ tetrahedra, an edge with one $Li(5)Br_2Cl_2$ tetrahedra, and an edge with one $Li(4)BrCl_3$ tetrahedra. The Li(1)-Br(2) bond length is 2.54 Å. The Li(1)-Cl(1) bond length is 2.36 Å. The Li(1)-Cl(2) bond length is 2.38 Å. The Li(1)-Cl(4) bond length is 2.34 Å. In the second Li^{1+} site, $Li(2)^{1+}$ is bonded to one $Br(1)^{1-}$, one $Br(2)^{1-}$, one $Cl(1)^{1-}$, and one $Cl(2)^{1-}$ atom to form $LiBr_2Cl_2$ tetrahedra that share a corner with one $Al(1)BrCl_3$ tetrahedra, corners with two equivalent $Li(3)Br_2Cl_2$ tetrahedra, corners with two equivalent $Li(5)Br_2Cl_2$ tetrahedra, corners with two equivalent $Li(4)BrCl_3$ tetrahedra, and corners with three equivalent $Li(1)BrCl_3$ tetrahedra. The Li(2)-Br(1) bond length is 2.59 Å. The Li(2)-Br(2) bond length is 2.50 Å. The Li(2)-Cl(1) bond length is 2.34 Å. The Li(2)-Cl(2) bond length is 2.34 Å. In the third Li^{1+} site, $Li(3)^{1+}$ is bonded to one $Br(2)^{1-}$, one $Br(3)^{1-}$, one $Cl(2)^{1-}$, and one $Cl(5)^{1-}$ atom to form $LiBr_2Cl_2$ tetrahedra that share a corner with one $Li(5)Br_2Cl_2$ tetrahedra, a corner with one $Al(1)BrCl_3$ tetrahedra, corners with two equivalent $Li(2)Br_2Cl_2$ tetrahedra, corners with two equivalent $Li(4)BrCl_3$ tetrahedra, an edge with one $Li(5)Br_2Cl_2$ tetrahedra, and an edge with one $Li(1)BrCl_3$ tetrahedra. The Li(3)-Br(2) bond length is 2.57 Å. The Li(3)-Br(3) bond length is 2.42 Å. The Li(3)-Cl(2) bond length is 2.37 Å. The Li(3)-Cl(5) bond length is 2.51 Å. In the fourth Li^{1+} site, $Li(4)^{1+}$ is bonded to one $Br(3)^{1-}$, one $Cl(1)^{1-}$, one $Cl(2)^{1-}$, and one $Cl(3)^{1-}$ atom to form $LiBrCl_3$ tetrahedra that share a corner with one $Al(1)BrCl_3$ tetrahedra, corners with two equivalent $Li(2)Br_2Cl_2$ tetrahedra, corners with two equivalent $Li(3)Br_2Cl_2$ tetrahedra, corners with two equivalent $Li(5)Br_2Cl_2$ tetrahedra, and an edge with one $Li(1)BrCl_3$ tetrahedra. The Li(4)-Br(3) bond length is 2.41 Å. The Li(4)-Cl(1) bond length is 2.37 Å. The Li(4)-Cl(2) bond length is 2.41 Å. The Li(4)-Cl(3) bond length is 2.41 Å. In the fifth Li^{1+} site, $Li(5)^{1+}$ is bonded to one $Br(2)^{1-}$, one $Br(3)^{1-}$, one $Cl(1)^{1-}$, and one $Cl(5)^{1-}$ atom to form $LiBr_2Cl_2$ tetrahedra that share a corner with one $Li(3)Br_2Cl_2$ tetrahedra, a corner with one $Al(1)BrCl_3$ tetrahedra, corners with two equivalent $Li(2)Br_2Cl_2$ tetrahedra, corners with two equivalent $Li(4)BrCl_3$ tetrahedra, an edge with one $Li(3)Br_2Cl_2$ tetrahedra, and an edge with one $Li(1)BrCl_3$ tetrahedra. The Li(5)-Br(2) bond length is 2.55 Å. The Li(5)-Br(3) bond length

is 2.40 Å. The Li(5)-Cl(1) bond length is 2.37 Å. The Li(5)-Cl(5) bond length is 2.51 Å. $Al(1)^{3+}$ is bonded to one $Br(1)^{1-}$, one $Cl(3)^{1-}$, one $Cl(4)^{1-}$, and one $Cl(5)^{1-}$ atom to form $AlBrCl_3$ tetrahedra that share a corner with one $Li(2)Br_2Cl_2$ tetrahedra, a corner with one $Li(3)Br_2Cl_2$ tetrahedra, a corner with one $Li(5)Br_2Cl_2$ tetrahedra, a corner with one $Li(1)BrCl_3$ tetrahedra, and a corner with one $Li(4)BrCl_3$ tetrahedra. The Al(1)-Br(1) bond length is 2.30 Å. The Al(1)-Cl(3) bond length is 2.13 Å. The Al(1)-Cl(4) bond length is 2.15 Å. The Al(1)-Cl(5) bond length is 2.17 Å. There are three inequivalent Br^{1-} sites. In the first Br^{1-} site, $Br(1)^{1-}$ is bonded in a water-like geometry to one $Li(2)^{1+}$ and one $Al(1)^{3+}$ atom. In the second Br^{1-} site, $Br(2)^{1-}$ is bonded in a distorted see-saw-like geometry to one $Li(1)^{1+}$, one $Li(2)^{1+}$, one $Li(3)^{1+}$, and one $Li(5)^{1+}$ atom. In the third Br^{1-} site, $Br(3)^{1-}$ is bonded in a trigonal non-coplanar geometry to one $Li(3)^{1+}$, one $Li(4)^{1+}$, and one $Li(5)^{1+}$ atom. There are five inequivalent Cl^{1-} sites. In the first Cl^{1-} site, $Cl(1)^{1-}$ is bonded in a rectangular see-saw-like geometry to one $Li(1)^{1+}$, one $Li(2)^{1+}$, one $Li(4)^{1+}$, and one $Li(5)^{1+}$ atom. In the second Cl^{1-} site, $Cl(2)^{1-}$ is bonded in a distorted rectangular see-saw-like geometry to one $Li(1)^{1+}$, one $Li(2)^{1+}$, one $Li(3)^{1+}$, and one $Li(4)^{1+}$ atom. In the third Cl^{1-} site, $Cl(3)^{1-}$ is bonded in a bent 120 degrees geometry to one $Li(4)^{1+}$ and one $Al(1)^{3+}$ atom. In the fourth Cl^{1-} site, $Cl(4)^{1-}$ is bonded in a water-like geometry to one $Li(1)^{1+}$ and one $Al(1)^{3+}$ atom. In the fifth Cl^{1-} site, $Cl(5)^{1-}$ is bonded in a distorted trigonal non-coplanar geometry to one $Li(3)^{1+}$, one $Li(5)^{1+}$, and one $Al(1)^{3+}$ atom.

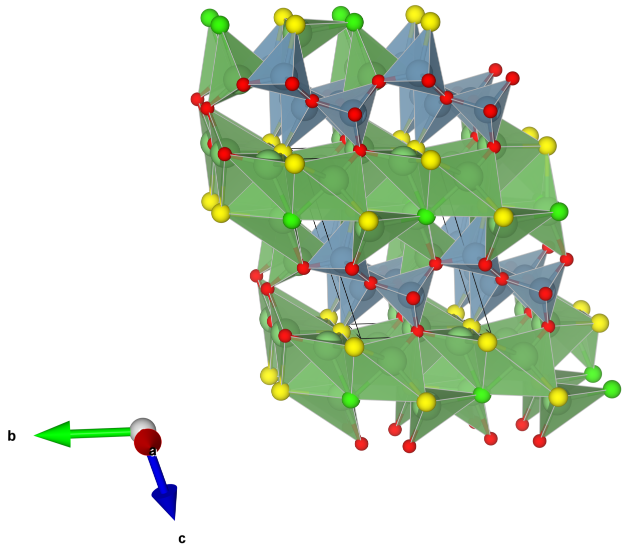

Figure S19: The predicted structure of $Li_6Al_3S_2O_5Cl$.

19. **$Li_6Al_3S_2O_5Cl$** crystallizes in the triclinic P1 space group. There are six inequivalent Li^{1+} sites. In the first Li^{1+} site, $Li(1)^{1+}$ is bonded in a 6-coordinate geometry to one $S(2)^{2-}$, two equivalent $S(1)^{2-}$, one $O(2)^{2-}$, and two equivalent $Cl(1)^{1-}$ atoms. The Li(1)-S(2) bond length is 2.80 Å. There is one shorter (2.61 Å) and one longer (3.04 Å) Li(1)-S(1) bond length. The Li(1)-O(2) bond length is 2.28 Å. There is one shorter (2.44 Å) and one longer (2.46 Å) Li(1)-Cl(1) bond length. In the second Li^{1+} site, $Li(2)^{1+}$ is bonded in a distorted single-bond geometry to two equivalent $S(2)^{2-}$, one $O(4)^{2-}$, and one $Cl(1)^{1-}$ atom. There is one shorter (2.51 Å) and one longer (2.68 Å) Li(2)-S(2) bond length. The Li(2)-O(4) bond length is 1.84 Å. The Li(2)-Cl(1) bond length is 2.54 Å. In the third Li^{1+} site, $Li(3)^{1+}$ is bonded in a 2-coordinate geometry to one $S(1)^{2-}$, one $S(2)^{2-}$, one $O(3)^{2-}$, and one $O(4)^{2-}$ atom. The Li(3)-S(1) bond length is 2.69 Å. The Li(3)-S(2) bond length is 2.49 Å. The Li(3)-O(3) bond length is 2.08 Å. The Li(3)-O(4) bond length is 1.88 Å. In the fourth Li^{1+} site, $Li(4)^{1+}$ is bonded in a distorted single-bond geometry to one $S(1)^{2-}$, one $S(2)^{2-}$, one $O(4)^{2-}$, and one $Cl(1)^{1-}$ atom. The Li(4)-S(1) bond length is 2.49 Å. The Li(4)-S(2) bond length is 2.54 Å. The Li(4)-O(4)

bond length is 1.87 Å. The Li(4)-Cl(1) bond length is 2.54 Å. In the fifth Li^{1+} site, $Li(5)^{1+}$ is bonded to one $O(1)^{2-}$, one $O(2)^{2-}$, one $O(3)^{2-}$, and one $Cl(1)^{1-}$ atom to form distorted $LiClO_3$ tetrahedra that share corners with two equivalent $Al(2)O_4$ tetrahedra, corners with two equivalent $Al(1)SO_3$ tetrahedra, and corners with two equivalent $Al(3)SO_3$ tetrahedra. The Li(5)-O(1) bond length is 1.95 Å. The Li(5)-O(2) bond length is 1.91 Å. The Li(5)-O(3) bond length is 1.90 Å. The Li(5)-Cl(1) bond length is 2.40 Å. In the sixth Li^{1+} site, $Li(6)^{1+}$ is bonded in a 4-coordinate geometry to two equivalent $S(1)^{2-}$, one $O(1)^{2-}$, and one $Cl(1)^{1-}$ atom. There is one shorter (2.47 Å) and one longer (2.56 Å) Li(6)-S(1) bond length. The Li(6)-O(1) bond length is 1.94 Å. The Li(6)-Cl(1) bond length is 2.42 Å. There are three inequivalent Al^{3+} sites. In the first Al^{3+} site, $Al(1)^{3+}$ is bonded to one $S(1)^{2-}$, one $O(1)^{2-}$, one $O(2)^{2-}$, and one $O(5)^{2-}$ atom to form $AlSO_3$ tetrahedra that share corners with two equivalent $Li(5)ClO_3$ tetrahedra, corners with two equivalent $Al(2)O_4$ tetrahedra, and corners with two equivalent $Al(3)SO_3$ tetrahedra. The Al(1)-S(1) bond length is 2.22 Å. The Al(1)-O(1) bond length is 1.76 Å. The Al(1)-O(2) bond length is 1.75 Å. The Al(1)-O(5) bond length is 1.80 Å. In the second Al^{3+} site, $Al(2)^{3+}$ is bonded to one $O(2)^{2-}$, one $O(3)^{2-}$, one $O(4)^{2-}$, and one $O(5)^{2-}$ atom to form AlO_4 tetrahedra that share corners with two equivalent $Li(5)ClO_3$ tetrahedra, corners with two equivalent $Al(1)SO_3$ tetrahedra, and corners with two equivalent $Al(3)SO_3$ tetrahedra. The Al(2)-O(2) bond length is 1.77 Å. The Al(2)-O(3) bond length is 1.76 Å. The Al(2)-O(4) bond length is 1.72 Å. The Al(2)-O(5) bond length is 1.83 Å. In the third Al^{3+} site, $Al(3)^{3+}$ is bonded to one $S(2)^{2-}$, one $O(1)^{2-}$, one $O(3)^{2-}$, and one $O(5)^{2-}$ atom to form $AlSO_3$ tetrahedra that share corners with two equivalent $Li(5)ClO_3$ tetrahedra, corners with two equivalent $Al(2)O_4$ tetrahedra, and corners with two equivalent $Al(1)SO_3$ tetrahedra. The Al(3)-S(2) bond length is 2.20 Å. The Al(3)-O(1) bond length is 1.76 Å. The Al(3)-O(3) bond length is 1.75 Å. The Al(3)-O(5) bond length is 1.79 Å. There are two inequivalent S^{2-} sites. In the first S^{2-} site, $S(1)^{2-}$ is bonded in a 7-coordinate geometry to one $Li(3)^{1+}$, one $Li(4)^{1+}$, two equivalent $Li(1)^{1+}$, two equivalent $Li(6)^{1+}$, and one $Al(1)^{3+}$ atom. In the second S^{2-} site, $S(2)^{2-}$ is bonded in a 6-coordinate geometry to one $Li(1)^{1+}$, one $Li(3)^{1+}$, one $Li(4)^{1+}$, two equivalent $Li(2)^{1+}$, and one $Al(3)^{3+}$ atom. There are five inequivalent O^{2-} sites. In the first O^{2-} site, $O(1)^{2-}$ is bonded in a tetrahedral geometry to one $Li(5)^{1+}$, one $Li(6)^{1+}$, one $Al(1)^{3+}$, and one $Al(3)^{3+}$ atom. In the second O^{2-} site, $O(2)^{2-}$ is bonded in a distorted trigonal pyramidal geometry to one $Li(1)^{1+}$, one $Li(5)^{1+}$, one $Al(1)^{3+}$, and one $Al(2)^{3+}$ atom. In the third O^{2-} site, $O(3)^{2-}$ is bonded in a tetrahedral geometry to one $Li(3)^{1+}$, one $Li(5)^{1+}$, one $Al(2)^{3+}$, and one $Al(3)^{3+}$ atom. In the fourth O^{2-} site, $O(4)^{2-}$ is bonded in a tetrahedral geometry to one $Li(2)^{1+}$, one $Li(3)^{1+}$, one $Li(4)^{1+}$, and one $Al(2)^{3+}$ atom. In the fifth O^{2-} site, $O(5)^{2-}$ is bonded in a trigonal planar geometry to one $Al(1)^{3+}$, one $Al(2)^{3+}$, and one $Al(3)^{3+}$ atom. $Cl(1)^{1-}$ is bonded in a distorted octahedral geometry to one $Li(2)^{1+}$, one $Li(4)^{1+}$, one $Li(5)^{1+}$, one $Li(6)^{1+}$, and two equivalent $Li(1)^{1+}$ atoms.

20. **$Li_2AlBSBr_5Cl$** crystallizes in the triclinic P1 space group. The structure is two-dimensional and consists of one $Li_2AlBSBr_5Cl$ sheet oriented in the (0, 0, 1) direction. There are two inequivalent Li^{1+} sites. In the first Li^{1+} site, $Li(1)^{1+}$ is bonded to one $S(1)^{2-}$, one $Br(1)^{1-}$, one $Br(2)^{1-}$, one $Br(4)^{1-}$, and one $Br(5)^{1-}$ atom to form distorted $LiSBr_4$ trigonal bipyramids that share corners with three equivalent $Li(2)SBr_3Cl$ trigonal bipyramids, an edge with one $B(1)SBr_2Cl$ tetrahedra, and edges with two equivalent $Al(1)SBr_3$ tetrahedra. The Li(1)-S(1) bond length is 2.54 Å. The Li(1)-Br(1) bond length is 2.66 Å. The Li(1)-Br(2) bond length is 2.80 Å. The Li(1)-Br(4) bond length is 2.78 Å. The Li(1)-Br(5) bond length is 2.67 Å. In the second Li^{1+} site, $Li(2)^{1+}$ is bonded to one $S(1)^{2-}$, one $Br(2)^{1-}$, one $Br(3)^{1-}$, one $Br(4)^{1-}$, and one $Cl(1)^{1-}$ atom to form distorted $LiSBr_3Cl$ trigonal bipyramids that share a corner with one $Al(1)SBr_3$ tetrahedra, corners with three equivalent $Li(1)SBr_4$ trigonal bipyramids, an edge with one $Al(1)SBr_3$ tetrahedra, and a faceface with one $B(1)SBr_2Cl$ tetrahedra. The Li(2)-S(1) bond length is 2.61 Å. The Li(2)-Br(2) bond length is 2.66 Å. The Li(2)-Br(3) bond length is 2.62 Å. The Li(2)-Br(4) bond length is 2.63 Å. The Li(2)-Cl(1) bond length is 2.55

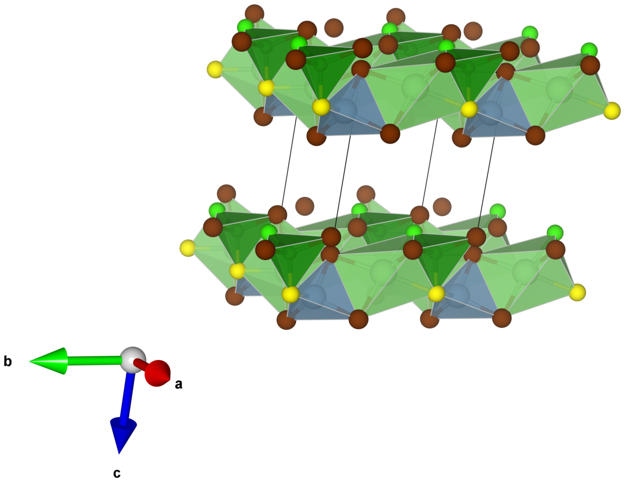

Figure S20: The predicted structure of $Li_2AlBSBr_5Cl$.

Å. $Al(1)^{3+}$ is bonded to one $S(1)^{2-}$, one $Br(1)^{1-}$, one $Br(2)^{1-}$, and one $Br(4)^{1-}$ atom to form $AlSBr_3$ tetrahedra that share a corner with one $B(1)SBr_2Cl$ tetrahedra, a corner with one $Li(2)SBr_3Cl$ trigonal bipyramid, an edge with one $Li(2)SBr_3Cl$ trigonal bipyramid, and edges with two equivalent $Li(1)SBr_4$ trigonal bipyramids. The Al(1)-S(1) bond length is 2.23 Å. The Al(1)-Br(1) bond length is 2.29 Å. The Al(1)-Br(2) bond length is 2.34 Å. The Al(1)-Br(4) bond length is 2.34 Å. $B(1)^{3+}$ is bonded to one $S(1)^{2-}$, one $Br(3)^{1-}$, one $Br(5)^{1-}$, and one $Cl(1)^{1-}$ atom to form $BSBr_2Cl$ tetrahedra that share a corner with one $Al(1)SBr_3$ tetrahedra, an edge with one $Li(1)SBr_4$ trigonal bipyramid, and a faceface with one $Li(2)SBr_3Cl$ trigonal bipyramid. The B(1)-S(1) bond length is 1.91 Å. The B(1)-Br(3) bond length is 2.06 Å. The B(1)-Br(5) bond length is 2.03 Å. The B(1)-Cl(1) bond length is 1.85 Å. $S(1)^{2-}$ is bonded in a 4-coordinate geometry to one $Li(1)^{1+}$, one $Li(2)^{1+}$, one $Al(1)^{3+}$, and one $B(1)^{3+}$ atom. There are five inequivalent Br^{1-} sites. In the first Br^{1-} site, $Br(1)^{1-}$ is bonded in an L-shaped geometry to one $Li(1)^{1+}$ and one $Al(1)^{3+}$ atom. In the second Br^{1-} site, $Br(2)^{1-}$ is bonded in a 3-coordinate geometry to one $Li(1)^{1+}$, one $Li(2)^{1+}$, and one $Al(1)^{3+}$ atom. In the third Br^{1-} site, $Br(3)^{1-}$ is bonded in a 2-coordinate geometry to one $Li(2)^{1+}$ and one $B(1)^{3+}$ atom. In the fourth Br^{1-} site, $Br(4)^{1-}$ is bonded in a distorted T-shaped geometry to one $Li(1)^{1+}$, one $Li(2)^{1+}$, and one $Al(1)^{3+}$ atom. In the fifth Br^{1-} site, $Br(5)^{1-}$ is bonded in a distorted L-shaped geometry to one $Li(1)^{1+}$ and one $B(1)^{3+}$ atom. $Cl(1)^{1-}$ is bonded in a 2-coordinate geometry to one $Li(2)^{1+}$ and one $B(1)^{3+}$ atom.

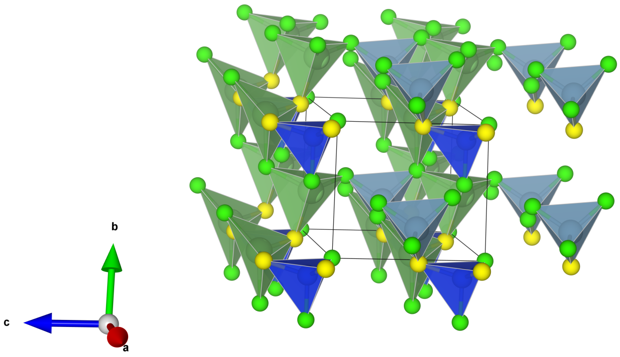

Figure S21: The predicted structure of $Li_2AlBSBr_5Cl$.

21. **$Li_2AlSiS_2Cl_5$** crystallizes in the triclinic P1 space group. There are two inequivalent Li^{1+}

sites. In the first Li^{1+} site, $Li(1)^{1+}$ is bonded to one $S(1)^{2-}$, one $S(2)^{2-}$, one $Cl(3)^{1-}$, and one $Cl(4)^{1-}$ atom to form distorted LiS_2Cl_2 trigonal pyramids that share corners with two equivalent $Li(2)SCl_3$ tetrahedra, corners with two equivalent $Si(1)S_2Cl_2$ tetrahedra, and corners with three equivalent $Al(1)SCl_3$ tetrahedra. The Li(1)-S(1) bond length is 2.56 Å. The Li(1)-S(2) bond length is 2.43 Å. The Li(1)-Cl(3) bond length is 2.52 Å. The Li(1)-Cl(4) bond length is 2.40 Å. In the second Li^{1+} site, $Li(2)^{1+}$ is bonded to one $S(2)^{2-}$, one $Cl(1)^{1-}$, one $Cl(3)^{1-}$, and one $Cl(5)^{1-}$ atom to form $LiSCl_3$ tetrahedra that share corners with two equivalent $Al(1)SCl_3$ tetrahedra, corners with two equivalent $Si(1)S_2Cl_2$ tetrahedra, and corners with two equivalent $Li(1)S_2Cl_2$ trigonal pyramids. The Li(2)-S(2) bond length is 2.39 Å. The Li(2)-Cl(1) bond length is 2.35 Å. The Li(2)-Cl(3) bond length is 2.45 Å. The Li(2)-Cl(5) bond length is 2.42 Å. $Al(1)^{3+}$ is bonded to one $S(1)^{2-}$, one $Cl(1)^{1-}$, one $Cl(3)^{1-}$, and one $Cl(4)^{1-}$ atom to form $AlSCl_3$ tetrahedra that share a corner with one $Si(1)S_2Cl_2$ tetrahedra, corners with two equivalent $Li(2)SCl_3$ tetrahedra, and corners with three equivalent $Li(1)S_2Cl_2$ trigonal pyramids. The Al(1)-S(1) bond length is 2.24 Å. The Al(1)-Cl(1) bond length is 2.15 Å. The Al(1)-Cl(3) bond length is 2.18 Å. The Al(1)-Cl(4) bond length is 2.15 Å. $Si(1)^{4+}$ is bonded to one $S(1)^{2-}$, one $S(2)^{2-}$, one $Cl(2)^{1-}$, and one $Cl(5)^{1-}$ atom to form SiS_2Cl_2 tetrahedra that share a corner with one $Al(1)SCl_3$ tetrahedra, corners with two equivalent $Li(2)SCl_3$ tetrahedra, and corners with two equivalent $Li(1)S_2Cl_2$ trigonal pyramids. The Si(1)-S(1) bond length is 2.13 Å. The Si(1)-S(2) bond length is 2.03 Å. The Si(1)-Cl(2) bond length is 2.06 Å. The Si(1)-Cl(5) bond length is 2.10 Å. There are two inequivalent S^{2-} sites. In the first S^{2-} site, $S(1)^{2-}$ is bonded in a trigonal non-coplanar geometry to one $Li(1)^{1+}$, one $Al(1)^{3+}$, and one $Si(1)^{4+}$ atom. In the second S^{2-} site, $S(2)^{2-}$ is bonded in a trigonal non-coplanar geometry to one $Li(1)^{1+}$, one $Li(2)^{1+}$, and one $Si(1)^{4+}$ atom. There are five inequivalent Cl^{1-} sites. In the first Cl^{1-} site, $Cl(1)^{1-}$ is bonded in a water-like geometry to one $Li(2)^{1+}$ and one $Al(1)^{3+}$ atom. In the second Cl^{1-} site, $Cl(2)^{1-}$ is bonded in a single-bond geometry to one $Si(1)^{4+}$ atom. In the third Cl^{1-} site, $Cl(3)^{1-}$ is bonded in a distorted trigonal non-coplanar geometry to one $Li(1)^{1+}$, one $Li(2)^{1+}$, and one $Al(1)^{3+}$ atom. In the fourth Cl^{1-} site, $Cl(4)^{1-}$ is bonded in a water-like geometry to one $Li(1)^{1+}$ and one $Al(1)^{3+}$ atom. In the fifth Cl^{1-} site, $Cl(5)^{1-}$ is bonded in a bent 120 degrees geometry to one $Li(2)^{1+}$ and one $Si(1)^{4+}$ atom.

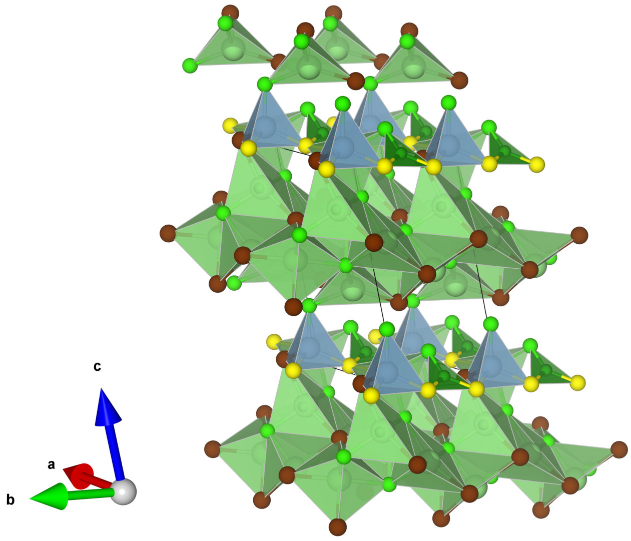

Figure S22: The predicted structure of $Li_7AlBS_2Br_4Cl_5$.

22. **$Li_7AlBS_2Br_4Cl_5$** crystallizes in the triclinic P1 space group. There are seven inequivalent Li^{1+} sites. In the first Li^{1+} site, $Li(1)^{1+}$ is bonded to one $Br(2)^{1-}$, one $Br(4)^{1-}$, one $Cl(2)^{1-}$, and one $Cl(4)^{1-}$ atom to form $LiBr_2Cl_2$ trigonal pyramids that share a corner with one $Li(7)Br_3Cl_2$ square pyramid, corners with three equivalent $Li(5)Br_3Cl_2$ square pyramids, a corner with one

$Al(1)S_2BrCl$ tetrahedra, corners with two equivalent $Li(4)Br_2Cl_3$ trigonal bipyramids, an edge with one $Li(2)Br_2Cl_3$ square pyramid, and an edge with one $Li(7)Br_3Cl_2$ square pyramid. The Li(1)-Br(2) bond length is 2.58 Å. The Li(1)-Br(4) bond length is 2.44 Å. The Li(1)-Cl(2) bond length is 2.35 Å. The Li(1)-Cl(4) bond length is 2.40 Å. In the second Li^{1+} site, $Li(2)^{1+}$ is bonded to one $Br(1)^{1-}$, one $Br(2)^{1-}$, one $Cl(1)^{1-}$, one $Cl(2)^{1-}$, and one $Cl(5)^{1-}$ atom to form distorted $LiBr_2Cl_3$ square pyramids that share corners with four equivalent $Li(5)Br_3Cl_2$ square pyramids, corners with three equivalent $Li(6)Br_2Cl_2$ tetrahedra, edges with two equivalent $Li(7)Br_3Cl_2$ square pyramids, an edge with one $Li(4)Br_2Cl_3$ trigonal bipyramid, an edge with one $Li(1)Br_2Cl_2$ trigonal pyramid, and a faceface with one $Li(4)Br_2Cl_3$ trigonal bipyramid. The Li(2)-Br(1) bond length is 2.59 Å. The Li(2)-Br(2) bond length is 2.67 Å. The Li(2)-Cl(1) bond length is 2.94 Å. The Li(2)-Cl(2) bond length is 2.45 Å. The Li(2)-Cl(5) bond length is 2.43 Å. In the third Li^{1+} site, $Li(3)^{1+}$ is bonded in a 5-coordinate geometry to one $S(1)^{2-}$, one $S(2)^{2-}$, one $Br(3)^{1-}$, one $Cl(3)^{1-}$, and one $Cl(5)^{1-}$ atom. The Li(3)-S(1) bond length is 2.50 Å. The Li(3)-S(2) bond length is 2.99 Å. The Li(3)-Br(3) bond length is 2.80 Å. The Li(3)-Cl(3) bond length is 2.49 Å. The Li(3)-Cl(5) bond length is 2.32 Å. In the fourth Li^{1+} site, $Li(4)^{1+}$ is bonded to one $Br(1)^{1-}$, one $Br(2)^{1-}$, one $Cl(1)^{1-}$, one $Cl(2)^{1-}$, and one $Cl(5)^{1-}$ atom to form $LiBr_2Cl_3$ trigonal bipyramids that share corners with four equivalent $Li(7)Br_3Cl_2$ square pyramids, a corner with one $Li(6)Br_2Cl_2$ tetrahedra, corners with two equivalent $Li(1)Br_2Cl_2$ trigonal pyramids, an edge with one $Li(2)Br_2Cl_3$ square pyramid, edges with two equivalent $Li(5)Br_3Cl_2$ square pyramids, an edge with one $Li(6)Br_2Cl_2$ tetrahedra, and a faceface with one $Li(2)Br_2Cl_3$ square pyramid. The Li(4)-Br(1) bond length is 2.66 Å. The Li(4)-Br(2) bond length is 2.66 Å. The Li(4)-Cl(1) bond length is 2.50 Å. The Li(4)-Cl(2) bond length is 2.42 Å. The Li(4)-Cl(5) bond length is 2.58 Å. In the fifth Li^{1+} site, $Li(5)^{1+}$ is bonded to one $Br(1)^{1-}$, one $Br(2)^{1-}$, one $Br(4)^{1-}$, one $Cl(1)^{1-}$, and one $Cl(2)^{1-}$ atom to form $LiBr_3Cl_2$ square pyramids that share corners with four equivalent $Li(2)Br_2Cl_3$ square pyramids, corners with three equivalent $Li(1)Br_2Cl_2$ trigonal pyramids, an edge with one $Li(7)Br_3Cl_2$ square pyramid, an edge with one $Li(6)Br_2Cl_2$ tetrahedra, edges with two equivalent $Li(4)Br_2Cl_3$ trigonal bipyramids, and a faceface with one $Li(7)Br_3Cl_2$ square pyramid. The Li(5)-Br(1) bond length is 2.86 Å. The Li(5)-Br(2) bond length is 2.67 Å. The Li(5)-Br(4) bond length is 2.57 Å. The Li(5)-Cl(1) bond length is 2.40 Å. The Li(5)-Cl(2) bond length is 2.66 Å. In the sixth Li^{1+} site, $Li(6)^{1+}$ is bonded to one $Br(1)^{1-}$, one $Br(3)^{1-}$, one $Cl(1)^{1-}$, and one $Cl(5)^{1-}$ atom to form distorted $LiBr_2Cl_2$ tetrahedra that share corners with two equivalent $Li(7)Br_3Cl_2$ square pyramids, corners with three equivalent $Li(2)Br_2Cl_3$ square pyramids, a corner with one $Al(1)S_2BrCl$ tetrahedra, a corner with one $Li(4)Br_2Cl_3$ trigonal bipyramid, an edge with one $Li(5)Br_3Cl_2$ square pyramid, and an edge with one $Li(4)Br_2Cl_3$ trigonal bipyramid. The Li(6)-Br(1) bond length is 2.57 Å. The Li(6)-Br(3) bond length is 2.64 Å. The Li(6)-Cl(1) bond length is 2.34 Å. The Li(6)-Cl(5) bond length is 2.31 Å. In the seventh Li^{1+} site, $Li(7)^{1+}$ is bonded to one $Br(1)^{1-}$, one $Br(2)^{1-}$, one $Br(4)^{1-}$, one $Cl(1)^{1-}$, and one $Cl(2)^{1-}$ atom to form $LiBr_3Cl_2$ square pyramids that share corners with two equivalent $Li(6)Br_2Cl_2$ tetrahedra, corners with four equivalent $Li(4)Br_2Cl_3$ trigonal bipyramids, a corner with one $Li(1)Br_2Cl_2$ trigonal pyramid, an edge with one $Li(5)Br_3Cl_2$ square pyramid, edges with two equivalent $Li(2)Br_2Cl_3$ square pyramids, an edge with one $Li(1)Br_2Cl_2$ trigonal pyramid, and a faceface with one $Li(5)Br_3Cl_2$ square pyramid. The Li(7)-Br(1) bond length is 2.78 Å. The Li(7)-Br(2) bond length is 2.61 Å. The Li(7)-Br(4) bond length is 2.68 Å. The Li(7)-Cl(1) bond length is 2.44 Å. The Li(7)-Cl(2) bond length is 2.54 Å. $Al(1)^{3+}$ is bonded to one $S(1)^{2-}$, one $S(2)^{2-}$, one $Br(3)^{1-}$, and one $Cl(4)^{1-}$ atom to form AlS_2BrCl tetrahedra that share a corner with one $Li(6)Br_2Cl_2$ tetrahedra and a corner with one $Li(1)Br_2Cl_2$ trigonal pyramid. The Al(1)-S(1) bond length is 2.25 Å. The Al(1)-S(2) bond length is 2.24 Å. The Al(1)-Br(3) bond length is 2.33 Å. The Al(1)-Cl(4) bond length is 2.14 Å. $B(1)^{3+}$ is bonded in a trigonal planar geometry to one $S(1)^{2-}$, one $S(2)^{2-}$, and one $Cl(3)^{1-}$ atom. The B(1)-S(1) bond length is 1.80 Å. The B(1)-S(2) bond length is 1.78 Å. The B(1)-Cl(3) bond length is 1.79 Å. There are two inequivalent S^{2-} sites. In the

first S^{2-} site, $S(1)^{2-}$ is bonded in a distorted T-shaped geometry to one $Li(3)^{1+}$, one $Al(1)^{3+}$, and one $B(1)^{3+}$ atom. In the second S^{2-} site, $S(2)^{2-}$ is bonded in a 2-coordinate geometry to one $Li(3)^{1+}$, one $Al(1)^{3+}$, and one $B(1)^{3+}$ atom. There are four inequivalent Br^{1-} sites. In the first Br^{1-} site, $Br(1)^{1-}$ is bonded in a 5-coordinate geometry to one $Li(2)^{1+}$, one $Li(4)^{1+}$, one $Li(5)^{1+}$, one $Li(6)^{1+}$, and one $Li(7)^{1+}$ atom. In the second Br^{1-} site, $Br(2)^{1-}$ is bonded in a distorted trigonal bipyramidal geometry to one $Li(1)^{1+}$, one $Li(2)^{1+}$, one $Li(4)^{1+}$, one $Li(5)^{1+}$, and one $Li(7)^{1+}$ atom. In the third Br^{1-} site, $Br(3)^{1-}$ is bonded in a 3-coordinate geometry to one $Li(3)^{1+}$, one $Li(6)^{1+}$, and one $Al(1)^{3+}$ atom. In the fourth Br^{1-} site, $Br(4)^{1-}$ is bonded in a 3-coordinate geometry to one $Li(1)^{1+}$, one $Li(5)^{1+}$, and one $Li(7)^{1+}$ atom. There are five inequivalent Cl^{1-} sites. In the first Cl^{1-} site, $Cl(1)^{1-}$ is bonded in a 5-coordinate geometry to one $Li(2)^{1+}$, one $Li(4)^{1+}$, one $Li(5)^{1+}$, one $Li(6)^{1+}$, and one $Li(7)^{1+}$ atom. In the second Cl^{1-} site, $Cl(2)^{1-}$ is bonded in a distorted trigonal bipyramidal geometry to one $Li(1)^{1+}$, one $Li(2)^{1+}$, one $Li(4)^{1+}$, one $Li(5)^{1+}$, and one $Li(7)^{1+}$ atom. In the third Cl^{1-} site, $Cl(3)^{1-}$ is bonded in a water-like geometry to one $Li(3)^{1+}$ and one $B(1)^{3+}$ atom. In the fourth Cl^{1-} site, $Cl(4)^{1-}$ is bonded in a water-like geometry to one $Li(1)^{1+}$ and one $Al(1)^{3+}$ atom. In the fifth Cl^{1-} site, $Cl(5)^{1-}$ is bonded in a distorted square co-planar geometry to one $Li(2)^{1+}$, one $Li(3)^{1+}$, one $Li(4)^{1+}$, and one $Li(6)^{1+}$ atom.

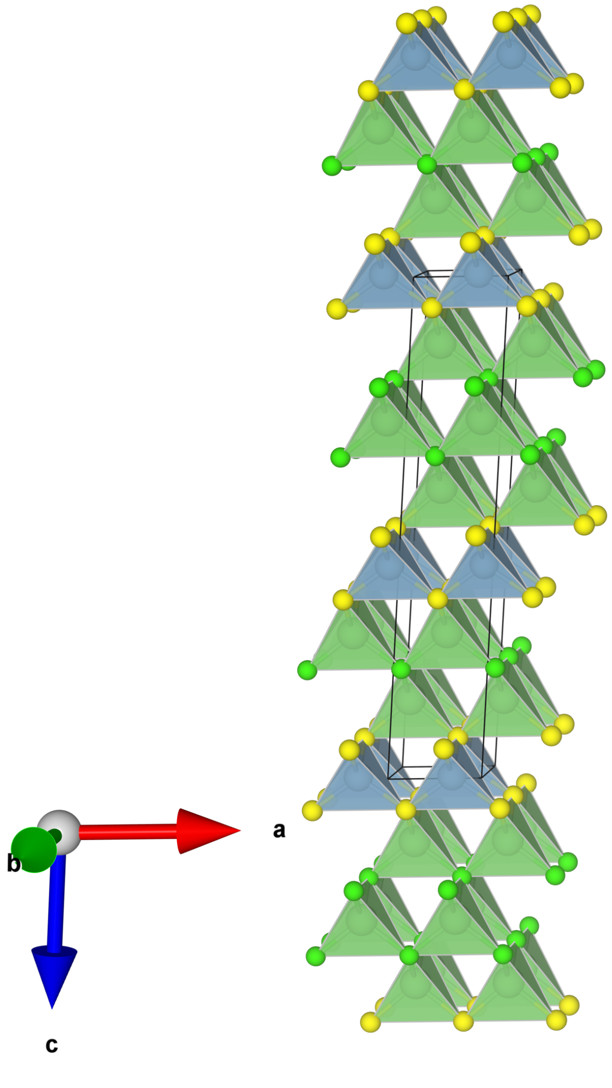

Figure S23: The predicted structure of $Li_5Al_2S_4Cl_3$.

23. **$Li_5Al_2S_4Cl_3$** is Stannite-like structured and crystallizes in the triclinic P1 space group. There are five inequivalent Li^{1+} sites. In the first Li^{1+} site, $Li(1)^{1+}$ is bonded to two equivalent $S(2)^{2-}$ and two equivalent $Cl(1)^{1-}$ atoms to form LiS_2Cl_2 tetrahedra that share corners with four equivalent $Li(2)Cl_4$ tetrahedra, corners with four equivalent $Li(1)S_2Cl_2$ tetrahedra, and corners with four equivalent $Al(1)S_4$ tetrahedra. Both Li(1)-S(2) bond lengths are 2.40 Å. There is one shorter (2.33 Å) and one longer (2.35 Å) Li(1)-Cl(1) bond length. In the second Li^{1+} site, $Li(2)^{1+}$ is bonded to two equivalent $Cl(1)^{1-}$ and two equivalent $Cl(2)^{1-}$ atoms to form $LiCl_4$ tetrahedra that share corners with four equivalent $Li(2)Cl_4$ tetrahedra, corners with four equivalent $Li(1)S_2Cl_2$ tetrahedra, and corners with four equivalent $Li(3)S_2Cl_2$ tetrahedra. There is one shorter (2.33 Å) and one longer (2.34 Å) Li(2)-Cl(1) bond length. There is one shorter (2.33 Å) and one longer (2.35 Å) Li(2)-Cl(2) bond length. In the third Li^{1+} site, $Li(3)^{1+}$ is bonded to two equivalent $S(3)^{2-}$ and two equivalent $Cl(2)^{1-}$ atoms to form LiS_2Cl_2 tetrahedra that share corners with four equivalent $Li(2)Cl_4$ tetrahedra, corners with four equivalent $Li(3)S_2Cl_2$ tetrahedra, and corners with four equivalent $Al(2)S_4$ tetrahedra. Both Li(3)-S(3) bond lengths are 2.41 Å. Both Li(3)-Cl(2) bond lengths are 2.33 Å. In the fourth Li^{1+} site, $Li(4)^{1+}$ is bonded to two equivalent $S(4)^{2-}$ and two equivalent $Cl(3)^{1-}$ atoms to form LiS_2Cl_2 tetrahedra that share corners with four equivalent $Li(4)S_2Cl_2$ tetrahedra, corners with four equivalent $Li(5)S_2Cl_2$ tetrahedra, and corners with four equivalent $Al(2)S_4$ tetrahedra. Both Li(4)-S(4) bond lengths are 2.40 Å. Both Li(4)-Cl(3) bond lengths are 2.34 Å. In the fifth Li^{1+} site, $Li(5)^{1+}$ is bonded to two equivalent $S(1)^{2-}$ and two equivalent $Cl(3)^{1-}$ atoms to form LiS_2Cl_2 tetrahedra that share corners with four equivalent $Li(4)S_2Cl_2$ tetrahedra, corners with four equivalent $Li(5)S_2Cl_2$ tetrahedra, and corners with four equivalent $Al(1)S_4$ tetrahedra. There is one shorter (2.40 Å) and one longer (2.41 Å) Li(5)-S(1) bond length. There is one shorter (2.33 Å) and one longer (2.34 Å) Li(5)-Cl(3) bond length. There are two inequivalent Al^{3+} sites. In the first Al^{3+} site, $Al(1)^{3+}$ is bonded to two equivalent $S(1)^{2-}$ and two equivalent $S(2)^{2-}$ atoms to form AlS_4 tetrahedra that share corners with four equivalent $Li(1)S_2Cl_2$ tetrahedra, corners with four equivalent $Li(5)S_2Cl_2$ tetrahedra, and corners with four equivalent $Al(1)S_4$ tetrahedra. Both Al(1)-S(1) bond lengths are 2.27 Å. Both Al(1)-S(2) bond lengths are 2.27 Å. In the second Al^{3+} site, $Al(2)^{3+}$ is bonded to two equivalent $S(3)^{2-}$ and two equivalent $S(4)^{2-}$ atoms to form AlS_4 tetrahedra that share corners with four equivalent $Li(3)S_2Cl_2$ tetrahedra, corners with four equivalent $Li(4)S_2Cl_2$ tetrahedra, and corners with four equivalent $Al(2)S_4$ tetrahedra. Both Al(2)-S(3) bond lengths are 2.27 Å. Both Al(2)-S(4) bond lengths are 2.27 Å. There are four inequivalent S^{2-} sites. In the first S^{2-} site, $S(1)^{2-}$ is bonded in a tetrahedral geometry to two equivalent $Li(5)^{1+}$ and two equivalent $Al(1)^{3+}$ atoms. In the second S^{2-} site, $S(2)^{2-}$ is bonded in a tetrahedral geometry to two equivalent $Li(1)^{1+}$ and two equivalent $Al(1)^{3+}$ atoms. In the third S^{2-} site, $S(3)^{2-}$ is bonded in a tetrahedral geometry to two equivalent $Li(3)^{1+}$ and two equivalent $Al(2)^{3+}$ atoms. In the fourth S^{2-} site, $S(4)^{2-}$ is bonded in a tetrahedral geometry to two equivalent $Li(4)^{1+}$ and two equivalent $Al(2)^{3+}$ atoms. There are three inequivalent Cl^{1-} sites. In the first Cl^{1-} site, $Cl(1)^{1-}$ is bonded in a tetrahedral geometry to two equivalent $Li(1)^{1+}$ and two equivalent $Li(2)^{1+}$ atoms. In the second Cl^{1-} site, $Cl(2)^{1-}$ is bonded in a tetrahedral geometry to two equivalent $Li(2)^{1+}$ and two equivalent $Li(3)^{1+}$ atoms. In the third Cl^{1-} site, $Cl(3)^{1-}$ is bonded in a tetrahedral geometry to two equivalent $Li(4)^{1+}$ and two equivalent $Li(5)^{1+}$ atoms.

24. **$Li_2Al_2S_3BrCl$** crystallizes in the triclinic P1 space group. There are two inequivalent Li^{1+} sites. In the first Li^{1+} site, $Li(1)^{1+}$ is bonded to one $S(1)^{2-}$, one $S(3)^{2-}$, one $Br(1)^{1-}$, and one $Cl(1)^{1-}$ atom to form LiS_2BrCl tetrahedra that share corners with three equivalent $Li(2)S_2BrCl$ tetrahedra, corners with three equivalent $Al(2)S_3Br$ tetrahedra, and corners with three equivalent $Al(1)S_3Cl$ tetrahedra. The Li(1)-S(1) bond length is 2.41 Å. The Li(1)-S(3) bond length is 2.45 Å. The Li(1)-Br(1) bond length is 2.55 Å. The Li(1)-Cl(1) bond length is 2.40 Å. In the second Li^{1+} site, $Li(2)^{1+}$ is bonded to one $S(2)^{2-}$, one $S(3)^{2-}$, one $Br(1)^{1-}$, and one $Cl(1)^{1-}$ atom to form LiS_2BrCl tetrahedra that share corners with three equivalent $Li(1)S_2BrCl$ tetra-

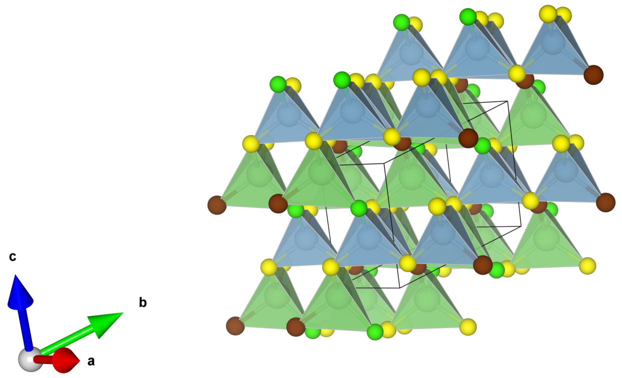

Figure S24: The predicted structure of $Li_2Al_2S_3BrCl$.

hedra, corners with three equivalent $Al(2)S_3Br$ tetrahedra, and corners with three equivalent $Al(1)S_3Cl$ tetrahedra. The Li(2)-S(2) bond length is 2.40 Å. The Li(2)-S(3) bond length is 2.44 Å. The Li(2)-Br(1) bond length is 2.56 Å. The Li(2)-Cl(1) bond length is 2.41 Å. There are two inequivalent Al^{3+} sites. In the first Al^{3+} site, $Al(1)^{3+}$ is bonded to one $S(1)^{2-}$, one $S(2)^{2-}$, one $S(3)^{2-}$, and one $Cl(1)^{1-}$ atom to form AlS_3Cl tetrahedra that share corners with three equivalent $Li(1)S_2BrCl$ tetrahedra, corners with three equivalent $Li(2)S_2BrCl$ tetrahedra, and corners with three equivalent $Al(2)S_3Br$ tetrahedra. The Al(1)-S(1) bond length is 2.22 Å. The Al(1)-S(2) bond length is 2.22 Å. The Al(1)-S(3) bond length is 2.25 Å. The Al(1)-Cl(1) bond length is 2.23 Å. In the second Al^{3+} site, $Al(2)^{3+}$ is bonded to one $S(1)^{2-}$, one $S(2)^{2-}$, one $S(3)^{2-}$, and one $Br(1)^{1-}$ atom to form AlS_3Br tetrahedra that share corners with three equivalent $Li(1)S_2BrCl$ tetrahedra, corners with three equivalent $Li(2)S_2BrCl$ tetrahedra, and corners with three equivalent $Al(1)S_3Cl$ tetrahedra. The Al(2)-S(1) bond length is 2.22 Å. The Al(2)-S(2) bond length is 2.22 Å. The Al(2)-S(3) bond length is 2.25 Å. The Al(2)-Br(1) bond length is 2.39 Å. There are three inequivalent S^{2-} sites. In the first S^{2-} site, $S(1)^{2-}$ is bonded in a trigonal non-coplanar geometry to one $Li(1)^{1+}$, one $Al(1)^{3+}$, and one $Al(2)^{3+}$ atom. In the second S^{2-} site, $S(2)^{2-}$ is bonded in a trigonal non-coplanar geometry to one $Li(2)^{1+}$, one $Al(1)^{3+}$, and one $Al(2)^{3+}$ atom. In the third S^{2-} site, $S(3)^{2-}$ is bonded in a tetrahedral geometry to one $Li(1)^{1+}$, one $Li(2)^{1+}$, one $Al(1)^{3+}$, and one $Al(2)^{3+}$ atom. $Br(1)^{1-}$ is bonded in a trigonal non-coplanar geometry to one $Li(1)^{1+}$, one $Li(2)^{1+}$, and one $Al(2)^{3+}$ atom. $Cl(1)^{1-}$ is bonded in a trigonal non-coplanar geometry to one $Li(1)^{1+}$, one $Li(2)^{1+}$, and one $Al(1)^{3+}$ atom.

25. **$Li_6Al_2S_3Br_5Cl$** crystallizes in the triclinic P1 space group. There are six inequivalent Li^{1+} sites. In the first Li^{1+} site, $Li(1)^{1+}$ is bonded to one $Br(4)^{1-}$, two equivalent $Br(5)^{1-}$, and two equivalent $Cl(1)^{1-}$ atoms to form $LiBr_3Cl_2$ square pyramids that share corners with two equivalent $Li(3)Br_4Cl_2$ octahedra, a corner with one $Li(4)SBr_3$ tetrahedra, edges with three equivalent $Li(3)Br_4Cl_2$ octahedra, edges with two equivalent $Li(1)Br_3Cl_2$ square pyramids, and edges with two equivalent $Li(6)Br_4Cl$ square pyramids. The corner-sharing octahedral tilt angles are 5°. The Li(1)-Br(4) bond length is 2.67 Å. Both Li(1)-Br(5) bond lengths are 2.61 Å. There is one shorter (2.58 Å) and one longer (2.59 Å) Li(1)-Cl(1) bond length. In the second Li^{1+} site, $Li(2)^{1+}$ is bonded in a trigonal non-coplanar geometry to one $Br(5)^{1-}$ and two equivalent $Br(2)^{1-}$ atoms. The Li(2)-Br(5) bond length is 2.40 Å. There is one shorter (2.44 Å) and one longer (2.47 Å) Li(2)-Br(2) bond length. In the third Li^{1+} site, $Li(3)^{1+}$ is bonded to one $Br(3)^{1-}$, one $Br(5)^{1-}$, two equivalent $Br(4)^{1-}$, and two equivalent $Cl(1)^{1-}$ atoms to form distorted $LiBr_4Cl_2$ octahedra that share corners with two equivalent $Li(1)Br_3Cl_2$ square pyramids, corners with two equivalent $Li(6)Br_4Cl$ square pyramids, edges with two equivalent $Li(3)Br_4Cl_2$ octahedra, edges with three equivalent $Li(1)Br_3Cl_2$ square pyramids, edges with three equivalent $Li(6)Br_4Cl$ square pyramids, and edges with two equivalent $Li(4)SBr_3$ tetrahedra. The Li(3)-Br(3) bond length is 3.11 Å. The Li(3)-Br(5) bond length is 2.63 Å. There is one shorter (2.80 Å) and one

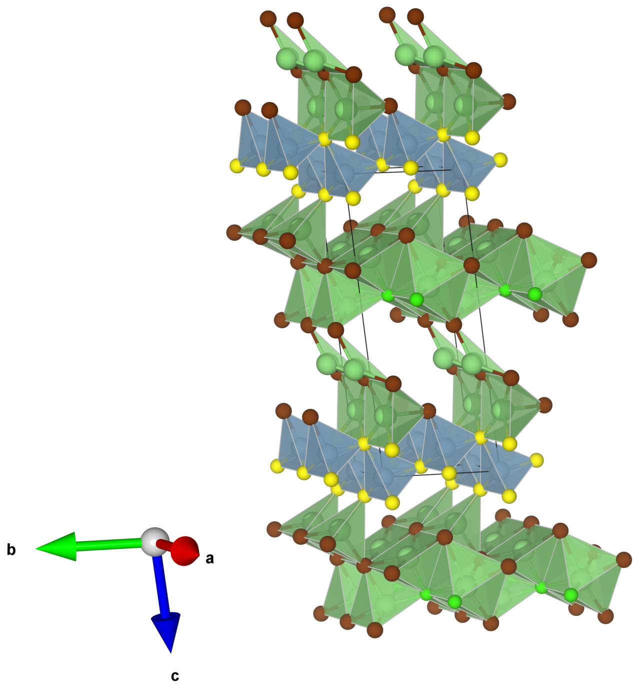

Figure S25: The predicted structure of $Li_6Al_2S_3Br_5Cl$.

longer (2.83 Å) Li(3)-Br(4) bond length. There is one shorter (2.48 Å) and one longer (2.49 Å) Li(3)-Cl(1) bond length. In the fourth Li^{1+} site, $Li(4)^{1+}$ is bonded to one $S(1)^{2-}$, one $Br(4)^{1-}$, and two equivalent $Br(3)^{1-}$ atoms to form $LiSBr_3$ tetrahedra that share a corner with one $Li(1)Br_3Cl_2$ square pyramid, corners with four equivalent $Li(6)Br_4Cl$ square pyramids, corners with two equivalent $Li(4)SBr_3$ tetrahedra, corners with two equivalent $Al(1)S_4$ tetrahedra, edges with two equivalent $Li(3)Br_4Cl_2$ octahedra, and an edge with one $Li(6)Br_4Cl$ square pyramid. The Li(4)-S(1) bond length is 2.38 Å. The Li(4)-Br(4) bond length is 2.56 Å. There is one shorter (2.45 Å) and one longer (2.46 Å) Li(4)-Br(3) bond length. In the fifth Li^{1+} site, $Li(5)^{1+}$ is bonded to two equivalent $S(2)^{2-}$, one $Br(1)^{1-}$, and two equivalent $Br(2)^{1-}$ atoms to form LiS_2Br_3 square pyramids that share corners with two equivalent $Al(1)S_4$ tetrahedra, corners with three equivalent $Al(2)S_3Br$ tetrahedra, and edges with two equivalent $Li(5)S_2Br_3$ square pyramids. There is one shorter (2.52 Å) and one longer (2.55 Å) Li(5)-S(2) bond length. The Li(5)-Br(1) bond length is 2.58 Å. Both Li(5)-Br(2) bond lengths are 2.71 Å. In the sixth Li^{1+} site, $Li(6)^{1+}$ is bonded to two equivalent $Br(3)^{1-}$, two equivalent $Br(4)^{1-}$, and one $Cl(1)^{1-}$ atom to form $LiBr_4Cl$ square pyramids that share corners with two equivalent $Li(3)Br_4Cl_2$ octahedra, corners with four equivalent $Li(4)SBr_3$ tetrahedra, edges with three equivalent $Li(3)Br_4Cl_2$ octahedra, edges with two equivalent $Li(1)Br_3Cl_2$ square pyramids, edges with two equivalent $Li(6)Br_4Cl$ square pyramids, and an edge with one $Li(4)SBr_3$ tetrahedra. The corner-sharing octahedral tilt angles range from 13-14°. There is one shorter (2.60 Å) and one longer (2.63 Å) Li(6)-Br(3) bond length. There is one shorter (2.65 Å) and one longer (2.68 Å) Li(6)-Br(4) bond length. The Li(6)-Cl(1) bond length is 2.39 Å. There are two inequivalent Al^{3+} sites. In the first Al^{3+} site, $Al(1)^{3+}$ is bonded to one $S(2)^{2-}$, one $S(3)^{2-}$, and two equivalent $S(1)^{2-}$ atoms to form AlS_4 tetrahedra that share corners with two equivalent $Li(5)S_2Br_3$ square pyramids, corners with two equivalent $Li(4)SBr_3$ tetrahedra, corners with two equivalent $Al(1)S_4$ tetrahedra, and corners with three equivalent $Al(2)S_3Br$ tetrahedra. The Al(1)-S(2) bond length is 2.25 Å. The Al(1)-S(3) bond length is 2.33 Å. Both Al(1)-S(1) bond lengths are 2.23 Å. In the second Al^{3+} site, $Al(2)^{3+}$ is bonded to one $S(2)^{2-}$, two equivalent $S(3)^{2-}$, and one $Br(1)^{1-}$ atom to form AlS_3Br tetrahedra that share corners with three equivalent $Li(5)S_2Br_3$ square pyramids, corners with two equivalent $Al(2)S_3Br$ tetrahedra, and corners with three equivalent $Al(1)S_4$

tetrahedra. The Al(2)-S(2) bond length is 2.22 Å. Both Al(2)-S(3) bond lengths are 2.29 Å. The Al(2)-Br(1) bond length is 2.33 Å. There are three inequivalent S^{2-} sites. In the first S^{2-} site, $S(1)^{2-}$ is bonded in a trigonal planar geometry to one $Li(4)^{1+}$ and two equivalent $Al(1)^{3+}$ atoms. In the second S^{2-} site, $S(2)^{2-}$ is bonded in a tetrahedral geometry to two equivalent $Li(5)^{1+}$, one $Al(1)^{3+}$, and one $Al(2)^{3+}$ atom. In the third S^{2-} site, $S(3)^{2-}$ is bonded in a trigonal non-coplanar geometry to one $Al(1)^{3+}$ and two equivalent $Al(2)^{3+}$ atoms. There are five inequivalent Br^{1-} sites. In the first Br^{1-} site, $Br(1)^{1-}$ is bonded in a bent 120 degrees geometry to one $Li(5)^{1+}$ and one $Al(2)^{3+}$ atom. In the second Br^{1-} site, $Br(2)^{1-}$ is bonded in a 4-coordinate geometry to two equivalent $Li(2)^{1+}$ and two equivalent $Li(5)^{1+}$ atoms. In the third Br^{1-} site, $Br(3)^{1-}$ is bonded in a 5-coordinate geometry to one $Li(3)^{1+}$, two equivalent $Li(4)^{1+}$, and two equivalent $Li(6)^{1+}$ atoms. In the fourth Br^{1-} site, $Br(4)^{1-}$ is bonded in an octahedral geometry to one $Li(1)^{1+}$, one $Li(4)^{1+}$, two equivalent $Li(3)^{1+}$, and two equivalent $Li(6)^{1+}$ atoms. In the fifth Br^{1-} site, $Br(5)^{1-}$ is bonded in a rectangular see-saw-like geometry to one $Li(2)^{1+}$, one $Li(3)^{1+}$, and two equivalent $Li(1)^{1+}$ atoms. $Cl(1)^{1-}$ is bonded in a square pyramidal geometry to one $Li(6)^{1+}$, two equivalent $Li(1)^{1+}$, and two equivalent $Li(3)^{1+}$ atoms.

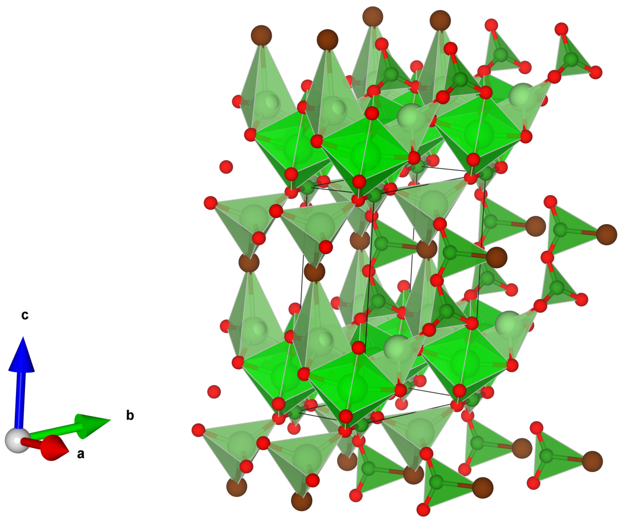

Figure S26: The predicted structure of $Li_4Zr_2B_3O_{10}Br$.

26. **$Li_4Zr_2B_3O_{10}Br$** crystallizes in the triclinic P1 space group. There are four inequivalent Li^{1+} sites. In the first Li^{1+} site, $Li(1)^{1+}$ is bonded to one $O(2)^{2-}$, one $O(3)^{2-}$, one $O(4)^{2-}$, and one $Br(1)^{1-}$ atom to form distorted $LiBrO_3$ tetrahedra that share a corner with one $Zr(1)O_6$ octahedra and a corner with one $Zr(2)O_6$ octahedra. The corner-sharing octahedral tilt angles range from 63-69°. The Li(1)-O(2) bond length is 1.87 Å. The Li(1)-O(3) bond length is 1.99 Å. The Li(1)-O(4) bond length is 2.01 Å. The Li(1)-Br(1) bond length is 2.61 Å. In the second Li^{1+} site, $Li(2)^{1+}$ is bonded in a 3-coordinate geometry to one $O(6)^{2-}$, one $O(7)^{2-}$, one $O(8)^{2-}$, and one $Br(1)^{1-}$ atom. The Li(2)-O(6) bond length is 2.05 Å. The Li(2)-O(7) bond length is 1.97 Å. The Li(2)-O(8) bond length is 1.86 Å. The Li(2)-Br(1) bond length is 2.99 Å. In the third Li^{1+} site, $Li(3)^{1+}$ is bonded in a see-saw-like geometry to one $O(1)^{2-}$, one $O(5)^{2-}$, one $O(6)^{2-}$, and one $O(8)^{2-}$ atom. The Li(3)-O(1) bond length is 1.92 Å. The Li(3)-O(5) bond length is 2.02 Å. The Li(3)-O(6) bond length is 2.00 Å. The Li(3)-O(8) bond length is 1.90 Å. In the fourth Li^{1+} site, $Li(4)^{1+}$ is bonded in a rectangular see-saw-like geometry to one $O(2)^{2-}$, one $O(3)^{2-}$, one $O(5)^{2-}$, and one $O(9)^{2-}$ atom. The Li(4)-O(2) bond length is 1.83 Å. The Li(4)-O(3) bond length is 2.09 Å. The Li(4)-O(5) bond length is 1.98 Å. The Li(4)-O(9) bond length is 1.92 Å. There are two inequivalent Zr^{4+} sites. In the first Zr^{4+} site, $Zr(1)^{4+}$ is bonded to one $O(4)^{2-}$, one $O(5)^{2-}$, one $O(6)^{2-}$, one $O(7)^{2-}$, one $O(8)^{2-}$, and one $O(9)^{2-}$ atom to form ZrO_6 octahedra that share corners with four equivalent $Zr(2)O_6$ octahedra and

a corner with one Li(1)BrO_3 tetrahedra. The corner-sharing octahedral tilt angles range from 32-57°. The Zr(1)-O(4) bond length is 2.32 Å. The Zr(1)-O(5) bond length is 2.01 Å. The Zr(1)-O(6) bond length is 2.14 Å. The Zr(1)-O(7) bond length is 2.10 Å. The Zr(1)-O(8) bond length is 2.22 Å. The Zr(1)-O(9) bond length is 1.98 Å. In the second Zr^{4+} site, $Zr(2)^{4+}$ is bonded to one $O(1)^{2-}$, one $O(3)^{2-}$, one $O(5)^{2-}$, one $O(6)^{2-}$, one $O(8)^{2-}$, and one $O(9)^{2-}$ atom to form distorted ZrO_6 octahedra that share corners with four equivalent Zr(1)O_6 octahedra and a corner with one Li(1)BrO_3 tetrahedra. The corner-sharing octahedral tilt angles range from 32-57°. The Zr(2)-O(1) bond length is 2.06 Å. The Zr(2)-O(3) bond length is 2.40 Å. The Zr(2)-O(5) bond length is 2.12 Å. The Zr(2)-O(6) bond length is 2.05 Å. The Zr(2)-O(8) bond length is 2.02 Å. The Zr(2)-O(9) bond length is 2.09 Å. There are three inequivalent B^{3+} sites. In the first B^{3+} site, $B(1)^{3+}$ is bonded in a trigonal planar geometry to one $O(1)^{2-}$, one $O(10)^{2-}$, and one $O(7)^{2-}$ atom. The B(1)-O(1) bond length is 1.36 Å. The B(1)-O(10) bond length is 1.38 Å. The B(1)-O(7) bond length is 1.36 Å. In the second B^{3+} site, $B(2)^{3+}$ is bonded in a linear geometry to one $O(3)^{2-}$ and one $O(4)^{2-}$ atom. The B(2)-O(3) bond length is 1.26 Å. The B(2)-O(4) bond length is 1.26 Å. In the third B^{3+} site, $B(3)^{3+}$ is bonded in a distorted trigonal planar geometry to one $O(10)^{2-}$, one $O(2)^{2-}$, and one $Br(1)^{1-}$ atom. The B(3)-O(10) bond length is 1.34 Å. The B(3)-O(2) bond length is 1.29 Å. The B(3)-Br(1) bond length is 2.12 Å. There are ten inequivalent O^{2-} sites. In the first O^{2-} site, $O(1)^{2-}$ is bonded in a distorted T-shaped geometry to one $Li(3)^{1+}$, one $Zr(2)^{4+}$, and one $B(1)^{3+}$ atom. In the second O^{2-} site, $O(2)^{2-}$ is bonded in a trigonal planar geometry to one $Li(1)^{1+}$, one $Li(4)^{1+}$, and one $B(3)^{3+}$ atom. In the third O^{2-} site, $O(3)^{2-}$ is bonded in a 4-coordinate geometry to one $Li(1)^{1+}$, one $Li(4)^{1+}$, one $Zr(2)^{4+}$, and one $B(2)^{3+}$ atom. In the fourth O^{2-} site, $O(4)^{2-}$ is bonded in a distorted trigonal planar geometry to one $Li(1)^{1+}$, one $Zr(1)^{4+}$, and one $B(2)^{3+}$ atom. In the fifth O^{2-} site, $O(5)^{2-}$ is bonded in a rectangular see-saw-like geometry to one $Li(3)^{1+}$, one $Li(4)^{1+}$, one $Zr(1)^{4+}$, and one $Zr(2)^{4+}$ atom. In the sixth O^{2-} site, $O(6)^{2-}$ is bonded in a distorted rectangular see-saw-like geometry to one $Li(2)^{1+}$, one $Li(3)^{1+}$, one $Zr(1)^{4+}$, and one $Zr(2)^{4+}$ atom. In the seventh O^{2-} site, $O(7)^{2-}$ is bonded in a distorted trigonal non-coplanar geometry to one $Li(2)^{1+}$, one $Zr(1)^{4+}$, and one $B(1)^{3+}$ atom. In the eighth O^{2-} site, $O(8)^{2-}$ is bonded in a distorted see-saw-like geometry to one $Li(2)^{1+}$, one $Li(3)^{1+}$, one $Zr(1)^{4+}$, and one $Zr(2)^{4+}$ atom. In the ninth O^{2-} site, $O(9)^{2-}$ is bonded in a distorted trigonal planar geometry to one $Li(4)^{1+}$, one $Zr(1)^{4+}$, and one $Zr(2)^{4+}$ atom. In the tenth O^{2-} site, $O(10)^{2-}$ is bonded in a distorted bent 120 degrees geometry to one $B(1)^{3+}$ and one $B(3)^{3+}$ atom. $Br(1)^{1-}$ is bonded in a 3-coordinate geometry to one $Li(1)^{1+}$, one $Li(2)^{1+}$, and one $B(3)^{3+}$ atom.

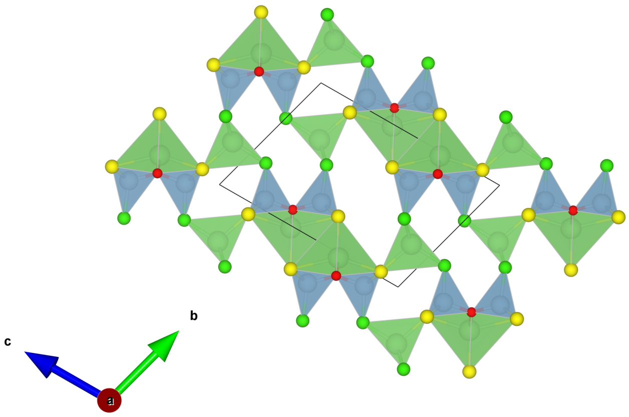

Figure S27: The predicted structure of $Li_2Al_2S_2OCl_2$.

27. **$Li_2Al_2S_2OCl_2$** crystallizes in the triclinic P1 space group. There are four inequivalent Li^{1+} sites. In the first Li^{1+} site, $Li(1)^{1+}$ is bonded to one $S(2)^{2-}$, one $Cl(2)^{1-}$, and two equivalent $Cl(3)^{1-}$

atoms to form $LiSCl_3$ tetrahedra that share a corner with one $Al(3)S_2ClO$ tetrahedra, corners with two equivalent $Li(1)SCl_3$ tetrahedra, corners with two equivalent $Al(1)S_2ClO$ tetrahedra, and corners with two equivalent $Al(2)S_2ClO$ tetrahedra. The Li(1)-S(2) bond length is 2.41 Å. The Li(1)-Cl(2) bond length is 2.33 Å. There is one shorter (2.37 Å) and one longer (2.40 Å) Li(1)-Cl(3) bond length. In the second Li^{1+} site, $Li(2)^{1+}$ is bonded in a 5-coordinate geometry to one $S(1)^{2-}$, one $S(2)^{2-}$, one $S(4)^{2-}$, and two equivalent $O(2)^{2-}$ atoms. The Li(2)-S(1) bond length is 2.43 Å. The Li(2)-S(2) bond length is 2.63 Å. The Li(2)-S(4) bond length is 2.89 Å. There is one shorter (2.10 Å) and one longer (2.11 Å) Li(2)-O(2) bond length. In the third Li^{1+} site, $Li(3)^{1+}$ is bonded to one $S(3)^{2-}$, one $Cl(4)^{1-}$, and two equivalent $Cl(1)^{1-}$ atoms to form $LiSCl_3$ tetrahedra that share a corner with one $Al(1)S_2ClO$ tetrahedra, corners with two equivalent $Li(3)SCl_3$ tetrahedra, corners with two equivalent $Al(3)S_2ClO$ tetrahedra, and corners with two equivalent $Al(4)S_2ClO$ tetrahedra. The Li(3)-S(3) bond length is 2.41 Å. The Li(3)-Cl(4) bond length is 2.35 Å. There is one shorter (2.37 Å) and one longer (2.39 Å) Li(3)-Cl(1) bond length. In the fourth Li^{1+} site, $Li(4)^{1+}$ is bonded in a 5-coordinate geometry to one $S(1)^{2-}$, one $S(3)^{2-}$, one $S(4)^{2-}$, and two equivalent $O(1)^{2-}$ atoms. The Li(4)-S(1) bond length is 2.88 Å. The Li(4)-S(3) bond length is 2.63 Å. The Li(4)-S(4) bond length is 2.43 Å. There is one shorter (2.10 Å) and one longer (2.11 Å) Li(4)-O(1) bond length. There are four inequivalent Al^{3+} sites. In the first Al^{3+} site, $Al(1)^{3+}$ is bonded to two equivalent $S(2)^{2-}$, one $O(2)^{2-}$, and one $Cl(4)^{1-}$ atom to form AlS_2ClO tetrahedra that share a corner with one $Li(3)SCl_3$ tetrahedra, a corner with one $Al(2)S_2ClO$ tetrahedra, corners with two equivalent $Li(1)SCl_3$ tetrahedra, and corners with two equivalent $Al(1)S_2ClO$ tetrahedra. Both Al(1)-S(2) bond lengths are 2.23 Å. The Al(1)-O(2) bond length is 1.75 Å. The Al(1)-Cl(4) bond length is 2.16 Å. In the second Al^{3+} site, $Al(2)^{3+}$ is bonded to two equivalent $S(4)^{2-}$, one $O(2)^{2-}$, and one $Cl(3)^{1-}$ atom to form AlS_2ClO tetrahedra that share a corner with one $Al(1)S_2ClO$ tetrahedra, corners with two equivalent $Li(1)SCl_3$ tetrahedra, and corners with two equivalent $Al(2)S_2ClO$ tetrahedra. There is one shorter (2.22 Å) and one longer (2.23 Å) Al(2)-S(4) bond length. The Al(2)-O(2) bond length is 1.74 Å. The Al(2)-Cl(3) bond length is 2.23 Å. In the third Al^{3+} site, $Al(3)^{3+}$ is bonded to two equivalent $S(3)^{2-}$, one $O(1)^{2-}$, and one $Cl(2)^{1-}$ atom to form AlS_2ClO tetrahedra that share a corner with one $Li(1)SCl_3$ tetrahedra, a corner with one $Al(4)S_2ClO$ tetrahedra, corners with two equivalent $Li(3)SCl_3$ tetrahedra, and corners with two equivalent $Al(3)S_2ClO$ tetrahedra. There is one shorter (2.22 Å) and one longer (2.23 Å) Al(3)-S(3) bond length. The Al(3)-O(1) bond length is 1.75 Å. The Al(3)-Cl(2) bond length is 2.16 Å. In the fourth Al^{3+} site, $Al(4)^{3+}$ is bonded to two equivalent $S(1)^{2-}$, one $O(1)^{2-}$, and one $Cl(1)^{1-}$ atom to form AlS_2ClO tetrahedra that share a corner with one $Al(3)S_2ClO$ tetrahedra, corners with two equivalent $Li(3)SCl_3$ tetrahedra, and corners with two equivalent $Al(4)S_2ClO$ tetrahedra. Both Al(4)-S(1) bond lengths are 2.22 Å. The Al(4)-O(1) bond length is 1.74 Å. The Al(4)-Cl(1) bond length is 2.23 Å. There are four inequivalent S^{2-} sites. In the first S^{2-} site, $S(1)^{2-}$ is bonded in a 4-coordinate geometry to one $Li(2)^{1+}$, one $Li(4)^{1+}$, and two equivalent $Al(4)^{3+}$ atoms. In the second S^{2-} site, $S(2)^{2-}$ is bonded in a 4-coordinate geometry to one $Li(1)^{1+}$, one $Li(2)^{1+}$, and two equivalent $Al(1)^{3+}$ atoms. In the third S^{2-} site, $S(3)^{2-}$ is bonded in a 4-coordinate geometry to one $Li(3)^{1+}$, one $Li(4)^{1+}$, and two equivalent $Al(3)^{3+}$ atoms. In the fourth S^{2-} site, $S(4)^{2-}$ is bonded in a distorted trigonal non-coplanar geometry to one $Li(2)^{1+}$, one $Li(4)^{1+}$, and two equivalent $Al(2)^{3+}$ atoms. There are two inequivalent O^{2-} sites. In the first O^{2-} site, $O(1)^{2-}$ is bonded in a distorted see-saw-like geometry to two equivalent $Li(4)^{1+}$, one $Al(3)^{3+}$, and one $Al(4)^{3+}$ atom. In the second O^{2-} site, $O(2)^{2-}$ is bonded in a distorted see-saw-like geometry to two equivalent $Li(2)^{1+}$, one $Al(1)^{3+}$, and one $Al(2)^{3+}$ atom. There are four inequivalent Cl^{1-} sites. In the first Cl^{1-} site, $Cl(1)^{1-}$ is bonded in a distorted trigonal planar geometry to two equivalent $Li(3)^{1+}$ and one $Al(4)^{3+}$ atom. In the second Cl^{1-} site, $Cl(2)^{1-}$ is bonded in a bent 120 degrees geometry to one $Li(1)^{1+}$ and one $Al(3)^{3+}$ atom. In the third Cl^{1-} site, $Cl(3)^{1-}$ is bonded in a distorted trigonal planar geometry to two equivalent $Li(1)^{1+}$ and one $Al(2)^{3+}$ atom. In the fourth Cl^{1-} site, $Cl(4)^{1-}$ is bonded in

a bent 120 degrees geometry to one $Li(3)^{1+}$ and one $Al(1)^{3+}$ atom.

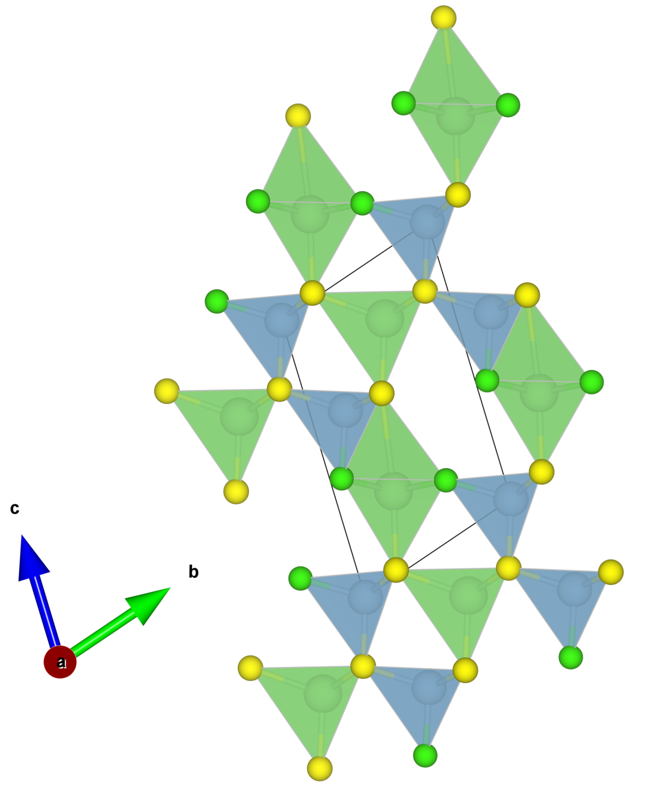

Figure S28: The predicted structure of $Li_2Al_2S_3Cl_2$.

28. $\mathbf{Li_2Al_2S_3Cl_2}$ is Chalcostibite-derived structured and crystallizes in the triclinic P1 space group. There are two inequivalent Li^{1+} sites. In the first Li^{1+} site, $Li(1)^{1+}$ is bonded to one $S(1)^{2-}$, one $S(2)^{2-}$, and two equivalent $S(3)^{2-}$ atoms to form LiS_4 tetrahedra that share corners with two equivalent $Li(1)S_4$ tetrahedra, corners with four equivalent $Al(1)S_3Cl$ tetrahedra, and corners with four equivalent $Al(2)S_3Cl$ tetrahedra. The Li(1)-S(1) bond length is 2.49 Å. The Li(1)-S(2) bond length is 2.43 Å. Both Li(1)-S(3) bond lengths are 2.41 Å. In the second Li^{1+} site, $Li(2)^{1+}$ is bonded in a 5-coordinate geometry to one $S(1)^{2-}$, two equivalent $Cl(1)^{1-}$, and two equivalent $Cl(2)^{1-}$ atoms. The Li(2)-S(1) bond length is 2.56 Å. There is one shorter (2.51 Å) and one longer (2.52 Å) Li(2)-Cl(1) bond length. There is one shorter (2.51 Å) and one longer (2.52 Å) Li(2)-Cl(2) bond length. There are two inequivalent Al^{3+} sites. In the first Al^{3+} site, $Al(1)^{3+}$ is bonded to one $S(3)^{2-}$, two equivalent $S(2)^{2-}$, and one $Cl(1)^{1-}$ atom to form AlS_3Cl tetrahedra that share a corner with one $Al(2)S_3Cl$ tetrahedra, corners with two equivalent $Al(1)S_3Cl$ tetrahedra, and corners with four equivalent $Li(1)S_4$ tetrahedra. The Al(1)-S(3) bond length is 2.25 Å. Both Al(1)-S(2) bond lengths are 2.24 Å. The Al(1)-Cl(1) bond length is 2.20 Å. In the second Al^{3+} site, $Al(2)^{3+}$ is bonded to one $S(3)^{2-}$, two equivalent $S(1)^{2-}$, and one $Cl(2)^{1-}$ atom to form AlS_3Cl tetrahedra that share a corner with one $Al(1)S_3Cl$ tetrahedra, corners with two equivalent $Al(2)S_3Cl$ tetrahedra, and corners with four equivalent $Li(1)S_4$ tetrahedra. The Al(2)-S(3) bond length is 2.23 Å. Both Al(2)-S(1) bond lengths are 2.25 Å. The Al(2)-Cl(2) bond length is 2.19 Å. There are three inequivalent S^{2-} sites. In the first S^{2-} site, $S(1)^{2-}$ is bonded in a tetrahedral geometry to one $Li(1)^{1+}$, one $Li(2)^{1+}$, and two equivalent $Al(2)^{3+}$ atoms. In the second S^{2-} site, $S(2)^{2-}$ is bonded in a distorted trigonal non-coplanar geometry to one $Li(1)^{1+}$ and two equivalent $Al(1)^{3+}$ atoms. In the third S^{2-} site, $S(3)^{2-}$ is bonded in a tetrahedral geometry to two equivalent $Li(1)^{1+}$, one $Al(1)^{3+}$, and one $Al(2)^{3+}$ atom. There are two inequivalent Cl^{1-} sites. In the first Cl^{1-} site, $Cl(1)^{1-}$ is bonded in a 3-coordinate geometry to two equivalent $Li(2)^{1+}$ and one $Al(1)^{3+}$ atom. In the second Cl^{1-}

site, $Cl(2)^{1-}$ is bonded in a distorted trigonal planar geometry to two equivalent $Li(2)^{1+}$ and one $Al(2)^{3+}$ atom.

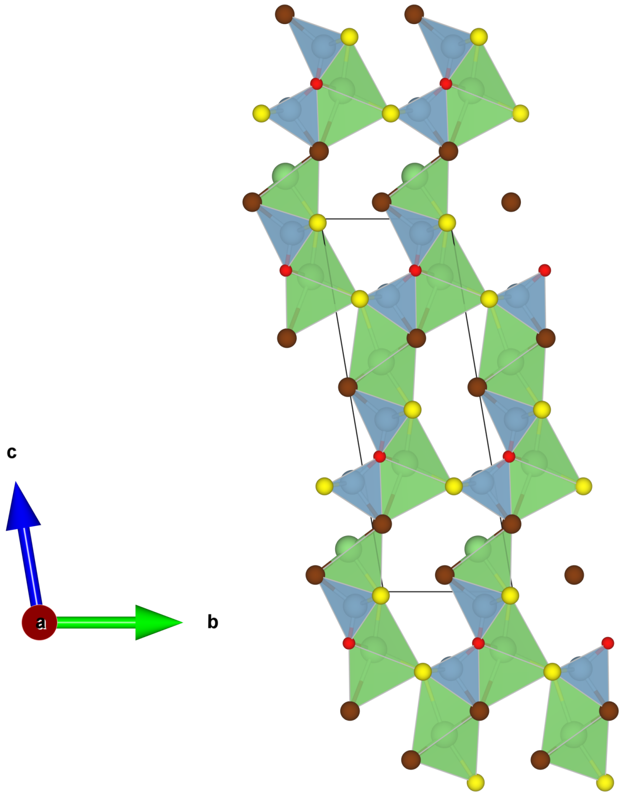

Figure S29: The predicted structure of $Li_2Al_2S_2OBr_2$.

29. **$Li_2Al_2S_2OBr_2$** crystallizes in the triclinic P1 space group. There are four inequivalent Li^{1+} sites. In the first Li^{1+} site, $Li(1)^{1+}$ is bonded to one $S(2)^{2-}$, one $S(4)^{2-}$, and two equivalent $O(1)^{2-}$ atoms to form distorted LiS_2O_2 trigonal pyramids that share a corner with one $Li(2)S_2Br_4$ octahedra, a corner with one $Li(3)S_2Br_4$ octahedra, corners with four equivalent $Al(2)S_2BrO$ tetrahedra, corners with two equivalent $Li(1)S_2O_2$ trigonal pyramids, and edges with two equivalent $Al(1)S_2BrO$ tetrahedra. The corner-sharing octahedral tilt angles range from 42-47°. The Li(1)-S(2) bond length is 2.51 Å. The Li(1)-S(4) bond length is 2.53 Å. Both Li(1)-O(1) bond lengths are 2.17 Å. In the second Li^{1+} site, $Li(2)^{1+}$ is bonded to one $S(1)^{2-}$, one $S(2)^{2-}$, two equivalent $Br(3)^{1-}$, and two equivalent $Br(4)^{1-}$ atoms to form LiS_2Br_4 octahedra that share a corner with one $Li(1)S_2O_2$ trigonal pyramid, a corner with one $Li(4)S_2O_2$ trigonal pyramid, edges with two equivalent $Li(2)S_2Br_4$ octahedra, edges with two equivalent $Al(2)S_2BrO$ tetrahedra, and edges with two equivalent $Al(3)S_2BrO$ tetrahedra. The Li(2)-S(1) bond length is 2.66 Å. The Li(2)-S(2) bond length is 3.09 Å. There is one shorter (2.66 Å) and one longer (2.69 Å) Li(2)-Br(3) bond length. Both Li(2)-Br(4) bond lengths are 2.66 Å. In the third Li^{1+} site, $Li(3)^{1+}$ is bonded to one $S(3)^{2-}$, one $S(4)^{2-}$, two equivalent $Br(1)^{1-}$, and two equivalent $Br(2)^{1-}$ atoms to form distorted LiS_2Br_4 octahedra that share a corner with one $Li(1)S_2O_2$ trigonal pyramid, a corner with one $Li(4)S_2O_2$ trigonal pyramid, edges with two equivalent $Li(3)S_2Br_4$ octahedra, edges with two equivalent $Al(1)S_2BrO$ tetrahedra, and edges with two equivalent $Al(4)S_2BrO$ tetrahedra. The Li(3)-S(3) bond length is 3.12 Å. The Li(3)-S(4) bond length is 2.63 Å. There is one shorter (2.65 Å) and one longer (2.67 Å) Li(3)-Br(1) bond length. There is one shorter (2.66 Å) and one longer (2.68 Å) Li(3)-Br(2) bond length. In the fourth Li^{1+} site, $Li(4)^{1+}$ is bonded to one $S(1)^{2-}$, one $S(3)^{2-}$, and two equivalent $O(2)^{2-}$ atoms to form distorted LiS_2O_2 trigonal pyramids that share a corner with one $Li(2)S_2Br_4$

octahedra, a corner with one $Li(3)S_2Br_4$ octahedra, corners with four equivalent $Al(4)S_2BrO$ tetrahedra, corners with two equivalent $Li(4)S_2O_2$ trigonal pyramids, and edges with two equivalent $Al(3)S_2BrO$ tetrahedra. The corner-sharing octahedral tilt angles range from 41-44°. The Li(4)-S(1) bond length is 2.52 Å. The Li(4)-S(3) bond length is 2.51 Å. There is one shorter (2.18 Å) and one longer (2.19 Å) Li(4)-O(2) bond length. There are four inequivalent Al^{3+} sites. In the first Al^{3+} site, $Al(1)^{3+}$ is bonded to two equivalent $S(4)^{2-}$, one $O(1)^{2-}$, and one $Br(2)^{1-}$ atom to form AlS_2BrO tetrahedra that share a corner with one $Al(2)S_2BrO$ tetrahedra, corners with two equivalent $Al(1)S_2BrO$ tetrahedra, edges with two equivalent $Li(3)S_2Br_4$ octahedra, and edges with two equivalent $Li(1)S_2O_2$ trigonal pyramids. Both Al(1)-S(4) bond lengths are 2.23 Å. The Al(1)-O(1) bond length is 1.75 Å. The Al(1)-Br(2) bond length is 2.34 Å. In the second Al^{3+} site, $Al(2)^{3+}$ is bonded to two equivalent $S(2)^{2-}$, one $O(1)^{2-}$, and one $Br(3)^{1-}$ atom to form distorted AlS_2BrO tetrahedra that share a corner with one $Al(1)S_2BrO$ tetrahedra, corners with two equivalent $Al(2)S_2BrO$ tetrahedra, corners with four equivalent $Li(1)S_2O_2$ trigonal pyramids, and edges with two equivalent $Li(2)S_2Br_4$ octahedra. Both Al(2)-S(2) bond lengths are 2.24 Å. The Al(2)-O(1) bond length is 1.74 Å. The Al(2)-Br(3) bond length is 2.37 Å. In the third Al^{3+} site, $Al(3)^{3+}$ is bonded to two equivalent $S(1)^{2-}$, one $O(2)^{2-}$, and one $Br(4)^{1-}$ atom to form AlS_2BrO tetrahedra that share a corner with one $Al(4)S_2BrO$ tetrahedra, corners with two equivalent $Al(3)S_2BrO$ tetrahedra, edges with two equivalent $Li(2)S_2Br_4$ octahedra, and edges with two equivalent $Li(4)S_2O_2$ trigonal pyramids. Both Al(3)-S(1) bond lengths are 2.23 Å. The Al(3)-O(2) bond length is 1.75 Å. The Al(3)-Br(4) bond length is 2.35 Å. In the fourth Al^{3+} site, $Al(4)^{3+}$ is bonded to two equivalent $S(3)^{2-}$, one $O(2)^{2-}$, and one $Br(1)^{1-}$ atom to form distorted AlS_2BrO tetrahedra that share a corner with one $Al(3)S_2BrO$ tetrahedra, corners with two equivalent $Al(4)S_2BrO$ tetrahedra, corners with four equivalent $Li(4)S_2O_2$ trigonal pyramids, and edges with two equivalent $Li(3)S_2Br_4$ octahedra. Both Al(4)-S(3) bond lengths are 2.24 Å. The Al(4)-O(2) bond length is 1.74 Å. The Al(4)-Br(1) bond length is 2.38 Å. There are four inequivalent S^{2-} sites. In the first S^{2-} site, $S(1)^{2-}$ is bonded in a 4-coordinate geometry to one $Li(2)^{1+}$, one $Li(4)^{1+}$, and two equivalent $Al(3)^{3+}$ atoms. In the second S^{2-} site, $S(2)^{2-}$ is bonded in a 4-coordinate geometry to one $Li(1)^{1+}$, one $Li(2)^{1+}$, and two equivalent $Al(2)^{3+}$ atoms. In the third S^{2-} site, $S(3)^{2-}$ is bonded in a 4-coordinate geometry to one $Li(3)^{1+}$, one $Li(4)^{1+}$, and two equivalent $Al(4)^{3+}$ atoms. In the fourth S^{2-} site, $S(4)^{2-}$ is bonded in a 4-coordinate geometry to one $Li(1)^{1+}$, one $Li(3)^{1+}$, and two equivalent $Al(1)^{3+}$ atoms. There are two inequivalent O^{2-} sites. In the first O^{2-} site, $O(1)^{2-}$ is bonded in a distorted tetrahedral geometry to two equivalent $Li(1)^{1+}$, one $Al(1)^{3+}$, and one $Al(2)^{3+}$ atom. In the second O^{2-} site, $O(2)^{2-}$ is bonded in a distorted tetrahedral geometry to two equivalent $Li(4)^{1+}$, one $Al(3)^{3+}$, and one $Al(4)^{3+}$ atom. There are four inequivalent Br^{1-} sites. In the first Br^{1-} site, $Br(1)^{1-}$ is bonded in a distorted T-shaped geometry to two equivalent $Li(3)^{1+}$ and one $Al(4)^{3+}$ atom. In the second Br^{1-} site, $Br(2)^{1-}$ is bonded in a 3-coordinate geometry to two equivalent $Li(3)^{1+}$ and one $Al(1)^{3+}$ atom. In the third Br^{1-} site, $Br(3)^{1-}$ is bonded in a 3-coordinate geometry to two equivalent $Li(2)^{1+}$ and one $Al(2)^{3+}$ atom. In the fourth Br^{1-} site, $Br(4)^{1-}$ is bonded in a 3-coordinate geometry to two equivalent $Li(2)^{1+}$ and one $Al(3)^{3+}$ atom.

30. $\mathbf{Li_4ZrAl_4S_5(O_2Cl)_2}$ crystallizes in the triclinic P1 space group. There are four inequivalent Li^{1+} sites. In the first Li^{1+} site, $Li(1)^{1+}$ is bonded in a 4-coordinate geometry to one $S(2)^{2-}$, one $S(4)^{2-}$, one $O(2)^{2-}$, and one $Cl(1)^{1-}$ atom. The Li(1)-S(2) bond length is 2.57 Å. The Li(1)-S(4) bond length is 2.58 Å. The Li(1)-O(2) bond length is 2.03 Å. The Li(1)-Cl(1) bond length is 2.33 Å. In the second Li^{1+} site, $Li(2)^{1+}$ is bonded in a 4-coordinate geometry to one $S(1)^{2-}$, one $S(4)^{2-}$, one $O(1)^{2-}$, and one $Cl(1)^{1-}$ atom. The Li(2)-S(1) bond length is 2.51 Å. The Li(2)-S(4) bond length is 2.62 Å. The Li(2)-O(1) bond length is 1.94 Å. The Li(2)-Cl(1) bond length is 2.35 Å. In the third Li^{1+} site, $Li(3)^{1+}$ is bonded to one $S(3)^{2-}$, one $S(4)^{2-}$, one $O(1)^{2-}$, and one $Cl(1)^{1-}$ atom to form distorted LiS_2ClO tetrahedra that share a corner with one $Al(1)S_2O_2$ tetrahedra and a corner with one $Al(2)S_3O$ tetrahedra. The Li(3)-S(3) bond length

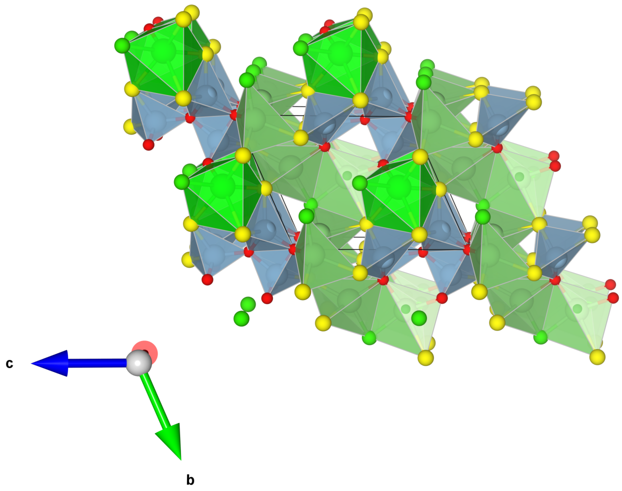

Figure S30: The predicted structure of $Li_4ZrAl_4S_5(O_2Cl)_2$.

is 2.53 Å. The Li(3)-S(4) bond length is 2.61 Å. The Li(3)-O(1) bond length is 1.92 Å. The Li(3)-Cl(1) bond length is 2.39 Å. In the fourth Li^{1+} site, $Li(4)^{1+}$ is bonded in a 5-coordinate geometry to one $S(1)^{2-}$, one $O(2)^{2-}$, one $O(3)^{2-}$, one $Cl(1)^{1-}$, and one $Cl(2)^{1-}$ atom. The Li(4)-S(1) bond length is 2.77 Å. The Li(4)-O(2) bond length is 1.99 Å. The Li(4)-O(3) bond length is 2.01 Å. The Li(4)-Cl(1) bond length is 2.41 Å. The Li(4)-Cl(2) bond length is 2.57 Å. $Zr(1)^{4+}$ is bonded in a 6-coordinate geometry to one $S(1)^{2-}$, one $S(2)^{2-}$, one $S(4)^{2-}$, one $S(5)^{2-}$, one $O(3)^{2-}$, and one $Cl(2)^{1-}$ atom. The Zr(1)-S(1) bond length is 2.66 Å. The Zr(1)-S(2) bond length is 2.47 Å. The Zr(1)-S(4) bond length is 2.57 Å. The Zr(1)-S(5) bond length is 2.81 Å. The Zr(1)-O(3) bond length is 1.94 Å. The Zr(1)-Cl(2) bond length is 2.64 Å. There are four inequivalent Al^{3+} sites. In the first Al^{3+} site, $Al(1)^{3+}$ is bonded to one $S(2)^{2-}$, one $S(5)^{2-}$, one $O(1)^{2-}$, and one $O(4)^{2-}$ atom to form distorted AlS_2O_2 tetrahedra that share a corner with one $Li(3)S_2ClO$ tetrahedra and a corner with one $Al(2)S_3O$ tetrahedra. The Al(1)-S(2) bond length is 2.17 Å. The Al(1)-S(5) bond length is 2.25 Å. The Al(1)-O(1) bond length is 1.76 Å. The Al(1)-O(4) bond length is 1.82 Å. In the second Al^{3+} site, $Al(2)^{3+}$ is bonded to one $S(1)^{2-}$, one $S(3)^{2-}$, one $S(5)^{2-}$, and one $O(2)^{2-}$ atom to form distorted AlS_3O tetrahedra that share a corner with one $Li(3)S_2ClO$ tetrahedra and a corner with one $Al(1)S_2O_2$ tetrahedra. The Al(2)-S(1) bond length is 2.31 Å. The Al(2)-S(3) bond length is 2.17 Å. The Al(2)-S(5) bond length is 2.24 Å. The Al(2)-O(2) bond length is 1.78 Å. In the third Al^{3+} site, $Al(3)^{3+}$ is bonded in a 5-coordinate geometry to one $S(4)^{2-}$, one $O(1)^{2-}$, one $O(3)^{2-}$, one $O(4)^{2-}$, and one $Cl(2)^{1-}$ atom. The Al(3)-S(4) bond length is 2.27 Å. The Al(3)-O(1) bond length is 1.83 Å. The Al(3)-O(3) bond length is 1.77 Å. The Al(3)-O(4) bond length is 1.84 Å. The Al(3)-Cl(2) bond length is 2.72 Å. In the fourth Al^{3+} site, $Al(4)^{3+}$ is bonded in a 4-coordinate geometry to one $S(1)^{2-}$, one $S(3)^{2-}$, one $O(2)^{2-}$, and one $O(4)^{2-}$ atom. The Al(4)-S(1) bond length is 2.35 Å. The Al(4)-S(3) bond length is 2.19 Å. The Al(4)-O(2) bond length is 1.78 Å. The Al(4)-O(4) bond length is 1.77 Å. There are five inequivalent S^{2-} sites. In the first S^{2-} site, $S(1)^{2-}$ is bonded in a 5-coordinate geometry to one $Li(2)^{1+}$, one $Li(4)^{1+}$, one $Zr(1)^{4+}$, one $Al(2)^{3+}$, and one $Al(4)^{3+}$ atom. In the second S^{2-} site, $S(2)^{2-}$ is bonded in a 3-coordinate geometry to one $Li(1)^{1+}$, one $Zr(1)^{4+}$, and one $Al(1)^{3+}$ atom. In the third S^{2-} site, $S(3)^{2-}$ is bonded in a trigonal non-coplanar geometry to one $Li(3)^{1+}$, one $Al(2)^{3+}$, and one $Al(4)^{3+}$ atom. In the fourth S^{2-} site, $S(4)^{2-}$ is bonded in a 5-coordinate geometry to one $Li(1)^{1+}$, one $Li(2)^{1+}$, one $Li(3)^{1+}$, one $Zr(1)^{4+}$, and one $Al(3)^{3+}$ atom. In the fifth S^{2-} site, $S(5)^{2-}$ is bonded in a 2-coordinate geometry to one $Zr(1)^{4+}$, one $Al(1)^{3+}$, and one $Al(2)^{3+}$ atom. There are four inequivalent O^{2-} sites. In the first O^{2-} site, $O(1)^{2-}$ is bonded in a tetrahedral geometry to one $Li(2)^{1+}$, one $Li(3)^{1+}$, one $Al(1)^{3+}$, and one $Al(3)^{3+}$ atom. In the second O^{2-} site, $O(2)^{2-}$ is bonded in a

tetrahedral geometry to one $Li(1)^{1+}$, one $Li(4)^{1+}$, one $Al(2)^{3+}$, and one $Al(4)^{3+}$ atom. In the third O^{2-} site, $O(3)^{2-}$ is bonded in a distorted trigonal non-coplanar geometry to one $Li(4)^{1+}$, one $Zr(1)^{4+}$, and one $Al(3)^{3+}$ atom. In the fourth O^{2-} site, $O(4)^{2-}$ is bonded in a 3-coordinate geometry to one $Al(1)^{3+}$, one $Al(3)^{3+}$, and one $Al(4)^{3+}$ atom. There are two inequivalent Cl^{1-} sites. In the first Cl^{1-} site, $Cl(1)^{1-}$ is bonded in a distorted trigonal pyramidal geometry to one $Li(1)^{1+}$, one $Li(2)^{1+}$, one $Li(3)^{1+}$, and one $Li(4)^{1+}$ atom. In the second Cl^{1-} site, $Cl(2)^{1-}$ is bonded in a 3-coordinate geometry to one $Li(4)^{1+}$, one $Zr(1)^{4+}$, and one $Al(3)^{3+}$ atom.

References

[1] N. S. Bjørner, C. Eisenhofer, L. Kovács, Satisfiability Modulo Custom Theories in Z3, in *Verification, Model Checking, and Abstract Interpretation - 24th International Conference, VMCAI 2023, Boston, MA, USA, January 16-17, 2023, Proceedings*, Verification, Model Checking, and Abstract Interpretation, Springer **2023** pages 91–105.

[2] S. A. Cook, The complexity of theorem-proving procedures, in *Proceedings of the Third Annual ACM Symposium on Theory of Computing*, Proceedings of the ACM Symposium on Theory of Computing, Association for Computing Machinery **1971** pages 151–158.

[3] J. P. M. Silva, K. A. Sakallah, GRASP - a new search algorithm for satisfiability, in *Proceedings of the 1996 International Conference on Computer-Aided Design*, Proceedings of the International Conference on Computer-Aided Design, IEEE Computer Society / ACM **1996** pages 220–227.

[4] B. Deng, P. Zhong, K. Jun, J. Riebesell, K. Han, C. J. Bartel, G. Ceder, *Nature Machine Intelligence* **2023**, *5*, 1031–1041.

[5] G. Kresse, J. Furthmüller, *Physical Review B* **1996**, *54*, 11169.

[6] J. P. Perdew, K. Burke, M. Ernzerhof, *Physical Review Letters* **1996**, *77*, 3865.

[7] J. Sun, A. Ruzsinszky, J. P. Perdew, *Physical Review Letters* **2015**, *115*, 036402.

[8] J. Sun, R. C. Remsing, Y. Zhang, Z. Sun, A. Ruzsinszky, H. Peng, Z. Yang, A. Paul, U. Waghmare, X. Wu, M. L. Klein, J. P. Perdew, *Nature Chemistry* **2016**, *8*, 831.

[9] S. P. Ong, W. D. Richards, A. Jain, G. Hautier, M. Kocher, S. Cholia, D. Gunter, V. L. Chevrier, K. A. Persson, G. Ceder, *Computational Materials Science* **2013**, *68*, 314.

[10] D. Zagorac, H. Müller, S. Ruehl, J. Zagorac, S. Rehme, *Journal of Applied Crystallography* **2019**, *52*, 918.

[11] A. Jain, S. P. Ong, G. Hautier, W. Chen, W. D. Richards, S. Dacek, S. Cholia, D. Gunter, D. Skinner, G. Ceder, K. A. Persson, *APL Materials* **2013**, *1*, 011002.

[12] J. Gamon, M. S. Dyer, B. B. Duff, A. Vasylenko, L. M. Daniels, M. Zanella, M. W. Gaultois, F. Blanc, J. B. Claridge, M. J. Rosseinsky, *Chemistry of Materials* **2021**, *33*, 8733.

[13] K. Momma, F. Izumi, *Journal of Applied Crystallography* **2011**, *44*, 1272.

[14] A. M. Ganose, A. Jain, *MRS Communications* **2019**, *9*, 874.
